# COX‐2 in Fracture Callus Chondro‐Osseous Junction Osteoclasts Regulates Chondrocyte Hypertrophy and Callus Vasculogenesis

**DOI:** 10.1002/jor.70040

**Published:** 2025-08-03

**Authors:** Marc Teitelbaum, Hsuan‐Ni Lin, Maya D. Culbertson, Charlene Wetterstrand, J. Patrick O'Connor

**Affiliations:** ^1^ Department of Orthopaedics Rutgers‐New Jersey Medical School Newark New Jersey USA; ^2^ Rutgers‐School of Graduate Studies Newark Health Sciences Campus Newark New Jersey USA; ^3^ Eli Lilly and Company New York New York USA

**Keywords:** chondroclast, cyclooxygenase‐2, endochondral ossification, fracture healing, osteoclast

## Abstract

Cyclooxygenase‐2 (COX‐2) activity is necessary for bone fracture healing to proceed normally. COX‐2 is encoded by *Ptgs2* and is expressed by several cell types during fracture healing, suggesting that COX‐2 regulates multiple processes to affect fracture healing. Here, the role of COX‐2 expression in osteoclasts during mouse femur fracture healing was examined. Mice lacking COX‐2 (*Ptgs2*‐cKO^*Lyz2*^) in osteoclasts and other myeloid cells were made using a floxed COX‐2 gene (*Ptgs2* ^tm1Hahe^) and cre recombinase expressed from the *Lyz2* ^tm1(cre)If^° allele. Fracture healing was assessed by radiology, histology, immunohistochemistry, and mRNA quantification. Targeted loss of COX‐2 in osteoclasts was confirmed by immunohistochemistry and led to significant reductions in callus osteoclasts. Comparisons between *Ptgs2*‐cKO^*Lyz2*^ and control mice found significant reductions in callus chondrogenesis and bone formation in the *Ptgs2*‐cKO^*Lyz2*^ mice. The reductions were accompanied by delayed callus vascularization and reduced MMP‐13 expression. Immunohistochemistry showed that osteoclasts along the callus chondro‐osseous junction normally express COX‐2. In *Ptgs2*‐cKO^*Lyz2*^ mice, COX‐2 expression was reduced in osteoclasts at the chondro‐osseous junction and coincided with reduced MMP‐13 expression at the chondro‐osseous junction. The results indicate that COX‐2 expressed by osteoclasts along the chondro‐osseous junction promotes vasculogenesis and regulates chondrocyte hypertrophy during endochondral ossification. The results also indicate that osteoclasts at the callus chondro‐osseous junction coordinate multiple cellular processes to promote endochondral ossification.

## 1 Introduction

Cyclooxygenase‐2 (COX‐2) function is necessary for normal fracture healing [1, 2]. Null mutation of the mouse COX‐2 gene (*Ptgs2*) but not the COX‐1 gene (*Ptgs1*) significantly impairs fracture healing [3]. Further, nonsteroidal anti‐inflammatory drugs (NSAIDs), which inhibit COX‐2, can also impair fracture healing in multiple animal models [4, 5]. Retrospective clinical studies also support the role of COX‐2 in promoting human fracture healing [6, 7]. In addition, NSAIDs are used therapeutically to reduce heterotopic bone formation that often occurs after certain fractures [8]. Despite the importance of COX‐2 in bone healing, the mechanisms by which COX‐2 regulates fracture healing and bone regeneration are still poorly understood.

COX‐2 is necessary for synthesizing prostaglandin lipid mediators that regulate several physiological processes, including the inflammation response to tissue injury [3, 9]. COX‐2 produces prostaglandin H_2_ (PGH_2_) from arachidonic acid, which is converted into different lipid mediators (PGD_2_, PGE_2_, PGF_2α_, PGI_2_, or thromboxane A_2_) by specific synthases [9]. Prostaglandins are rapidly inactivated by enzymatic and nonenzymatic modifications and therefore function as local signaling molecules [10]. Prostaglandins signal through prostaglandin‐specific G‐coupled receptors to regulate intracellular cAMP and calcium levels [11].

The prostaglandins produced by COX‐2, particularly PGE_2_, have additional roles in skeletal biology. In vitro, PGE_2_ can promote both osteoclastogenesis and osteoblastogenesis [2, 12]. Systemic treatment with PGE_2_ or PGE_1_ (an analog of PGE_2_) can induce ectopic bone formation in humans and animals [13, 14]. Mechanical stress‐induced bone remodeling relies upon cyclooxygenase activity and several studies have shown that stress induces osteoblast COX‐2 expression in vitro [15, 16].

To elucidate the molecular mechanisms through which COX‐2 regulates fracture healing, we identified when and in which cells during fracture healing COX‐2 is expressed [17, 18]. COX‐2 expression was detected in callus chondrocytes, osteoblasts, and osteoclasts including those osteoclasts at the callus chondro‐osseous junction. Measurement of fracture callus prostaglandin levels found that PGE_2_ was the most abundant and that prostaglandin synthesis continues past the initial inflammatory phase and into the regenerative phase of fracture healing [19]. The results indicate a role for COX‐2 in fracture healing beyond regulating inflammation.

COX‐2 expression in osteoclasts, chondrocytes, and osteoblasts suggests one or more of these cell types may be necessary for fracture healing to proceed normally. Here, we determined the effects of targeted COX‐2 deletion in osteoclasts on femur fracture healing in mice. Our analysis indicates that osteoclast COX‐2 expression promotes callus vascularity and the progression of callus chondrocyte hypertrophy. The proximity of chondrocyte hypertrophy, vasculogenesis, and osteoclasts (also called chondroclasts) at the callus chondro‐osseous junction suggests that COX‐2 expressed in the chondro‐osseous junction osteoclasts regulates both processes during endochondral bone formation.

## 2 Materials and Methods

### 2.1 Animal Models

Mice in which exons 4 and 5 of the COX‐2 gene (*Ptgs2*) were flanked with loxP sequences (*Ptgs2* ^tm1Hahe^) were obtained from Jackson Laboratory (strain 030785; Bar Harbor, ME) [20]. Mice that express cre recombinase from the *Lyz2* locus (*Lyz2* ^tm1(cre)If^°) were obtained from Jackson Laboratory (strain 004781) [21]. Insertion of cre recombinase into the *Lyz2* locus produced an allele that expresses cre but no lysozyme. The *Ptgs2* ^tm1Hahe^ and *Lyz2* ^tm1(cre)Ifo^ mice were bred to produce mice that were COX‐2 null in myeloid cells (*Ptgs2*‐cKO^*Lyz2*^), including osteoclasts. C57BL/6 mice (wild‐type; Jackson Laboratory) and mice homozygous for the *Lyz2* ^tm1(cre)Ifo^ allele (*Lyz2* ^cre/cre^) were used as controls. All mice were maintained in the C57BL/6 background. Mice were genotyped via allele‐specific PCR amplification on DNA extracted from tail clip biopsies (SI Table S1). Mice were euthanized at 7, 10, 14, or 21 days postfracture (dpf) for histological and radiographic examination. Male and female mice of each genotype were used in all experiments. Male mice weighed significantly more than females for all genotypes (*p* < 0.001; SI Table S2). *Ptgs2*‐cKO^*Lyz2*^ mice (27.68 ± 3.51 g) weighed significantly more than the *Lyz2* ^cre/cre^ (22.96 ± 3.17 g) or WT (23.33 ± 3.47 g) mice (*p* < 0.001, both genotypes). All animal procedures were approved by the Rutgers‐New Jersey Medical School Institutional Animal Care and Use Committee (IACUC protocol 201800006).

### 2.2 Fracture Procedure and Radiography

Closed femur fractures were made in mice using previously described procedures [22]. Briefly, male and female mice aged 15 weeks were anesthetized by intraperitoneal injection of ketamine and xylazine (0.1 and 0.01 mg/g body weight, respectively) before retrograde insertion of a 0.01‐inch diameter stainless steel pin in the right femoral canal to stabilize the impending fracture. At 16 weeks of age, the mice were anesthetized again and the right femur was fractured using a three‐point controlled impactor. Radiographs were taken of anesthetized mice using an XPERT80 digital radiography cabinet (65 kV, 90 µA, and 8 s; KUBTEC, Stratford, CT) immediately after fracture and then at 7, 10, 14, and 21 days after fracture (dpf) or until the endpoint (SI Figure S1). Resected femurs were scanned using a high‐resolution computerized tomography (µCT) system (Bruker Skyscan 1275, Micro Photonics Inc., Allentown, PA) at 73 kVp, 133 µA intensity, 12 μm isotropic voxel, and with a 0.5 mm aluminum filter. The µCT images were reconstructed (NRecon), analyzed (CTan), and viewed (CTvol) using software from the manufacturer to determine fracture callus total volume (TV) and fracture callus calcified tissue volume (BV) as previously described [23].

### 2.3 Histology

Resected femurs were fixed in an aqueous solution of bronopol (3% w/v), diazolidinyl urea (3% w/v), zinc sulfate hepta‐hydrate (1.2% w/v), sodium citrate (0.29% w/v), ascorbic acid (0.025% w/v), and 20% (v/v) ethanol for 2 days at room temperature. The specimens were decalcified with 0.5 M Na_2_EDTA (2 weeks at 4^o^C), embedded in paraffin, cut into 5 µm thick longitudinal sections parallel to the intramedullary canal in the mediolateral plane, mounted onto TruBond 380 slides, deparaffinized, and rehydrated before further analysis. Osteoclasts were identified based on immuno‐histochemical detection of cathepsin K (CTSK), cell size, and multi‐nucleation. Cartilage was visualized by safranin‐O staining with fast green and hematoxylin counterstaining. Bone was visualized using Masson's trichrome.

### 2.4 Immunohistochemistry (IHC)

Antibodies, antibody dilutions, and antigen retrieval methods are listed in SI Table S3. Endogenous peroxidases were quenched with 3% H_2_O_2_ in PBS (30 min, room temperature) followed by nonspecific epitope blocking with SuperBlock (1 h, room temperature, ThermoFisher, Waltham, MA). After washing with PBS, diluted primary antibody (150 µL) was applied to each histological section and incubated overnight in a humidified chamber at 4^o^C. Following PBS washes, primary antibodies were detected using POLINK‐2 Plus Rabbit polymeric HRP secondary antibody (IHC World) and diaminobenzidine (GBI Labs, Bothell, WA). Sections were counterstained with methyl green.

### 2.5 Histology and Immunohistochemistry Image Collection and Analysis

Digital images of callus fracture sections were captured using an Olympus BX53 microscope and DP73 camera (Olympus Corporation of America, Center Valley, PA). Cartilage and bone areas were measured for each specimen using OsteoMeasure software (OsteoMetrics Inc., Decatur, GA) from safranin‐O and trichrome stained sections, respectively, and normalized as a percentage of total callus area. CTSK positive cells were manually counted using OsteoMeasure and divided by callus area to calculate CTSK positive cell density (cells per mm^2^). Similar procedures for image collection and analysis were conducted with the IHC samples to determine COX‐2, MMP‐13, CD31, ColX, and F4/80 positive areas or cell numbers in the callus.

### 2.6 Fracture Callus RNA Isolation, cDNA Synthesis, and qPCR

Mouse femur fracture calluses resected at 10 and 14 days after fracture (dpf) were used to isolate callus total RNA as described previously using Trizol extraction and Qiagen RNeasy purification [18]. Target mRNA levels were measured using RT‐qPCR with primers described in SI Table S1. At least three PCR reactions were performed with each cDNA preparation for every target mRNA and the mean threshold cycle (Ct) value was used for further comparisons. For each target mRNA, at least six callus RNA preparations (three male and three female) were measured for each genotype and timepoint. Target mRNA levels were normalized to the β‐actin mRNA cycle threshold (Ct) for the corresponding cDNA preparation using the 2^−ΔΔCt^ method [24].

### 2.7 Statistical Analyses

Statistical analyses were performed using OriginPro 2022b (OriginLab Corp., Northhampton, MA). Callus histomorphometry data, including cartilage area, bone area, and all immuno‐histochemical detection of cells or stained tissue area were analyzed by 3‐way ANOVA using genotype, days postfracture (dpf), and gender as independent variables. Post‐hoc tests utilized Tukey or Holm‐Sidak corrections to identify significant differences between groups. Detailed information from the statistical analyses is included in the Supporting Information (SI).

## 3 Results

### 3.1 COX‐2 Expression Is Reduced in Fracture Callus Osteoclasts of *Ptgs2*‐Cko^*Lyz2*^ Mice

**Figure 1:**
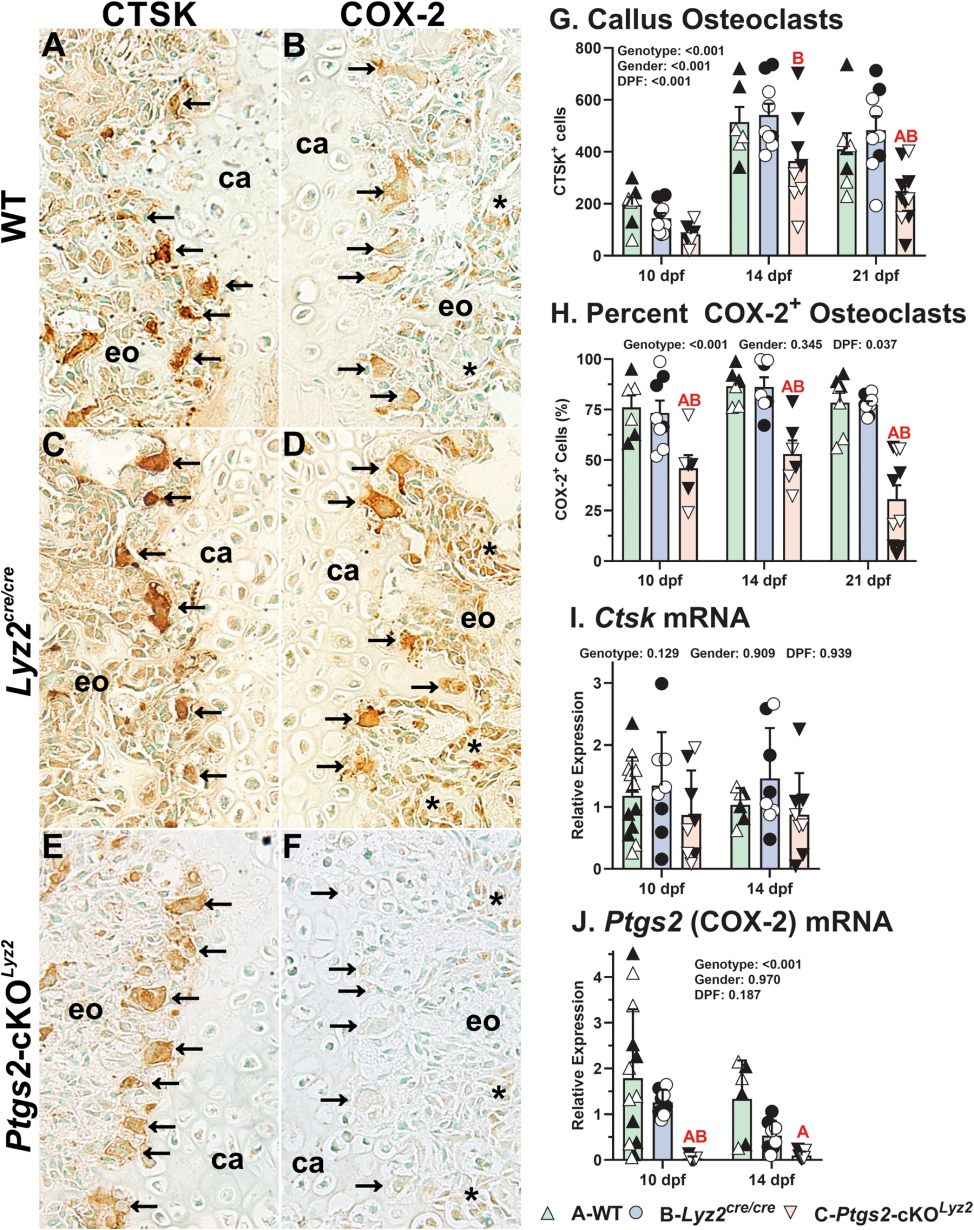
Lack of COX‐2 expression in *Ptgs2*‐cKO^*Lyz2*^ mouse femur fracture callus osteoclasts**.** Mouse femur fracture calluses collected at 14 dpf were used to detect CTSK (A, C, E) or COX‐2 (B, D, F) in WT (A and B), *Lyz2* ^cre/cre^ (C and D), and *Ptgs2*‐cKO^*Lyz2*^ (E and F) mice by immunohistochemistry. Shown are mirrored images from the callus chondro‐osseous junction serial sections of each mouse used to detect CTSK (A, C, E) and COX‐2 (B, D, F). Callus cartilage (ca) and the area of endochondral ossification (eo) are labeled. Arrows indicate osteoclasts along the chondro‐osseous junction. As expected, callus osteoclasts from WT and *Lyz2* ^cre/cre^ mice expressed both CTSK and COX‐2, while *Ptgs2*‐cKO^*Lyz2*^ osteoclasts expressed CTSK but showed reduced levels of COX‐2. Osteoblasts (*) expressing COX‐2 were observed in the WT, *Lyz2* ^cre/cre^, and *Ptgs2*‐cKO^*Lyz2*^ calluses. The number of callus cells expressing CTSK was significantly reduced in *Ptgs2*‐cKO^*Lyz2*^ mice at 14 dpf as compared to the *Lyz2* ^cre/cre^ mice and at 21 dpf as compared to both WT and *Lyz2* ^cre/cre^ mice (G). Gender effects on callus osteoclast number (*p* < 0.001) likely relates to callus size in male mice (closed symbols) as compared to female mice (open symbols). The number of osteoclasts expressing COX‐2 as determined by IHC was also reduced in the *Ptgs2*‐cKO^*Lyz2*^ calluses at all timepoints when normalized to the number of cells expressing CTSK (H). Consistent with the IHC observations, no differences were detected in *Ctsk* mRNA levels (I) while *Ptgs2* mRNA levels were significantly reduced in the *Ptgs2*‐cKO^*Lyz2*^ calluses (J). The effects of genotype, gender, and time after fracture were determined by 3‐way ANOVA and *p* values are shown in each graph. Statistically significant differences between genotypes within a time point are denoted with red letters in each graph (A: significantly different from WT; B: significantly different from *Lyz2* ^cre/cre^).

Immunohistochemistry was performed at 10, 14, and 21 dpf on fracture calluses from WT, *Lyz2* ^cre/cre^, and *Ptgs2‐*cKO^*Lyz2*^ mice to quantify the number of COX‐2 expressing osteoclasts. As shown at 14 dpf, osteoclasts expressing cathepsin K (CTSK) were localized along the callus chondro‐osseous junction in mice of all genotypes (Figure 1A,C,E). Consistent with the expected targeted deletion of *Ptgs2*, COX‐2 expression was absent or appeared at reduced levels only in osteoclasts from *Ptgs2*‐cKO^*Lyz2*^ mice (Figure 1B,D,F, arrows). In contrast, COX‐2 expression was evident in callus osteoblasts of all three genotypes (Figure 1B,D,F, asterisks).

Callus osteoclasts identified by CTSK expression increased from 10 to 14 or 21 dpf for all genotypes (Figure 1G). The increase in callus osteoclasts for male mice likely reflected their larger callus size since no gender difference was detected when osteoclasts were normalized to callus area (*p* = 0.65; SI Figure S1B). However, callus osteoclasts were reduced in *Ptgs2*‐cKO^*Lyz2*^ mice by 43% and 52% at 21 dpf as compared WT or *Lyz2* ^cre/cre^ mouse calluses, respectively (Figure 1G) and were reduced at 14 and 21 dpf when normalized to callus area (SI Figure S1B). The number of callus osteoclasts visibly expressing COX‐2 were compared between mouse genotypes as a percentage of the total number of callus osteoclasts (Figure 1H). At each timepoint, the percentage of osteoclasts expressing COX‐2 was significantly reduced in the *Ptgs2*‐cKO^*Lyz2*^ mice as compared to WT or *Lyz2* ^cre/cre^ (*p* < 0.001; overall means of 42%, 91%, and 86% between genotypes, respectively). In contrast, callus *Ctsk* mRNA levels showed no difference between the genotypes (Figure 1I), while *Ptgs2* mRNA levels were significantly lower in the *Ptgs2*‐cKO^*Lyz2*^ calluses as compared to WT at 10 and 14 dpf (Figure 1J). Overall mean callus levels of *Ptgs2* mRNA were 1.65, 0.89, and 0.07 for the WT, *Lyz2* ^cre/cre^, and *Ptgs2*‐cKO^*Lyz2*^ mice, respectively (*p* < 0.001).

### 3.2 Targeted Deletion of COX‐2 From Callus Osteoclasts Impairs Callus Bone Formation

**Figure 2:**
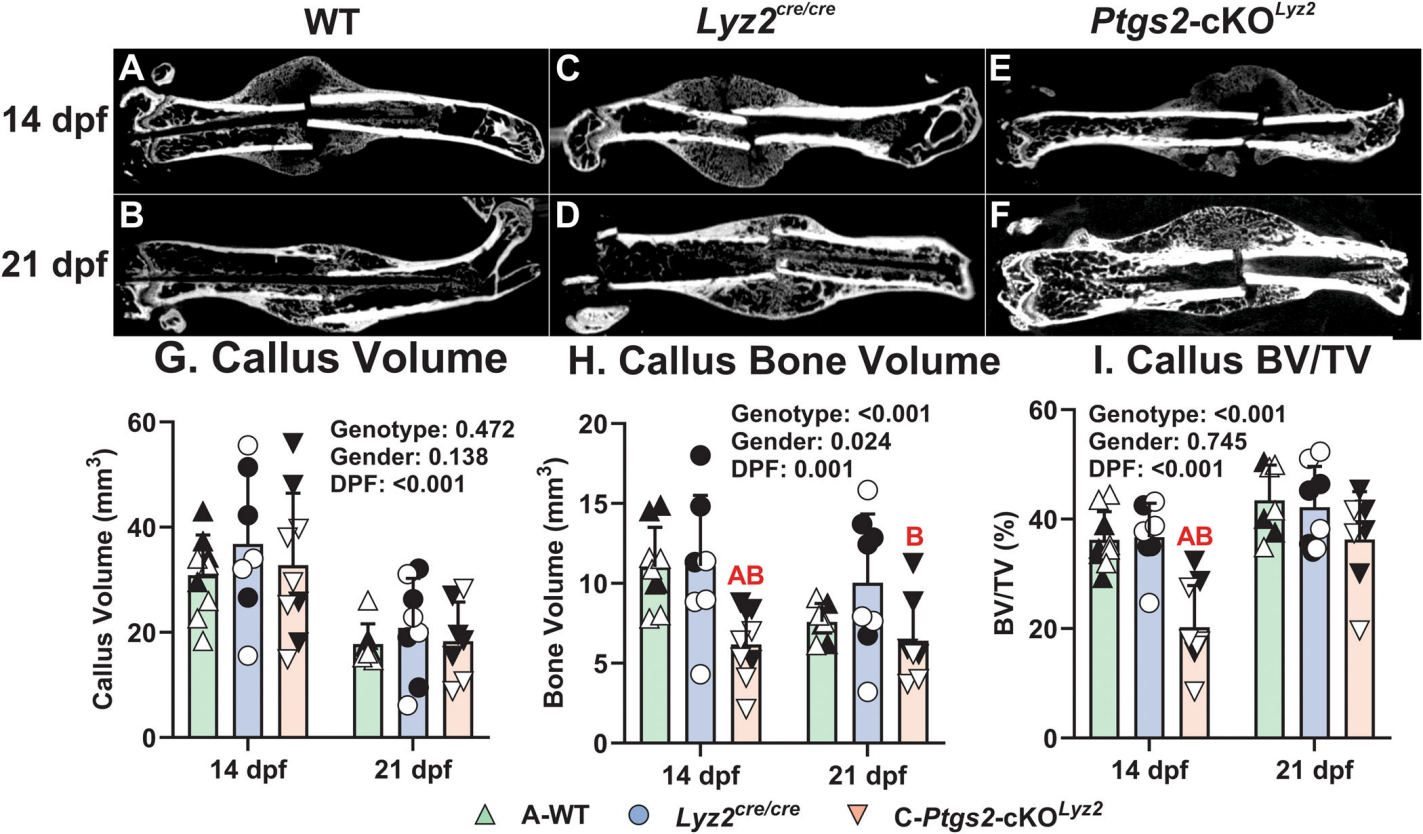
Delayed bone formation in *Ptgs2*‐cKO^*Lyz2*^ mouse femur fracture calluses**.** Fractured mouse femurs were collected from C57BL/6 (WT), *Lyz2* ^cre/cre^ and *Ptgs2*‐cKO^*Lyz2*^ mice and imaged by µCT scanning. Shown are representative images from the reconstructed scans at 14 and 21 dpf for WT (A, B), *Lyz2* ^cre/cre^ (C, D) and *Ptgs2*‐cKO^*Lyz2*^ mice (E, F), respectively. Callus total volume (TV; G), bone volume (BV; H) and normalized bone volume (BV/TV; I) were determined from µCT imaging data collected from at least three female (open symbols) and three male (closed symbols) mice of each genotype for each time point. The effects of genotype, gender, and time after fracture were determined by 3‐way ANOVA and *p* values are reported in each graph. Statistically significant differences between genotypes within a time point are denoted with red letters in each graph. Callus size decreased between 14 and 21 dpf but no effect of genotype or gender was detected (G). Callus BV was significantly greater in male mice as compared to female mice (H; *p* = 0.024) but this effect was absent when BV was normalized to callus volume (I; *p* = 0.745). Callus BV and BV/TV were significantly reduced in the *Ptgs2*‐cKO^*Lyz2*^ mice at 14 dpf (H and I).

Fractures from the WT and *Lyz2* ^cre/cre^ mice appeared to be bridged with new bone (calcified tissue) by 14 dpf with substantial callus bone remodeling evident by 21 dpf (Figure 2A–D). In contrast, by 14 dpf the *Ptgs2*‐cKO^*Lyz2*^ fractures were not fully bridged by bone and had large radiolucent areas within the callus (Figure 2E). By 21 dpf, the *Ptgs2*‐cKO^*Lyz2*^ calluses appeared to be bridged with bone but callus remodeling appeared to be delayed (Figure 2F). Quantification of callus volumes showed no genotype or gender differences (Figure 2G). However, callus bone volume and callus BV/TV were significantly reduced in the *Ptgs2*‐cKO^*Lyz2*^ mice at 14 dpf as compared to either the WT or *Lyz2* ^cre/cre^ mice (Figure 2H and I). Serial X‐rays of the healing fractures also indicated a delay in bone formation at 10 dpf in the *Ptgs2*‐cKO^Lyz2^ mice (SI Figure S2). Consistent with the reduced BV, overall mRNA levels for matricellular proteins, osteopontin (*Spp1*), osteonectin (*Sparc*), and periostin (*Postn*), were significantly reduced in the *Ptgs2*‐cKO^*Lyz2*^ mouse calluses relative to WT or *Lyz2* ^cre/cre^ though no significant differences were detected for osteocalcin (*Bglap*; SI Figure S1, Table S7, Statistical Analyses S7). Callus bone volume was greater in male mice (Figure 2H), but this effect disappeared when the bone volume was normalized to callus volume (BV/TV; Figure 2I).

### 3.3 Targeted Deletion of COX‐2 From Callus Osteoclasts Impairs Callus Chondrocyte Hypertrophy

**Figure 3:**
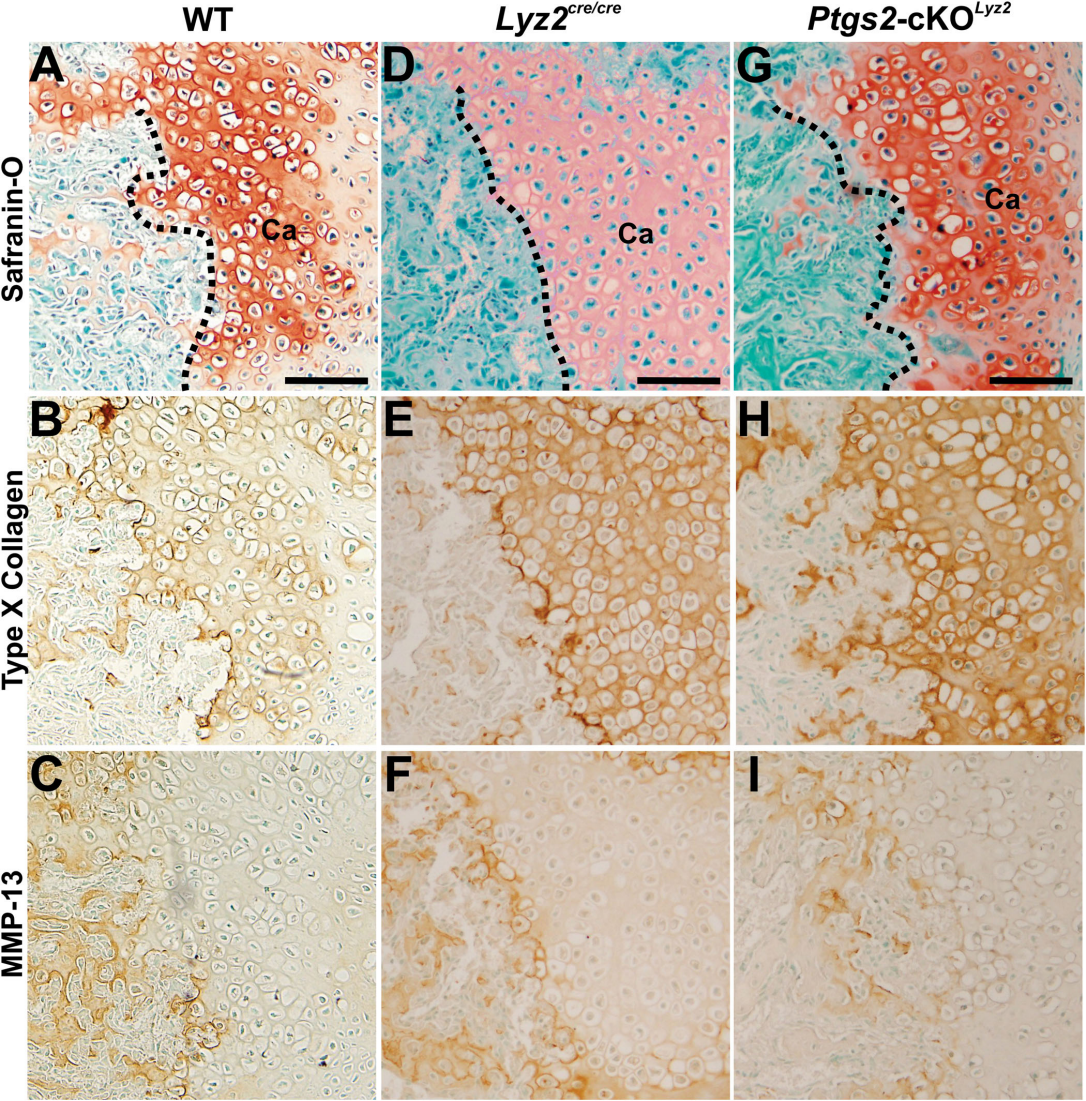
Reduced MMP‐13 expression in *Ptgs2*‐cKO^*Lyz2*^ mouse fracture callus chondrocytes**.** Longitudinal sections from 14 dpf mouse femur fracture calluses were stained with safranin‐O and fast green (A, D, G) or used to detect ColX (B, E, H) and MMP‐13 (C, F, I) by immunohistochemistry. Specimens were from WT (A, B, C), *Lyz2* ^cre/cre^ (D, E, F) and *Ptgs2*‐cKO^*Lyz2*^ (G, H, I) mice. Areas of callus cartilage (Ca) are noted in Safranin‐O stains along with the chondro‐osseous junction (dotted line). For all genotypes, ColX expression (brown; B, E, H) was prominently detected around callus cartilage chondrocytes but also around chondrocytes in areas of endochondral bone formation. In contrast, MMP‐13 expression (brown; C, F, I) was detected around chondrocytes in areas of endochondral ossification or those chondrocytes at the chondro‐osseous junction. MMP‐13 expression appeared reduced in the *Ptgs2*‐cKO^*Lyz2*^ callus (I). Scale bars equal 100 µm.

**Figure 4:**
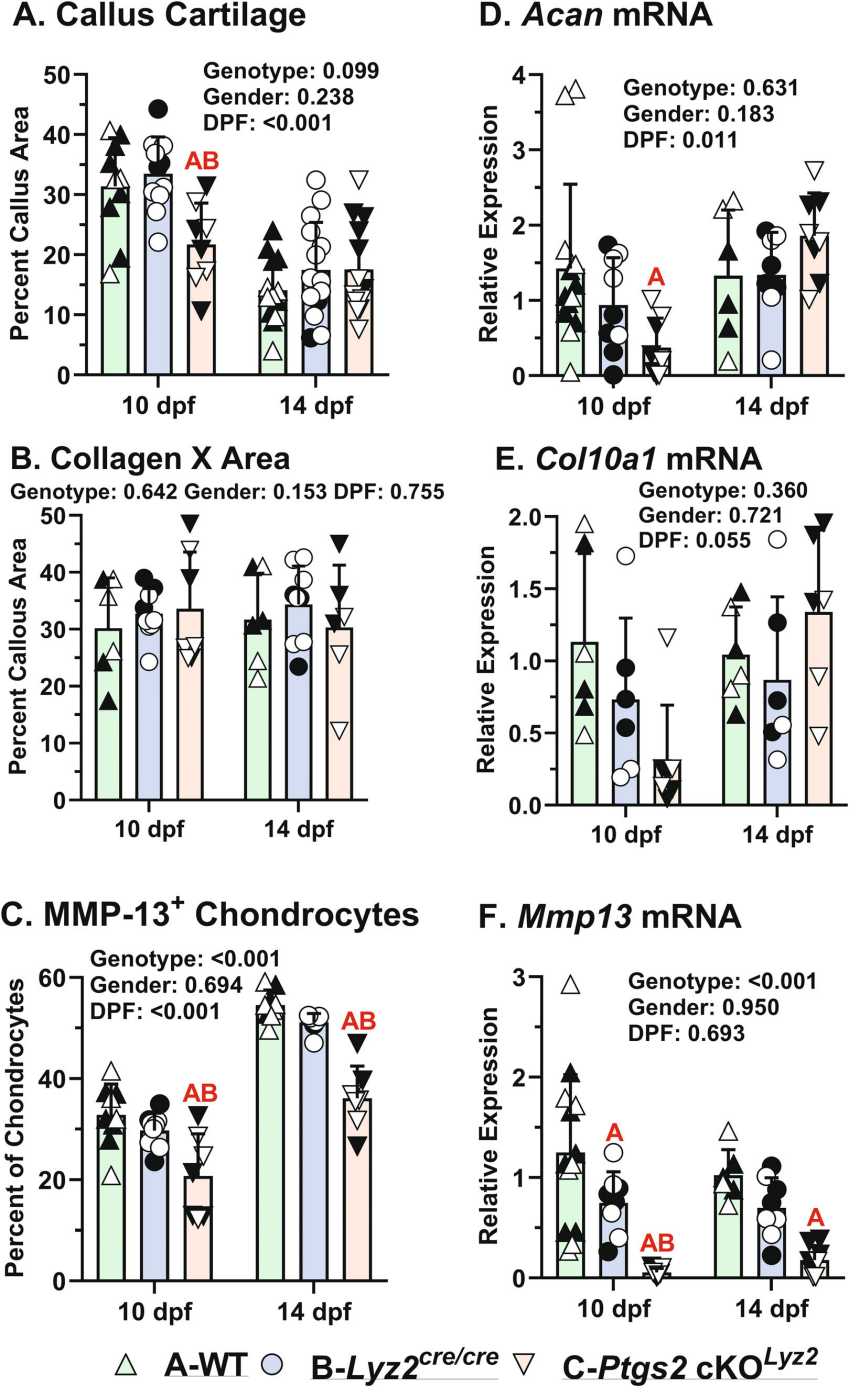
Aberrant callus chondrogenesis and hypertrophy in *Ptgs2*‐cKO^*Lyz2*^ mouse fracture calluses**.** Shown are the quantitative analyses of fracture callus chondrogenesis by histomorphometric analysis of cartilage area (A), ColX (B) and MMP‐13 (C) immunohistochemical staining, and RT‐qPCR measured levels of *Acan* (D), *Col10a1* (E), and *Mmp13* (F) mRNA levels. The effects of genotype, gender, and time after fracture were determined by 3‐way ANOVA and P‐values are reported in each graph. Statistically significant differences between genotypes within a time point are denoted with red letters in each graph. Time dependent changes between 10 and 14 dpf were found for reduced callus cartilage area (A) and increased percentage of MMP‐13 expressing chondrocytes (C). No differences were detected between WT and *Lyz2* ^cre/cre^ specimens or between males (closed symbols) and females (open symbols), except for a reduction in the *Lyz2* ^cre/cre^ callus *Mmp13* mRNA level at 10 dpf (F). In contrast, callus cartilage area (A), the percentage of MMP‐13 expressing chondrocytes (C), and *Acan* and *Mmp13* mRNA levels (D, F) were all reduced in the *Ptgs2*‐cKO^*Lyz2*^ calluses at 10 dpf. Consistent with the observed reduction in MMP‐13 as detected by immunohistochemistry, the percentage of MMP‐13 expressing chondrocytes (C) and *Mmp13* mRNA levels (F) were significantly reduced in the *Ptgs2*‐cKO^*Lyz2*^ calluses at 14 dpf.

Calluses cartilage from all mouse genotypes were stained with Safranin‐O (Figure 3A,D,G). Callus cartilage area and aggrecan (*Acan*) mRNA were significantly reduced in the *Ptgs2*‐cKO^*Lyz2*^ mice at 10 dpf when callus cartilage area normally peaks (Figure 4A,D) [22, 25]. Immunohistochemical localization of Type X Collagen and MMP‐13 at 14 dpf in the WT and *Lyz2* ^cre/cre^ mouse calluses showed that most callus chondrocytes expressed Type X collagen (Figure 3B,E) while MMP‐13 expression was localized only to chondrocytes at the chondro‐osseous junction and chondrocytes that had been traversed by the chondro‐osseous junction (Figure 3C,F). In contrast, the *Ptgs2*‐cKO^*Lyz2*^ callus chondrocytes also expressed Type X collagen (Figure 3H) but the apparent level of MMP‐13 expression was much lower in the *Ptgs2*‐cKO^*Lyz2*^ chondrocytes at the chondro‐osseous junction and the chondrocytes that had been traversed by the chondro‐osseous junction (Figure 3I). Quantification of Type X collagen and *Col10a1* mRNA levels found no differences between genotypes (Figure 4B,E). In contrast, proportionally fewer callus chondrocytes expressed MMP‐13 in the *Ptgs2*‐cKO^*Lyz2*^ mice at 10 and 14 dpf (Figure 4C). Similarly, *Mmp13* mRNA levels were low at 10 dpf and remained significantly lower relative to WT at 14 dpf (Figure 4F).

### 3.4 Fracture Callus Vascularization Is Delayed by Targeted Deletion of COX‐2 From Osteoclasts

**Figure 5:**
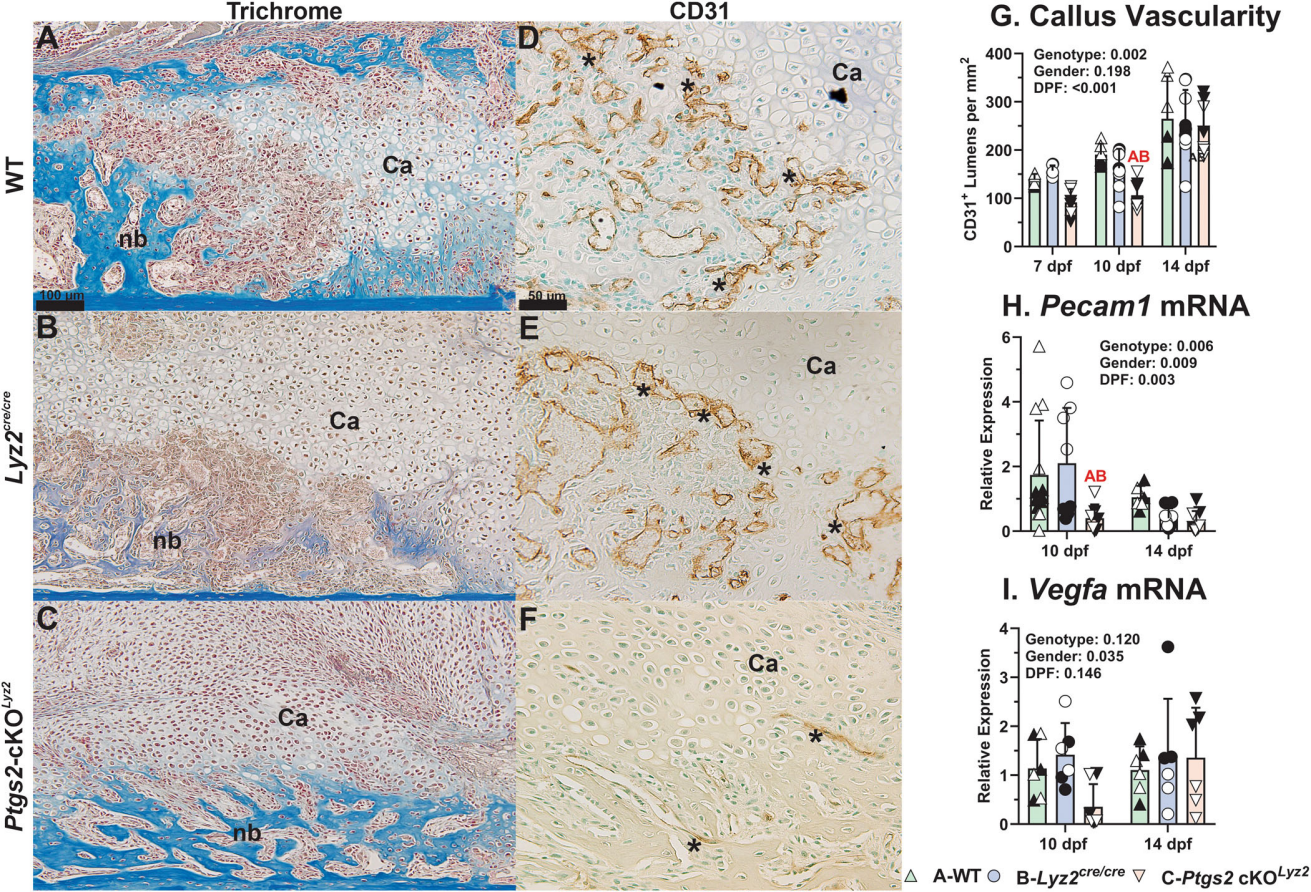
Delayed callus vasculogenesis in *Ptgs2*‐cKO^*Lyz2*^ mice**.** Shown are histological sections from 10 dpf calluses stained with Masson's trichrome (A B, C, scale bar 100 µm) or used to detect CD31 expressing endothelial cells by immunohistochemistry (D, E, F, scale bar 50 µm). Callus cartilage (Ca) and areas of newly formed bone in the calluses (nb) are noted in the WT (A), *Lyz2* ^cre/cre^ (B), and *Ptgs2*‐cKO^*Lyz2*^ (C) calluses. Vascular lumens (*) surrounded by CD31 expressing endothelial cells appeared abundant in the regions of newly formed bone and absent from callus cartilage in the WT (D) and *Lyz2* ^cre/cre^ (E) mouse calluses. In the *Ptgs2*‐cKO^*Lyz2*^ mice, CD31 expressing cells and vascular lumens (*) appeared less abundant in the 10 dpf callus (F). Histomorphometric analysis of the vascular lumens found an overall time dependent increase in callus vascularity for all genotypes (*p* < 0.001) but with a significant reduction in vascular density within the *Ptgs2*‐cKO^*Lyz2*^ callus at 10 dpf (G). Measurement of callus *Pecam1* (CD31) mRNA levels also found a significant reduction in the *Ptgs2*‐cKO^*Lyz2*^ calluses (H), though no significant effect on *Vegfa* mRNA levels was detected (I). The effects of genotype, gender, and time after fracture were determined by 3‐way ANOVA and *p* values are reported in each graph. Statistically significant differences between genotypes within a time point are denoted with red letters in each graph. Closed symbols represent male mice; open symbols represent female mice.

Vascularization of femur fracture calluses was determined by immunohistochemical detection of CD31 (*Pecam1*). Areas of newly formed bone (nb) and cartilage (Ca) were evident in all callus specimens at 10 dpf (Figure 5A–C). Vessels (lumens surrounded by CD31 expressing cells) in the WT and *Lyz2* ^cre/cre^ mouse fracture calluses were abundant by 10 dpf (Figure 5D,E). In contrast, fewer vessels appeared in the *Ptgs2*‐cKO^*Lyz2*^ calluses (Figure 5F). The number of vessels in fracture calluses at 7, 10, and 14 dpf were counted and normalized to callus area (Figure 5G). Vascular density was significantly lower in the *Ptgs2*‐cKO^*Lyz2*^ mouse calluses at 10 dpf but increased to a level similar to those observed in WT and *Lyz2* ^cre/cre^ mice by 14 dpf. *Pecam1* mRNA levels were also reduced in the *Ptgs2*‐cKO^*Lyz2*^ calluses at 10 dpf, consistent with the reduced vascular density (Figure 5H). Similar to previous reports, *Pecam1* and *Vegfa* mRNA levels were significantly elevated in the female fracture calluses (Figure 5H,I) [26, 27].

### 3.5 Effects of Targeted Deletion of COX‐2 on Callus Macrophages

**Figure 6:**
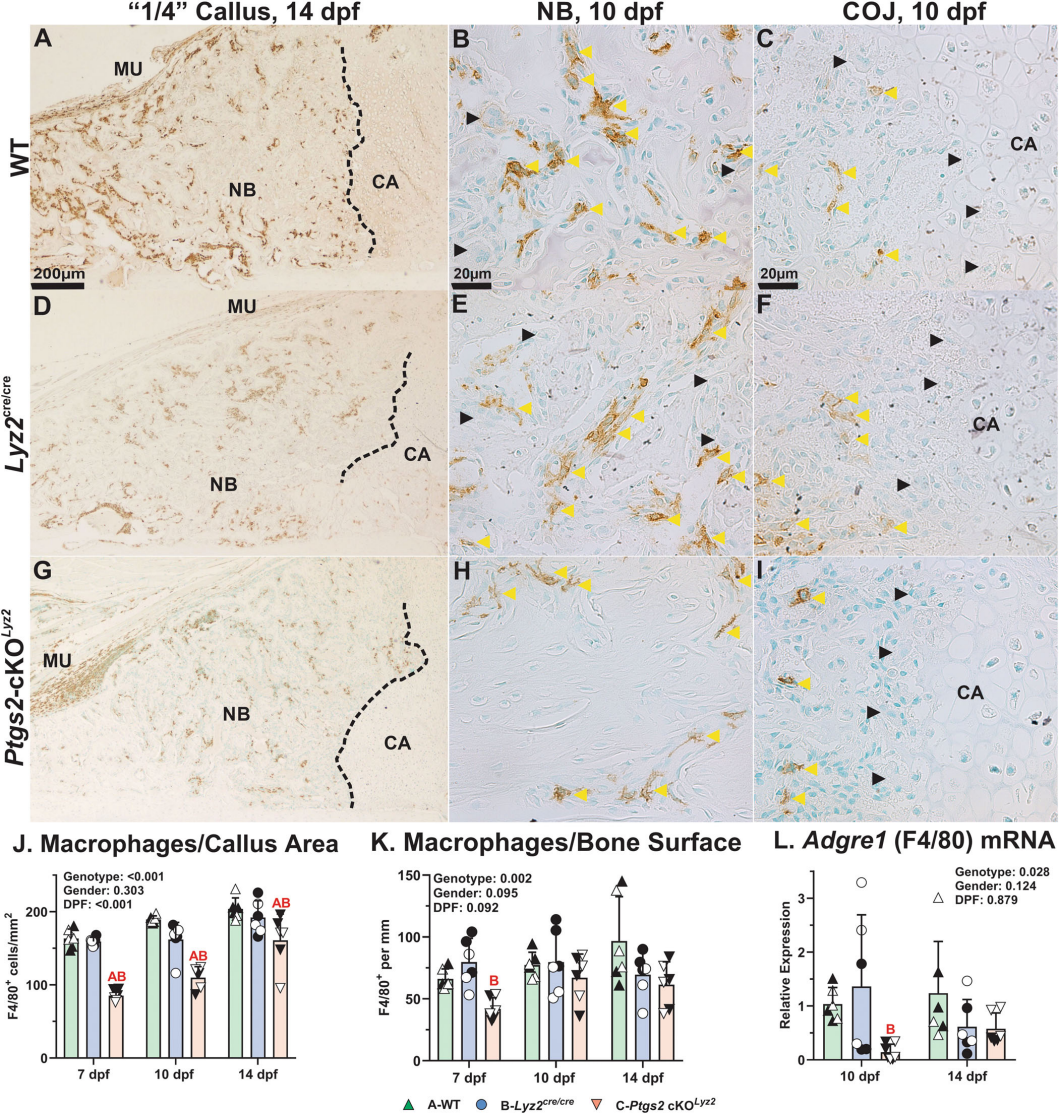
Comparison of callus macrophages in WT, *Lyz2* ^cre/cre^, and *Ptgs2*‐cKO^*Lyz2*^ mice**.** Immunohistochemistry was used to identify macrophages expressing F4/80 (*Adgre1*) in 7 (not shown), 10 (B, C, E, F, H, I, scale bar 20 µm), and 14 dpf (A, D, G, scale bar 200 µm) fracture calluses of WT (A, B, C), *Lyz2* ^cre/cre^ (D, E, F) and *Ptgs2*‐cKO^*Lyz2*^ mice (G, H, I). Muscle (MU), callus cartilage (CA), areas of newly formed bone in the callus (NB), and the chondro‐osseous junction (dotted line) are noted. At 14 dpf, F4/80 expressing macrophages were mainly detected in muscle at the callus periphery and in areas of callus new bone (A, D, G). At 10 dpf, macrophages (yellow arrowheads) in areas of callus new bone appeared associated with marrow in WT (B) and *Lyz2* ^cre/cre^ (E) calluses but appeared less abundant in the *Ptgs2*‐cKO^*Lyz2*^ callus (H). At the chondro‐osseous junction (C, F, I), more osteoclasts (black arrowheads) than macrophages appeared to be adjacent to the callus cartilage. Quantitative analyses found significant reductions in *Ptgs2*‐cKO^*Lyz2*^ macrophage density as compared to WT or *Lyz2* ^cre/cre^ at all timepoints (J). However, when normalized to callus new bone perimeter, no genotype‐dependent differences in macrophages per mm bone perimeter were detected (K). *Adgre1* (F4/80) mRNA levels were significantly reduced at the *Ptgs2*‐cKO^*Lyz2*^ calluses as compared to WT and *Lyz2* ^cre/cre^ mice across both time points (*p* = 0.028, L), but within a time point, *Adgre1* mRNA levels were only reduced at 10 dpf the *Ptgs2*‐cKO^Lyz2^ calluses as compared to the *Lyz2* ^cre/cre^ calluses. The effects of genotype, gender, and time after fracture were determined by 3‐way ANOVA and *p*values are reported in each graph. Statistically significant differences between genotypes within a time point are denoted with red letters in each graph. Closed symbols represent male mice; open symbols represent female mice.

Macrophages identified by IHC detection of F4/80 (*Adgre1*) were abundant in the femur bone marrow of all mouse genotypes (not shown). Macrophages were also abundant in the muscle surrounding the fracture callus as shown at 14 dpf in the WT, *Lyz2* ^cre/cre^, *Ptgs2*‐cKO^*Lyz2*^ and mice (Figure 6A,D,G). Within the callus, macrophages were reduced in the *Ptgs2*‐cKO^*Lyz2*^ mice (Figure 6J). However, the macrophages were predominantly localized at the newly formed bone (yellow arrowheads, Figure 6B,E,H) and when the callus macrophages were normalized to callus bone surface, no differences between WT and *Ptgs2*‐cKO^*Lyz2*^ were detected (Figure 6K). Consistent with the overall reduced number of macrophages in the *Ptgs2*‐cKO^*Lyz2*^ calluses, *Adgre1* mRNA levels were also significantly lower in the *Ptgs2*‐cKO^*Lyz2*^ calluses (ANOVA *p* = 0.028; Figure 6L). Macrophages expressing F4/80 were also observed at the callus chondro‐osseous junction (yellow arrowheads, Figure 6C,F,I). Unlike osteoclasts (black arrowheads), the macrophages were generally not located in direct contact with callus cartilage.

### 3.6 Targeted Deletion of COX‐2 From Callus Osteoclasts Does Not Prevent Healing

**Figure 7:**
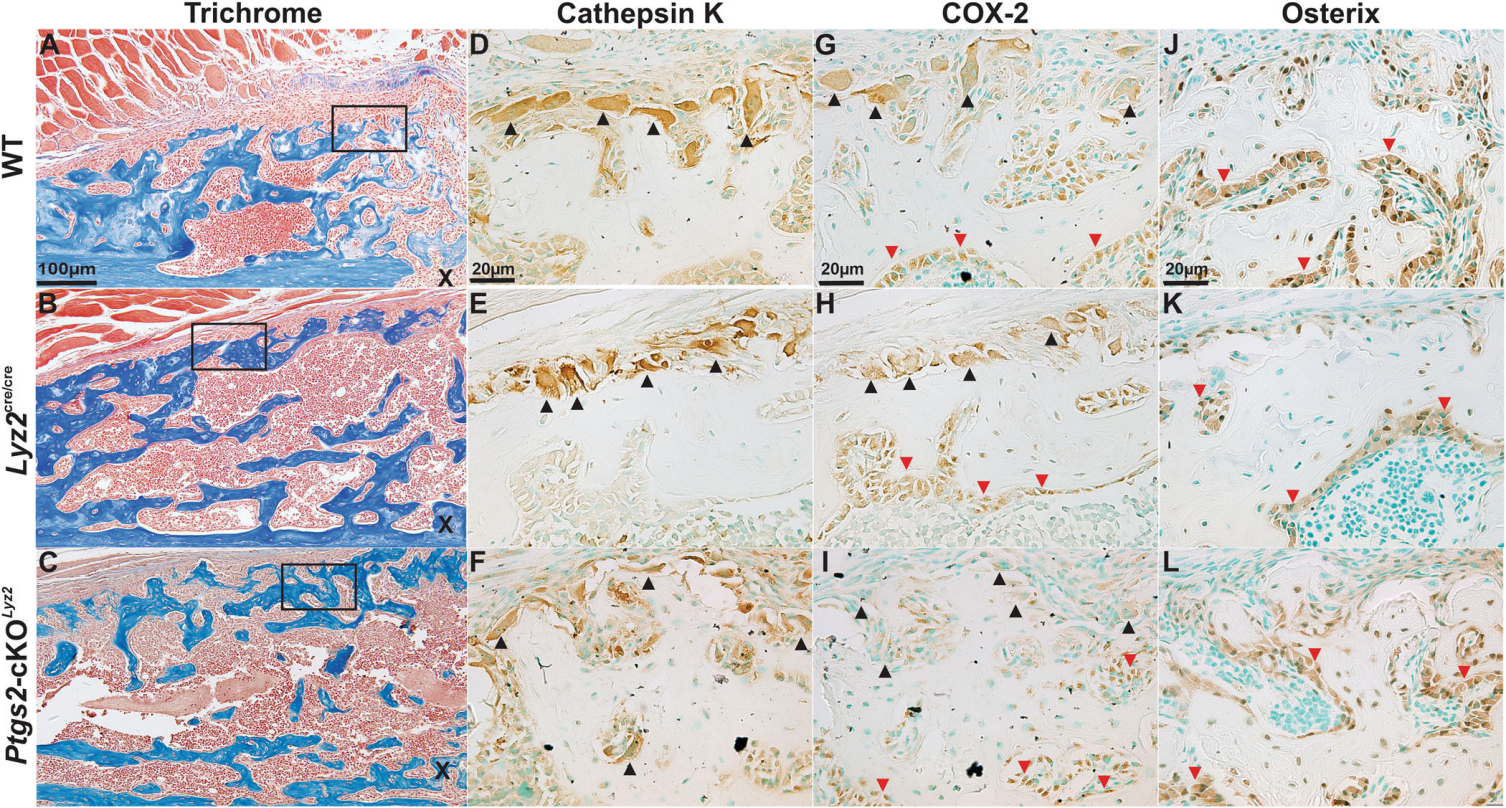
Callus bone formation in the absence of osteoclast COX‐2 expression**.** Longitudinal sections from 21 dpf mouse femur fracture calluses were stained with Masson's trichrome (A, B, C, scale bar 100 µm) or used to detect CTSK (D, E, F), COX‐2 (G, H, I), or Sp7 (J, K, L) by immunohistochemistry (scale bar 20 µm). Specimens were from WT (A, D, G, J), *Lyz2* ^cre/cre^ (B, E, H, K), and *Ptgs2*‐cKO^*Lyz2*^ (C, F, I, L) mice. The fracture site is located near the bottom right corner (X) of Masson's trichrome stained images. New bone formation in the callus (blue) is detected along the periosteum, within the callus, and at the muscle boundary for all genotypes (A, B, C). Osteoclasts (black arrowheads) expressing CTSK were abundant along the peripheral edges of newly formed bone next to the muscle boundary for all genotypes (D, E, F). Osteoblasts (red arrowheads) expressing COX‐2 (G, H, I) or Sp7 (J, K, L) were abundant in all genotypes. In contrast, osteoclasts (black arrowheads) expressing COX‐2 were only detected in the WT (G) and *Lyz2* ^cre/cre^ (H) calluses with reduced or absent COX‐2 expression in the *Ptgs2*‐cKO^*Lyz2*^ callus (I).

Loss of COX‐2 from osteoclasts using the *Lyz2* ^cre^ allele did not prevent bone formation in mouse femur fractures (Figure 1, SI Figure S2 and Tables S4–S6). New bone was evident in calluses of all genotypes at 21 dpf (Figure 7A–C). Osteoclasts expressing CTSK were abundant in calluses of all genotypes (Figure 7D–F). Similarly, osteoblasts expressing osterix (*Sp7*) were abundant in calluses of all genotypes (Figure 7J–L). However, while COX‐2 expression was evident in osteoblasts from all genotypes (compare Figure 7G–L), COX‐2 expression was absent or reduced in osteoclasts from the *Ptgs2*‐cKO^*Lyz2*^ mice (compare Figure 7F–I).

## 4 Discussion

Targeted deletion of COX‐2 in osteoclasts using the floxed *Ptgs2* ^tm1Hahe^ allele and the cre recombinase expressing *Lyz2* ^tm1(cre)Ifo^ allele (*Ptgs2*‐cKO^*Lyz2*^) was confirmed by immunohistochemistry (Figure 1). Loss of COX‐2 from osteoclasts delayed but did not prevent fracture healing (Figures 2 and 7). Delayed fracture healing in the *Ptgs2*‐cKO^*Lyz2*^ mice was associated with a reduction of callus osteoclasts (Figure 1), delayed bone formation (Figure 2), reduced callus cartilage (Figure 4), reduced MMP‐13 expression (Figures 3 and 4), and reduced or delayed vasculogenesis of the callus (Figure 5). An overall reduction in callus macrophages was detected in the *Ptgs2*‐cKO^*Lyz2*^ mice but when macrophages were normalized to callus bone, no difference was detected (Figure 6).

*Ptgs2* ^null^ mice are viable with normally formed skeletons [28]. However, fracture healing is severely impaired in *Ptgs2* ^null^ mice, indicating that COX‐2 is necessary for fracture healing but not essential for bone formation [1, 2]. Similarly, *Ptgs2*‐cKO^*Lyz2*^ mice have normally formed skeletons. Unlike *Ptgs2* ^null^ mice though, fracture healing was successful in the *Ptgs2*‐cKO^*Lyz2*^ mice, albeit with the abnormalities listed above. Bone formation in mouse femur fracture calluses occurs via endochondral ossification at the chondro‐osseous junction and also by apparent intramembranous ossification at the peripheral edges of the callus and along the callus muscle boundary (Figure 7). Loss of COX‐2 in osteoclasts did not appear to affect bone formation at the callus muscle boundary, (which may have enabled healing; Figure 7), or prevent callus bone remodeling (Figure 2).

Loss of *Ptgs2* from all cells, from osteoclasts (this study), from chondrocytes, or from osteoblast, chondrocytes, and mesenchymal cells, did not grossly affect skeletal formation or growth [1, 2, 29]. However, all caused abnormalities in cartilage formation or resolution of callus cartilage into bone during fracture healing. *Ptgs2* ^null^ fracture calluses are small, composed mainly of chondrocytes, and have impaired bone formation [1, 2]. Targeted deletion of *Ptgs2* using the *Prx1*‐cre transgene (Tg(*Prrx1*‐cre)^1Cjt^) reduced callus bone and increased callus cartilage and mesenchymal cells at 14 dpf [29]. Targeted deletion of *Ptgs2* using cre expressed from the *Col2a1* promoter (*Col2*‐cre) also reduced callus bone and increased callus cartilage and mesenchymal cells at 14 dpf [29]. However, targeted deletion of *Ptgs2* using the *Lyz2* ^tm1(cre)^ allele reduced callus cartilage at 10 dpf and callus bone at 14 dpf (Figures 2 and 4). This suggests that unlike the targeted deletion of *Ptgs2* with *Prx1*‐cre or *Col2*‐cre, targeted deletion with *Lyz2* ^tm1(cre)^ may be affecting earlier events during fracture healing that reduces chondrocyte differentiation. That these earlier events may be mediated by COX‐2 expressed from macrophages or other myeloid‐derived cells requires additional investigation. During endochondral ossification, a cartilage anlage is replaced with bone. This typically occurs in a progressive process that includes: (a) chondrocyte hypertrophy; (b) cartilage matrix calcification, resorption, and vascularization; (c) osteoblast‐mediated bone formation on remnants of calcified cartilage; (d) remodeling of the newly formed bone; and (e) establishment of bone marrow [30].

A critical phase of endochondral ossification is progression of chondrocytes into hypertrophy, which is characterized by *Col10a1* and *Mmp13* expression [30]. Immunohistochemical detection of ColX showed that callus chondrocytes rapidly progress from proliferating chondrocytes into hypertrophic chondrocytes expressing *Col10a1* (Figure 3) [18]. However, *Mmp13* expression was not detected until the callus chondro‐osseous junction came in contact with the *Col10a1* expressing hypertrophic chondrocytes (Figure 3). Thus, MMP‐13 expression in the fracture callus appeared restricted to only those chondrocytes at the chondro‐osseous junction or those chondrocytes that had been traversed by the chondro‐osseous junction. Progression from ColX^+^ hypertrophic chondrocytes to ColX^+^, MMP‐13^+^ hypertrophic chondrocytes was dramatically reduced in the *Ptgs2*‐cKO^*Lyz2*^ mouse calluses when COX‐2 was absent from chondro‐osseous junction osteoclasts (Figures 3 and 4). Failure to convert into ColX^+^, MMP‐13^+^ hypertrophic chondrocytes appears to be one reason for delayed bone formation in *Ptgs2*‐cKO^Lyz2^ mouse calluses.

Since COX‐2 is expressed by multiple cell types (Figure 7) [17], prior experiments using systemic NSAIDs cannot distinguish cell type specific roles for COX‐2 during fracture healing. However, prior studies using a rat femur fracture model found that systemic NSAIDs promoted abnormal morphology of callus cartilage and impaired chondrocyte progression into hypertrophy based on reduced *Col10a1* expression [1, 31]. In vitro chondrocyte models as well as analysis of rabbit growth plates found that NSAIDs inhibited chondrocyte hypertrophy based on reduced *Col10a1*, *Mmp13*, and *Vegfa* expression and reductions in the length of growth plate hypertrophic zones [32, 33]. A comparison of tibia growth plates from wild‐type, celecoxib (COX‐2 selective NSAID) treated wild‐type, and *Ptgs2* ^null^ mice found that the growth plate proliferative zone was reduced only in the *Ptgs2* ^null^ mice, while ColX expression and hypertrophic chondrocytes were reduced in both the celecoxib‐treated and *Ptgs2* ^null^ mice [34]. As shown here, callus chondrocyte hypertrophy appears to be regulated by osteoclast COX‐2 activity.

Co‐localization of callus vascularization and chondrocyte hypertrophy at the chondro‐osseous junction suggests that these two processes may be mutually dependent upon osteoclast COX‐2 activity. Vascular invasion of cartilage precedes bone formation during fetal endochondral ossification and is necessary for fracture healing [35, 36]. However, in the *Ptgs2*‐cKO^*Lyz2*^ mouse calluses, vascularization of the callus was delayed at 10 dpf (Figure 5). In *Ptgs2* ^null^ mouse fractures, reduced callus vasculogenesis and delayed resolution of callus cartilage into bone was reversed by local infusion of either prostaglandin E_2_ (PGE_2_) or a prostaglandin receptor EP4 agonist (CP‐734432) [37]. Interestingly, the increased vasculogenesis and bone formation induced in the *Ptgs2* ^null^ callus by PGE_2_ or CP‐734432 was accompanied by increased *Mmp9* expression. Osteoclasts express *Mmp9* and mice lacking *Mmp9* have abnormal growth plates and fracture calluses characterized by reduced vasculogenesis and delayed resolution of hypertrophic chondrocytes [38]. The available experimental results suggest that COX‐2 in osteoclasts acts through an autocrine or paracrine mechanism to induce *Mmp9* expression and thereby indirectly promote callus vasculogenesis.

An additional possibility is that osteoclast COX‐2 activity is necessary for septoclast activity at the chondro‐osseous junction. Septoclasts are mononuclear cells that appear to be derived from mesenchymal cells [39, 40]. Septoclasts contact and resorb hypertrophic chondrocytes while expressing cathepsin B, MMP‐9, and MMP‐13 [41]. Thus, the reduced MMP‐13 expression in the *Ptgs2*‐cKO^*Lyz2*^ calluses may also be related to COX‐2 dependent signaling between osteoclasts at the chondro‐osseous junction and septoclasts.

VEGF expression is another hallmark of chondrocyte hypertrophy [30, 36]. Given the reduced conversion of ColX^+^ chondrocytes to ColX^+^, MMP‐13^+^ chondrocytes in the *Ptgs2*‐cKO^*Lyz2*^ mouse calluses, the reduced vascularity may be a function of fewer hypertrophic chondrocytes producing VEGF. The level of *Vegfa* mRNA appeared to be reduced in the *Ptgs2*‐cKO^*Lyz2*^ calluses at 10 dpf, but the reduction was not statistically significant (Figure 5I). PGE_2_ and other COX‐2 derived products can promote angiogenesis [42, 43]. Thus, another possibility is that prostaglandins produced by chondro‐osseous junction osteoclasts help promote vascularization.

The experiments shown here were performed with mice that were homozygous for the floxed *Ptgs2* ^tm1Hahe^ allele as well as *Lyz2* ^tm1(cre)Ifo^ allele [20, 21]. In the *Lyz2* ^tm1(cre)Ifo^ allele, exon 1 of *Lyz2* is replaced with cre recombinase having an N‐terminal nuclear localization sequence. As such, the *Lyz2* ^tm1(cre)Ifo^ allele does not express lysozyme. Consequently, mice that were homozygous only for the *Lyz2* ^tm1(cre)Ifo^ allele were used as controls. The only significant effect detected was that *Mmp13* mRNA levels were reduced in the *Lyz2* ^cre/cre^ calluses relative to WT at 10 dpf but not at 14 dpf (Figure 4).

The deficits in fracture healing observed in the *Ptgs2*‐cKO^*Lyz2*^ mice were largely interpreted as being caused by loss of COX‐2 activity in osteoclasts, particularly those osteoclasts (chondroclasts) at the callus chondro‐osseous junction. The *Lyz2* ^tm1(cre)Ifo^ allele enables cre‐mediated recombination in osteoclasts as well as other myeloid cells including macrophages [21]. Thus, the potential contributions of *Lyz2* ^tm1(cre)Ifo^ mediated inactivation of *Ptgs2* in other cell types on the impaired fracture healing observed in the *Ptgs2*‐cKO^*Lyz2*^ mice cannot be ruled out. COX‐2 expression was observed in callus osteoblasts (Figures 1 and 7) and chondrocytes (not shown) of the *Ptgs2*‐cKO^*Lyz2*^ mice indicating some specificity of the *Lyz2* ^tm1(cre)Ifo^ allele. However, the reduction of callus cartilage at 10 dpf in the *Ptgs2*‐cKO^*Lyz2*^ mice (Figure 4A) suggests that loss of COX‐2 in macrophages, osteal macrophages, or another myeloid cell population could be influencing earlier events of fracture healing. Macrophages and tissue‐resident osteal macrophages have been shown to positively affect bone regeneration and fracture healing [44, 45]. Whether and to what extent loss of COX‐2 in macrophages or osteal macrophages has on fracture healing could not be distinguished from the loss of COX‐2 in osteoclasts as described here. Based on F4/80 immunoreactivity, callus macrophages were sparse within cartilage, sparse at the chondro‐osseous junction, but were coincident with new vasculature (Figure 6). Accordingly, we favor the proposed interpretation of the results that osteoclast COX‐2 expression affects chondrocyte hypertrophy and vasculogenesis. The reduction in callus cartilage and delay in callus remodeling in *Ptgs2*‐cKO^*Lyz2*^ calluses parallels prior studies showing that pharmacological depletion of callus osteoclasts does not prevent bony union but delays resolution of callus cartilage into bone and delays callus remodeling [46, 47]. Reduced osteoclastogenesis caused by inhibition of RANK signaling in mice also leads to delayed resolution of callus cartilage and impaired callus remodeling, in addition to osteopetrosis [48]. Loss of *Tnfrsf11a* (RANK) in mice dramatically reduces osteoclastogenesis, callus vascularity, and the incidence of bony union, again indicating a role for osteoclasts in callus vasculogenesis and fracture healing [48]. The overlap in observed effects upon fracture healing between targeted loss of COX‐2 in osteoclasts and the chemical or genetic depletion of osteoclast suggests that COX‐2 has a pivotal function in osteoclasts during fracture healing.

COX‐2 expression is controlled by physiological regulation of transcription factors, by posttranscriptional control of mRNA stability, and by post‐translational regulation of COX‐2 activity [49]. Mice that are homozygous for a null mutation in the osteopontin gene (*Spp1*) or in the periostin gene (*Postn*) also have deficiencies in fracture healing that include reduced percentages of MMP‐13 expressing chondrocytes and reduced callus size [18]. Interestingly, the *Spp1* ^null^ and *Postn* ^null^ mice also showed reduced expression of COX‐2 in osteoclasts, including those osteoclasts at the callus chondro‐osseous junction. In the *Ptgs2*‐cKO^*Lyz2*^ mouse fracture calluses, the levels of *Spp1*, *Postn*, and *Sparc* mRNAs were reduced (SI Figure S1, Table S7, and Statistical Analyses S7). *Spp1*, *Postn*, and *Sparc* are matricellular proteins expressed by osteoblasts and so the reduced mRNA levels in the *Ptgs2*‐cKO^*Lyz2*^ calluses may simply reflect delayed bone formation. However, the results also suggest that osteopontin, periostin, and possibly other matricellular proteins regulate osteoclast COX‐2 expression to affect fracture healing and potentially other bone homeostatic mechanisms.

The biology of the callus chondro‐osseous junction is poorly understood. Using laser capture, Khan et al. collected and analyzed gene expression in mouse callus chondro‐osseous junction osteoclasts associated with cartilage (chondroclasts) as well as callus osteoclasts associated with bone [50]. The analysis found 375 differentially expressed genes upregulated in chondroclasts as compared to 357 upregulated genes in osteoclasts. A secondary analysis indicated that *Etv1*, *Sirt1*, and *Atf1* likely regulate the chondroclast specific gene expression profile. Targeted deletion of *Runx1* using the *Lyz2* ^tm1(cre)Ifo^ allele increased callus chondroclasts at 14 dpf in male mice while also reducing the bone formation rate [51]. Whether chondroclasts and osteoclasts originate from different precursor cells, utilize different signaling pathways to promote differentiation into chondroclasts versus osteoclasts, or whether the differential gene expression observed by Khan et al. represents osteoclasts responding to different microenvironments remain open questions. More clear is that the present results support a regulatory role for osteoclasts in fracture healing that includes dependence on COX‐2 expression.

## Author Contributions

Marc Teitelbaum performed experiments, aided in experimental design and methods, collected and analyzed data, and helped prepare the manuscript. Hsuan‐Ni Lin performed experiments, aided in experimental design and methods, and collected and analyzed data. Maya D. Culbertson performed experiments, maintained mouse colonies, and aided with manuscript preparation. Charlene Wetterstrand performed experiments, developed methods, and collected and analyzed data. J. Patrick O'Connor developed the study, aided in experimental design and methods, performed data analysis, and prepared the manuscript. All authors reviewed and approved the submitted manuscript.

## Supporting information


**Supporting Figure S1:** Loss of COX‐2 in Osteoclasts Effects Osteogenic mRNA Levels in Ptgs2‐ cKOLyz2 mice.
**Supporting Figure S2:** Serial X‐ray Observations of Femur Fracture Healing in Ptgs2‐cKOLyz2 Mice.
**Supporting Table S1:** Oligodeoxynucleotides used in this study.
**Supporting Table S2:** Mouse body weights at fracture (16 weeks old).
**Supporting Table S3:** Rabbit antibodies used in this study.
**Supporting Table S4:** Summary of histomorphometry data.
**Supporting Table S5:** Summary of Callus IHC data.
**Supporting Table S6:** Summary of μCT data.
**Supporting Table S7:** Summary of relative mRNA level data.
