# Supplementary material for: COX‐2 in Fracture Callus Chondro‐Osseous Junction Osteoclasts Regulates Chondrocyte Hypertrophy and Callus Vasculogenesis
Source: J Orthop Res. 2025 Aug 3;43(11):1973–86. doi: 10.1002/jor.70040 (PMC12509252; doi:10.1002/jor.70040)
Supplement: Supplementary file 1 — Supporting Figure S1: Loss of COX‐2 in Osteoclasts Effects Osteogenic mRNA Levels in Ptgs2‐ cKOLyz2 mice. Supporting Figure S2: Serial X‐ray Observations of Femur Fracture Healing in Ptgs2‐cKOLyz2 Mice. Supporting Table S1: Oligodeoxynucleotides used in this study. Supporting Table S2: Mouse body weights at fracture (16 weeks old). Supporting Table S3: Rabbit antibodies used in this study. Supporting Table S4: Summary of histomorphometry data. Supporting Table S5: Summary of Callus IHC data. Supporting Table S6: Summary of μCT data. Supporting Table S7: Summary of relative mRNA level data. [file JOR-43-1973-s001.pdf]

## Supplemental Information

# Table of Contents

## Figures

Figure S1. Loss of COX-2 in Osteoclasts Effects Osteogenic mRNA Levels in *Ptgs2*-cKO$^{Lyz2}$ mice……………………………………………………………………………………….3

Figure S2. Serial X-ray Observations of Femur Fracture Healing in *Ptgs2*-cKO$^{Lyz2}$ Mice……....4

## Tables

Table S1. Oligodeoxynucleotides used in this study………………………..…………………5

Table S2. Mouse body weights at fracture (16 weeks old)……………………………………..6

Table S3: Rabbit antibodies used in this study…..…………..……………………………………6

Table S4. Summary of histomorphometry data……..………………….…………………………7

Table S5. Summary of Callus IHC data……………………………………………………..8

Table S6. Summary of µCT data………………………...……………………………………..9

Table S7. Summary of relative mRNA level data…..……………………………………...10

## Statistical Analyses

S1-A. Histomorphometry: Total TRAP$^+$ Callus Cells………………………………………..12

S1-B. COX-2 IHC Percent COX-2$^+$ Osteoclasts: (Percent Callus COX-2$^+$ OCs/TRAP$^+$OCs)....17

S1-C. Relative *Ctsk* mRNA Levels (RTqPCR)………………………………………………….22

S1-D. Relative COX-2 (*Ptgs2*) mRNA Levels (RTqPCR)……………………………………...25

S2-A. µCT Analysis: Callus Volume (mm$^3$)……………………………………………………….28

S2-B. µCT Analysis: Callus Bone Volume (mm$^3$) ………………………………………………...31

S2-C. µCT Analysis: BV/TV (%)………………………………………………………………….34

S3-A. Chondrocyte Figure- No Statistical Analyses…………………………………………37

S4-A. Histomorphometry: Callus Percent Cartilage Area: (Cartilage Area/Callus Area)………38

S4-B. Cartilage IHC Callus Percent COL10A1: (COL10A1 area/Callus Area)………………..41

S4-C.Cartilage IHC Percent MMP13$^+$ Chondrocytes: (MMP13$^+$ Cells/No. of Chondrocytes)....44

S4-D. Relative Aggrecan (*Acan)* mRNA Levels (RTqPCR)……………………….……………47

S4-E. Relative *COL10A1* mRNA Levels (RTqPCR)…..……………………………………..50

S4-F. Relative *Mmp13* mRNA Levels (RTqPCR)………………………………...……..…53

S5-A. CD31 IHC Callus Lumen Density: (CD31$^+$ Lumens per callus mm$^2$)…………….……..56

S5-B. Relative CD31 (*Pecam1*) mRNA Levels (RTqPCR)……………………………………..61

S5-C. Relative *Vegfa* mRNA Levels (RTqPCR)………………………………………………...64

S6-A. F4-80+ Macrophage Density: (F4-80$^+$ cells per callus mm$^2$).…………………………....67

S6-B. F4-80+ Macrophage EO Zone Density: (F4-80$^+$ cells per Bone Perimeter mm)………...71

S6-C. Relative F4/80 (*Adgre1*) mRNA Levels (RTqPCR)……………………………………...75

S7-A. Histomorphometry Callus Percent Bone: (Bone Area/Callus Area)…….………...……..78

S7-B. Relative Osteocalcin (*Bglap*) mRNA Levels (RTqPCR)………………….………...……83

S7-C. CTSK$^+$ Osteoclast Density: (CTSK$^+$ cells per callus mm$^2$)…………………..…..…………86

S7-D. Osteopontin (*Spp1*) mRNA Levels (RTqPCR)……………………………….…………..91

S7-E. Osteonectin (*SPARC*) mRNA Levels (RTqPCR)…………………………….…………...94

S7-F. Periostin (*Postn*) mRNA Levels (RTqPCR)………………………………………..……...97

**Figure S1. Loss of COX-2 in Osteoclasts Effects Osteogenic mRNA Levels in *Ptgs2*-cKO$^{Lyz2}$ mice.** Histomorphometry and RT-qPCR measurements of callus mRNA levels were used to assess osteogenesis and osteoclastogenesis in femur fracture calluses of WT, *Lyz2*$^{cre/cre}$, and *Ptgs2*-cKO$^{Lyz2}$ mice. Statistical significance for genotype, gender, and days post-fracture (dpf) were determined by 3-way ANOVA and P-values are reported in each panel (A–F). Significant differences within a timepoint are shown with red letters as A: different from WT and B: different from *Lyz2*$^{cre/cre}$. Panel A: Histomorphometric analysis of new bone formation in fracture calluses was performed using Masson's Trichrome staining. *Ptgs2*-cKO$^{Lyz2}$ mice exhibited significantly reduced new bone formation at 14 and 21 dpf. Panel B: The number of osteoclasts expressing cathepsin K (CTSK) were identified by immunohistochemistry and quantified relative to callus area. Significant reductions in osteoclast density were found in the *Ptgs2*-cKO$^{Lyz2}$ calluses at 14 and 21 dpf. Panels C, D, E and F: Respectively, the relative levels of *Bglap* (osteocalcin), *Spp1* (osteopontin), *Sparc* (osteonectin), and *Postn* (periostin) mRNAs were determined. No differences in *Bglap* mRNA levels were detected. Significant reductions in *Spp1*, *Postn*, and *Sparc* mRNAs that encode matricellular proteins were detected in the *Ptgs2*-cKO$^{Lyz2}$ calluses relative to WT or *Lyz2*$^{cre/cre}$. Closed symbols represent male mice; open symbols, female mice.

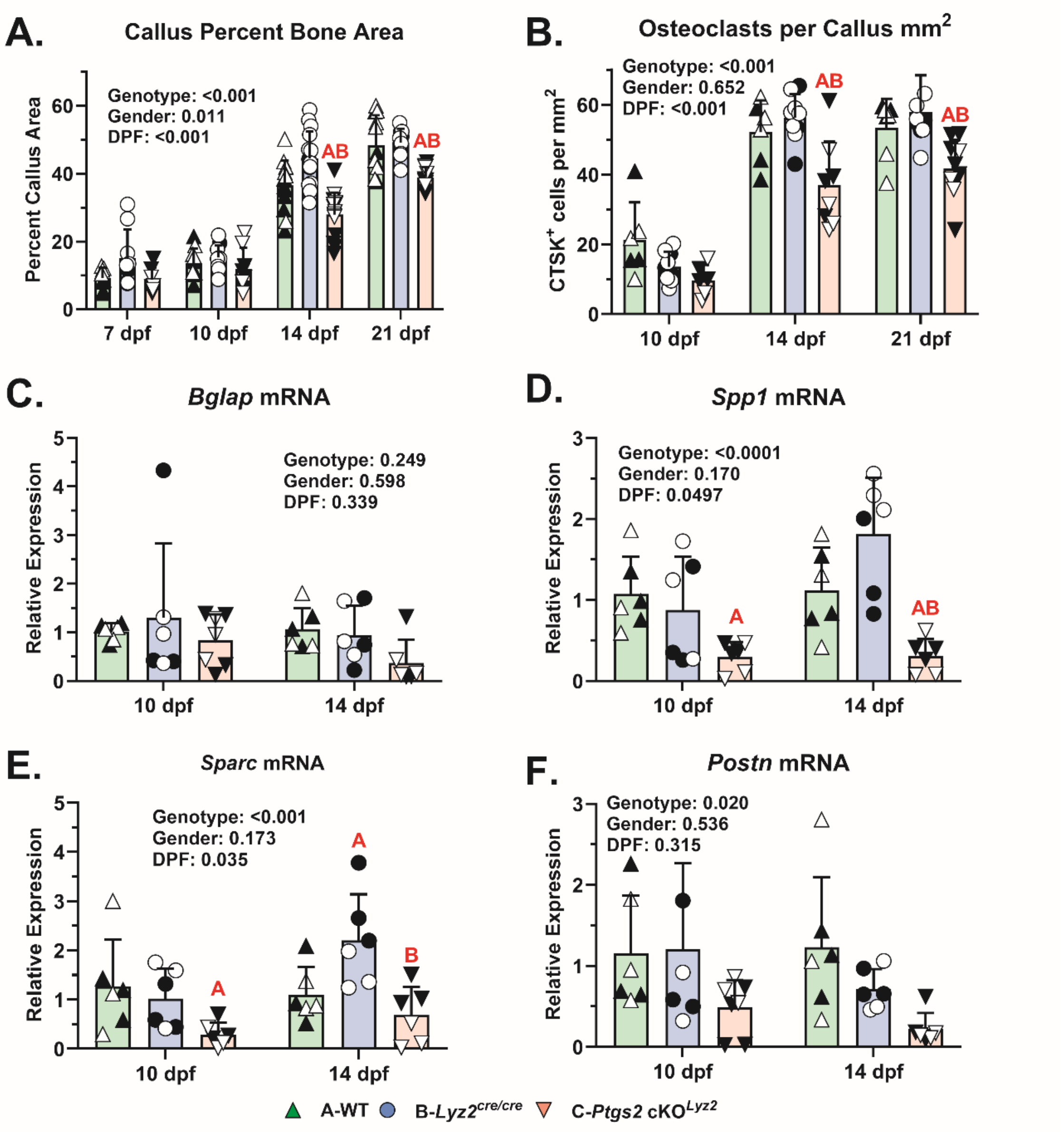

**Figure S2. Serial X-ray Observations of Femur Fracture Healing in *Ptgs2*-cKO*Lyz2* Mice.** Fracture healing was observed in C57BL/6 (WT), *Lyz2*$^{cre/cre}$, and *Ptgs2*-cKO$^{Lyz2}$ mice by X-ray radiography using an XPERT80 digital radiography cabinet (KUBTEC, Stamford, CT). The XPERT80 was used on auto-exposure (65 kV, 90 µA, cumulative 8 second exposure). Shown are serial images of femur fracture healing in a WT, *Lyz2*$^{cre/cre}$, and two *Ptgs2*-cKO$^{Lyz2}$ mice at 7, 10, 14, and 21 dpf. Grayscale-inverted images from the same radiographs are shown below.

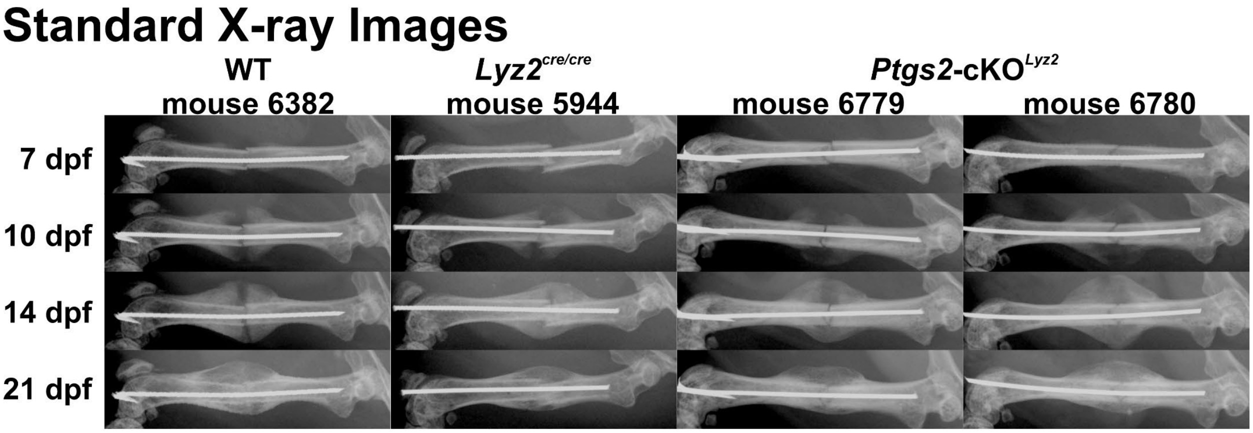


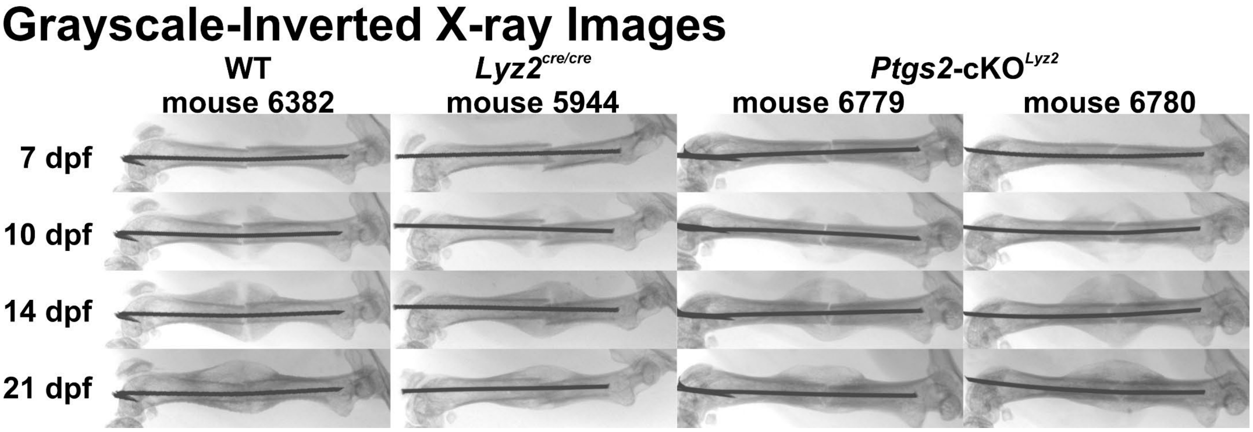

## Table S1. Oligodeoxynucleotides used in this study

| Genotyping Primers | | | |
|---|---|---|---|
| **Designation** | **Gene Allele** | **Forward** | **Reverse** |
| floxed *Ptgs2* | *Ptgs2*$^{tm1Hahe}$ | GGTCCCTCGAAGAGGTTCACT | GCGCAGTTTATGTTGTCTGTCC |
| wild-type *Ptgs2* | *Ptgs2* | AATTACTGCTGAAGCCCACC | CTTCCCAGCTTTTGTAACCAT |
| *Lyz2*$^{cre}$ | *Lyz2*$^{tm1(cre)Ifo}$ | GCAGCATTGCAGACTAGCTAAAGG | CCTGGCGATCCCTGAACATGTCC |
| wild-type *Lyz2* | *Lyz2* | TACCTTTGCTGGGCAAGAG | GTCCCTGTTCTGAGCGATTAG |
| **RTqPCR Primers** | | | |
| **Gene** | **Protein** | **Forward** | **Reverse** |
| *Acan* | Aggrecan | CATGAGAGAGGCGAATGGAA | TGATCTCGTAGCGATCTTTCTTCT |
| *Acp5* | Tartrate-resistant Acid Phosphatase 5 | GGTTCCAGGAGACCTTTGAG | TTCCAGCCAGCACATACC |
| *Actb* | ß-actin | GGGCTATGCTCTCCCTCACG | AGACGAACATAGCACAGCTTCTCTT |
| *Adgre1* | F4-80 | CTCTGTGGTCCCACCTTCAT | GATGGCCAAGGATCTGAAAA |
| *B2m* | ß-2-microglobulin | CTGCTACGTAACACAGTTCCACCC | CATGATGCTTGATCACATGTCTCG |
| *Bglap* | Osteocalcin | CCATGAGGACCATCTTTCTGC | CAGGTCCTAAATAGTGATACC |
| *Col10a1* | Collagen X | TCATGCCTGATGGCTTCATA | CAGCCTACTGCTGGGTAAGC |
| *Ctsk* | Cathepsin-K | AGGCAGCTAAATGCAGAGGGTACA | AGCTTGCATCGATGGACACAGAGA |
| *Mmp13* | Matrix Metallopeptidase-13 | GAGTGCCTGATGTGGGTGAAT | CCAGAAGGTCCATCAAATGGGT |
| *Pecam1* | CD-31 | CCAAAGCCAGTAGCATCATGGTC | GGATGGTGAAGTTGGCTACAGG |
| *Postn* | Periostin | CCTGCCCTTATATGCTCTGCT | AAACATGGTCAATAGGCATCACT |
| *Ptgs2* | Cyclooxygenase-2 | GGGCAGGAAGTCTTTGGTC | GGTAACCGCTCAGGTGTTG |
| *Sparc* | Osteonectin | CACCTGGACTACATCGGACCAT | CTGCTTCTCAGTGAGGAGGTTG |
| *Spp1* | Osteopontin | GCAGCCATGAGTCAAGTCAGC | GCCTCTTCTTTAGTTGACC |
| *Vegfa* | Vascular endothelial growth factor | ATCTTCAAGCCGTCCTGTGT | GCATTCACATCTGCTGTGCT |

**Table S2. Mouse body weights at fracture (16 weeks old)**

| Genotype | Gender | N | Mean Weight (g) | SD (g) | P vs. | |
|---|---|---|---|---|---|---|
| | | | | | WT | *Lyz2*$^{cre/cre}$ |
| WT | Male | 30 | 26.13 | 2.47 | ___ | |
| WT | Female | 29 | 20.45 | 1.27 | | |
| *Lyz2*$^{cre/cre}$ | Male | 25 | 25.48 | 2.1 | 0.659 | ___ |
| *Lyz2*$^{cre/cre}$ | Female | 26 | 20.38 | 1.68 | | |
| *Ptgs2*-cKO$^{Lyz2}$ | Male | 20 | 29.23 | 3.1 | <0.001 | <0.001 |
| *Ptgs2*-cKO$^{Lyz2}$ | Female | 21 | 25.9 | 2.95 | | |

**Table S3. Rabbit antibodies used in this study**

| Target | Company | Catalog Number | Type | Dilution | Antigen Retrieval |
|---|---|---|---|---|---|
| CTSK (cathepsin K) | Novus Biologicals | NBP1-45460 | PAb | 1:100 | 10 mM Na•citrate 1 hour, 75°C |
| CD-31 (Pecam-1) | Cell Signaling | 77699 | MAb | 1:500 | 10 mM Na•citrate 1 hour, 75°C |
| Collagen X | abcam | ab260040 | MAb | 1:1,000 | 25 mg/ml hyaluronidase 1 hour, 37°C |
| COX-2 | Cayman | 160126 | PAb | 1:700 | 10 mM Na•citrate 1 hour, 75°C |
| F4/80 | Cell Signaling | 70076 | MAb | 1:1,000 | 10 mM Na•citrate 1 hour, 75°C |
| MMP-13 | abcam | ab219620 | MAb | 1:100 | 25 mg/ml hyaluronidase 1 hour, 37°C |
| Sp7 (Osterix) | abcam | ab209484 | MAb | 1:500 | 10 mM Na•citrate 1 hour, 75°C |

**Table S4. Summary of histomorphometry data**

| Day after Fracture | Genotype | | | | | |
|---|---|---|---|---|---|---|
| | C57BL/6 (WT) | | *Lyz2*$^{cre/cre}$ | | *Ptgs2*-cKO$^{Lyz2}$ | |
| | N | Mean (+/-) SD | N | Mean (+/-) SD | N | Mean (+/-) SD |
| **Fracture Callus Size (mm²)** | | | | | | |
| 7 | 8 | 7.28 ± 2.50 | 9 | 9.30 ± 6.90 | 7 | 7.20 ± 2.11 |
| 10 | 10 | 10.70 ± 5.88 | 11 | 10.27 ± 4.78 | 8 | 8.40 ± 2.43 |
| 14 | 13 | 9.73 ± 3.49 | 15 | 10.47 ± 3.72 | 13 | 8.96 ± 3.28 |
| 21 | 11 | 7.36 ± 2.27 | 10 | 8.04 ± 3.09 | 13 | 4.91 ± 2.38 |
| **Callus Percent Cartilage** | | | | | | |
| 10 | 10 | 31.38 ± 8.09 | 11 | 33.49 ± 6.11 | 8 | 21.73 ± 6.87 |
| 14 | 13 | 14.12 ± 5.51 | 15 | 17.47 ± 7.91 | 15 | 17.58 ± 7.69 |
| **Callus TRAP+ Cells** | | | | | | |
| 10 | 10 | 226.70 ± 110.65 | 11 | 126.36 ± 65.43 | 8 | 118.38 ± 51.94 |
| 14 | 13 | 508.08 ± 158.10 | 11 | 510.00 ± 150.76 | 14 | 331.43 ± 132.90 |
| 21 | 11 | 481.64 ± 168.54 | 10 | 485.60 ± 167.65 | 12 | 275.42 ± 63.13 |
| **Callus TRAP+ Cells per mm$^2$** | | | | | | |
| 10 | 10 | 21.57 ± 6.37 | 11 | 12.54 ± 3.92 | 8 | 15.02 ± 6.33 |
| 14 | 13 | 55.34 ± 6.47 | 11 | 52.18 ± 7.33 | 14 | 34.88 ± 7.30 |
| 21 | 11 | 62.87 ± 3.68 | 10 | 61.65 ± 9.39 | 12 | 45.92 ± 8.24 |
| **Callus Percent Bone** | | | | | | |
| 7 | 8 | 9.24 ± 3.04 | 9 | 15.08 ± 8.39 | 7 | 8.63 ± 3.68 |
| 10 | 10 | 13.55 ± 4.46 | 11 | 15.24 ± 3.56 | 8 | 11.83 ± 6.28 |
| 14 | 13 | 36.51 ± 7.34 | 15 | 44.50 ± 7.93 | 15 | 27.89 ± 6.48 |
| 21 | 11 | 48.31 ± 8.89 | 10 | 49.13 ± 4.07 | 12 | 38.94 ± 3.04 |

**Table S5. Summary of Callus IHC data**

| Day after Fracture | Genotype | | | | | |
|---|---|---|---|---|---|---|
| | C57BL/6 (WT) | | *Lyz2*$^{cre/cre}$ | | *Ptgs2*-cKO$^{Lyz2}$ | |
| | N | Mean (+/-) SD | N | Mean (+/-) SD | N | Mean (+/-) SD |
| **Percent COX-2+ Osteoclasts** | | | | | | |
| 10 | 7 | 94.99 ± 5.40 | 9 | 89.08 ± 3.87 | 7 | 40.11 ± 14.45 |
| 14 | 9 | 94.50 ± 5.31 | 9 | 90.93 ± 2.45 | 10 | 52.25 ± 9.42 |
| 21 | 7 | 83.19 ± 10.62 | 9 | 79.06 ± 2.76 | 11 | 32.01 ± 20.32 |
| **CTSK+ Osteoclasts per mm$^2$** | | | | | | |
| 10 | 6 | 21.22 ± 10.80 | 9 | 13.53 ± 4.37 | 6 | 9.66 ± 4.40 |
| 14 | 6 | 52.12 ± 9.12 | 9 | 56.22 ± 6.83 | 8 | 36.91 ± 12.46 |
| 21 | 7 | 53.32 ± 8.35 | 9 | 57.91 ± 10.50 | 10 | 41.67 ± 8.30 |
| **CTSK+ Osteoclasts per Bone Perimeter (mm)** | | | | | | |
| 10 | 6 | 11.88 ± 1.89 | 9 | 9.20 ± 3.01 | 6 | 7.01 ± 2.99 |
| 14 | 6 | 15.75 ± 4.45 | 9 | 18.52 ± 4.55 | 8 | 12.97 ± 5.55 |
| 21 | 7 | 12.41 ± 3.69 | 9 | 12.98 ± 1.91 | 10 | 9.99 ± 3.59 |
| **Percent Collagen X Area** | | | | | | |
| 10 | 6 | 30.17 ± 8.86 | 9 | 32.73 ± 4.41 | 7 | 33.59 ± 9.97 |
| 14 | 6 | 31.68 ± 8.18 | 9 | 34.35 ± 6.75 | 6 | 30.30 ± 10.99 |
| **Chondrocytes** | | | | | | |
| 10 | 9 | 4251.56 ± 1293.57 | 9 | 4326.67 ± 619.70 | 7 | 2943.71 ± 746.04 |
| 14 | 9 | 2857.00 ± 644.21 | 8 | 3161.63 ± 757.54 | 7 | 2367.71 ± 585.30 |
| **MMP-13+ Chondrocytes** | | | | | | |
| 10 | 9 | 1407.67 ± 476.38 | 9 | 1295.11 ± 297.91 | 7 | 635.88 ± 331.39 |
| 14 | 9 | 1560.44 ± 372.25 | 8 | 1414.00 ± 399.15 | 7 | 867.71 ± 346.47 |
| **Percent MMP-13+ Chondrocyte** | | | | | | |
| 10 | 9 | 32.84 ± 6.08 | 9 | 29.75 ± 3.41 | 7 | 27.57 ± 8.31 |
| 14 | 9 | 54.55 ± 2.97 | 8 | 51.15 ± 1.73 | 7 | 29.31 ± 6.33 |
| **Chondrocytes per mm$^2$** | | | | | | |
| 10 | 9 | 474.00 ± 75.61 | 9 | 280.38 ± 158.34 | 7 | 341.88 ± 102.12 |
| 14 | 9 | 342.38 ± 45.94 | 8 | 487.52 ± 102.81 | 7 | 317.05 ± 86.91 |
| **MMP-13+ Chondrocytes per mm$^2$** | | | | | | |
| 10 | 9 | 156.82 ± 41.47 | 9 | 142.29 ± 50.54 | 7 | 81.97 ± 48.68 |
| 14 | 9 | 186.98 ± 29.18 | 8 | 176.96 ± 51.75 | 7 | 99.54 ± 29.87 |
| **MMP-13+ Chondrocytes per Bone Perimeter (mm)** | | | | | | |
| 10 | 9 | 86.92 ± 22.18 | 9 | 77.37 ± 26.03 | 7 | 39.64 ± 18.65 |
| 14 | 9 | 59.24 ± 15.25 | 8 | 56.90 ± 17.09 | 7 | 31.84 ± 12.22 |
| **CD-31+ Callus Lumens** | | | | | | |
| 7 | 6 | 1084.17 ± 342.81 | 6 | 1721.17 ± 1196.63 | 6 | 695.00 ± 260.22 |
| 10 | 6 | 1536.17 ± 299.17 | 9 | 1783.00 ± 1041.71 | 7 | 952.14 ± 310.62 |
| 14 | 6 | 2490.00 ± 779.28 | 9 | 2647.67 ± 1168.01 | 7 | 2267.43 ± 1220.11 |

| Table S5 continued | | | | | | |
|---|---|---|---|---|---|---|
| **CD-31+ Lumens per mm$^2$** | | | | | | |
| 7 | 6 | 138.20 ± 12.26 | 6 | 157.17 ± 10.89 | 6 | 91.54 ± 27.65 |
| 10 | 6 | 190.60 ± 22.60 | 9 | 162.57 ± 39.96 | 7 | 106.66 ± 29.99 |
| 14 | 6 | 264.94 ± 87.41 | 9 | 253.78 ± 70.82 | 7 | 249.80 ± 55.50 |
| **Percent CD-31 Area** | | | | | | |
| 7 | 6 | 12.27 ± 1.75 | 6 | 10.23 ± 2.63 | 6 | 6.69 ± 1.70 |
| 10 | 6 | 13.52 ± 3.36 | 9 | 12.36 ± 3.75 | 7 | 8.21 ± 3.49 |
| 14 | 6 | 21.67 ± 5.85 | 9 | 24.08 ± 4.65 | 7 | 21.87 ± 7.52 |
| **F4/80+ Macrophages** | | | | | | |
| 7 | 6 | 1110.50 ± 200.65 | 6 | 1833.17 ± 1137.45 | 6 | 649.83 ± 132.28 |
| 10 | 6 | 1970.00 ± 858.39 | 6 | 1831.00 ± 1132.82 | 6 | 970.67 ± 197.80 |
| 14 | 6 | 1879.83 ± 596.53 | 6 | 1922.00 ± 978.37 | 6 | 1746.00 ± 903.60 |
| **F4/80+ Macrophages per mm$^2$** | | | | | | |
| 7 | 6 | 163.44 ± 12.83 | 6 | 159.27 ± 5.76 | 6 | 85.29 ± 7.65 |
| 10 | 6 | 189.51 ± 4.93 | 6 | 162.01 ± 23.42 | 6 | 109.98 ± 14.84 |
| 14 | 6 | 203.80 ± 14.96 | 6 | 192.18 ± 22.80 | 6 | 160.97 ± 35.79 |
| **F4/80+ Macrophages per Bone Perimeter mm** | | | | | | |
| 7 | 6 | 66.17 ± 8.05 | 6 | 41.95 ± 9.00 | 6 | 79.75 ± 19.17 |
| 10 | 6 | 77.41 ± 10.34 | 6 | 67.02 ± 19.25 | 6 | 79.67 ± 25.65 |
| 14 | 6 | 96.78 ± 35.78 | 6 | 61.52 ± 18.43 | 6 | 69.55 ± 17.91 |

## Table S6. Summary of µCT data

| Day after Fracture | Genotype | | | | | |
|---|---|---|---|---|---|---|
| | C57BL/6 (WT) | | *Lyz2*$^{cre/cre}$ | | *Ptgs2*-cKO$^{Lyz2}$ | |
| | N | Mean (+/-) SD | N | Mean (+/-) SD | N | Mean (+/-) SD |
| **µCT Callus Tissue Volume (TV)** | | | | | | |
| 14 | 9 | 30.89 ± 7.61 | 7 | 36.81 ± 14.02 | 9 | 32.80 ± 13.68 |
| 21 | 7 | 17.78 ± 3.87 | 8 | 20.89 ± 9.37 | 7 | 18.34 ± 7.44 |
| **µCT Callus Bone Volume (BV)** | | | | | | |
| 14 | 9 | 11.02 ± 2.48 | 7 | 12.84 ± 3.44 | 9 | 6.17 ± 2.21 |
| 21 | 7 | 7.59 ± 1.14 | 8 | 8.52 ± 3.95 | 7 | 6.40 ± 2.72 |
| **µCT BV/TV Percentage** | | | | | | |
| 14 | 9 | 36.24 ± 5.12 | 7 | 36.72 ± 6.22 | 9 | 20.27 ± 7.60 |
| 21 | 7 | 43.43 ± 6.46 | 8 | 42.17 ± 7.49 | 7 | 36.26 ± 8.77 |

**Table S7. Summary of relative mRNA level data**

| Day after Fracture | Genotype | | | | | |
|---|---|---|---|---|---|---|
| | C57BL/6 (WT) | | *Lyz2*$^{cre/cre}$ | | *Ptgs2*-cKO$^{Lyz2}$ | |
| | N | Mean (+/-) SD | N | Mean (+/-) SD | N | Mean (+/-) SD |
| ***Actb*** **(ß-actin) mRNA Relative Expression** | | | | | | |
| 10 | 13 | 1.00 ± 0.00 | 8 | 1.00 ± 0.00 | 8 | 1.00 ± 0.00 |
| 14 | 6 | 1.00 ± 0.00 | 9 | 1.00 ± 0.00 | 9 | 1.00 ± 0.00 |
| ***B2m*** **(ß-2-microglobulin) mRNA Relative Expression** | | | | | | |
| 10 | 6 | 1.00 ± 0.09 | 6 | 1.02 ± 0.29 | 7 | 0.97 ± 0.29 |
| 14 | 6 | 1.10 ± 0.28 | 6 | 1.27 ± 0.50 | 6 | 1.14 ± 0.30 |
| ***Ptgs2*** **(COX-2) mRNA Relative Expression** | | | | | | |
| 10 | 13 | 1.79 ± 1.50 | 8 | 1.26 ± 0.28 | 7 | 0.03 ± 0.04 |
| 14 | 6 | 1.34 ± 0.84 | 8 | 0.54 ± 0.32 | 7 | 0.10 ± 0.08 |
| ***Spp1*** **(Osteopontin) mRNA Relative Expression** | | | | | | |
| 10 | 6 | 1.07 ± 0.46 | 6 | 0.88 ± 0.66 | 6 | 0.30 ± 0.19 |
| 14 | 6 | 1.12 ± 0.53 | 6 | 1.81 ± 0.70 | 6 | 0.31 ± 0.21 |
| ***Sparc*** **(Osteonectin) mRNA Relative Expression** | | | | | | |
| 10 | 6 | 1.27 ± 0.95 | 6 | 1.02 ± 0.61 | 6 | 0.29 ± 0.24 |
| 14 | 6 | 1.10 ± 0.56 | 6 | 2.20 ± 0.94 | 6 | 0.68 ± 0.57 |
| ***Mmp13*** **(MMP-13) mRNA Relative Expression** | | | | | | |
| 10 | 13 | 1.25 ± 0.78 | 8 | 0.75 ± 0.31 | 7 | 0.06 ± 0.04 |
| 14 | 6 | 1.02 ± 0.25 | 8 | 0.70 ± 0.30 | 8 | 0.18 ± 0.14 |
| ***Pecam1*** **(CD31) mRNA Relative Expression** | | | | | | |
| 10 | 13 | 1.74 ± 1.68 | 8 | 2.10 ± 1.71 | 7 | 0.40 ± 0.42 |
| 14 | 6 | 1.05 ± 0.35 | 9 | 0.40 ± 0.31 | 9 | 0.29 ± 0.33 |
| ***Acp5*** **(Trap) mRNA Relative Expression** | | | | | | |
| 10 | 13 | 1.51 ± 1.05 | 8 | 1.35 ± 1.04 | 6 | 0.23 ± 0.25 |
| 14 | 6 | 1.11 ± 0.57 | 9 | 1.58 ± 0.83 | 8 | 0.84 ± 0.75 |
| ***Ctsk*** **(Cathepsin K) mRNA Relative Expression** | | | | | | |
| 10 | 13 | 1.18 ± 0.62 | 8 | 1.35 ± 0.86 | 8 | 0.87 ± 0.72 |
| 14 | 6 | 1.03 ± 0.27 | 8 | 1.46 ± 0.81 | 8 | 0.87 ± 0.67 |
| ***Acan*** **(Aggrecan) mRNA Relative Expression** | | | | | | |
| 10 | 13 | 1.42 ± 1.12 | 9 | 0.94 ± 0.63 | 8 | 0.37 ± 0.39 |
| 14 | 6 | 1.33 ± 0.87 | 8 | 1.34 ± 0.57 | 8 | 1.86 ± 0.57 |
| ***Col10a1*** **(Collagen X) mRNA Relative Expression** | | | | | | |
| 10 | 6 | 1.13 ± 0.61 | 6 | 0.73 ± 0.56 | 7 | 0.31 ± 0.38 |
| 14 | 6 | 1.04 ± 0.33 | 6 | 0.87 ± 0.58 | 6 | 1.33 ± 0.57 |
| ***Postn*** **(Periostin) mRNA Relative Expression** | | | | | | |
| 10 | 6 | 1.16 ± 0.71 | 6 | 1.20 ± 1.06 | 7 | 0.49 ± 0.34 |
| 14 | 6 | 1.23 ± 0.86 | 6 | 0.71 ± 0.25 | 6 | 0.22 ± 0.20 |

| **Table S7 continued** | | | | | | |
|---|---|---|---|---|---|---|
| ***Bglap* (Osteocalcin) mRNA Relative Expression** | | | | | | |
| 10 | 6 | 1.01 ± 0.18 | 6 | 1.30 ± 1.53 | 7 | 0.84 ± 0.53 |
| 14 | 6 | 1.07 ± 0.44 | 6 | 0.94 ± 0.60 | 6 | 0.37 ± 0.48 |
| ***Vegfa* (VEGF-a) mRNA Relative Expression** | | | | | | |
| 10 | 6 | 1.14 ± 0.60 | 6 | 1.42 ± 0.65 | 7 | 0.35 ± 0.46 |
| 14 | 6 | 1.11 ± 0.48 | 6 | 1.39 ± 1.18 | 6 | 1.36 ± 1.02 |
| ***Adgre1* (F4/80) mRNA Relative Expression** | | | | | | |
| 10 | 6 | 1.04 ± 0.31 | 6 | 1.36 ± 1.33 | 7 | 0.14 ± 0.15 |
| 14 | 6 | 1.24 ± 0.96 | 6 | 0.62 ± 0.50 | 6 | 0.58 ± 0.29 |

*ANOVAThreeWay (11/5/2024 15:20:2*

*Descriptive Statistics  Genotype*

| | N | Mean | SD | SEM | Variance | Missing | NonMissing |
|---|---|---|---|---|---|---|---|
| WT | 34 | 416.76471 | 191.49366 | 32.84089 | 36669.82175 | 0 | 34 |
| cKO COX-2 | 34 | 261.52941 | 126.37012 | 21.6723 | 15969.4082 | 0 | 34 |
| Lyz2 | 32 | 370.5 | 221.81378 | 39.21151 | 49201.35484 | 0 | 32 |

*Timepoint*

| | N | Mean | SD | SEM | Variance | Missing | NonMissing |
|---|---|---|---|---|---|---|---|
| 10 | 29 | 158.75862 | 93.09045 | 17.28646 | 8665.83251 | 0 | 29 |
| 14 | 38 | 443.55263 | 167.29513 | 27.13885 | 27987.65932 | 0 | 38 |
| 21 | 33 | 407.84848 | 168.78576 | 29.38183 | 28488.63258 | 0 | 33 |

*Gender*

| | N | Mean | SD | SEM | Variance | Missing | NonMissing |
|---|---|---|---|---|---|---|---|
| Male | 43 | 402.67442 | 223.1973 | 34.03727 | 49817.03433 | 0 | 43 |
| Female | 57 | 308.82456 | 157.27582 | 20.83169 | 24735.68296 | 0 | 57 |

*Genotype*Timepoint*

| | | N | Mean | SD | SEM | Variance | Missing | NonMissing |
|---|---|---|---|---|---|---|---|---|
| WT | 10 | 10 | 226.7 | 110.65065 | 34.99081 | 12243.56667 | 0 | 10 |
| | 14 | 13 | 508.07692 | 158.09779 | 43.84844 | 24994.91026 | 0 | 13 |
| | 21 | 11 | 481.63636 | 168.53562 | 50.8154 | 28404.25455 | 0 | 11 |
| cKO COX-2 | 10 | 8 | 118.375 | 51.93935 | 18.36333 | 2697.69643 | 0 | 8 |
| | 14 | 14 | 331.42857 | 132.90003 | 35.51903 | 17662.41758 | 0 | 14 |
| | 21 | 12 | 275.41667 | 63.12536 | 18.22272 | 3984.81061 | 0 | 12 |
| Lyz2 | 10 | 11 | 126.36364 | 65.43435 | 19.7292 | 4281.65455 | 0 | 11 |
| | 14 | 11 | 510 | 150.76339 | 45.45687 | 22729.6 | 0 | 11 |
| | 21 | 10 | 485.6 | 167.6499 | 53.01555 | 28106.48889 | 0 | 10 |

*Genotype*Gender*

| | | N | Mean | SD | SEM | Variance | Missing | NonMissing |
|---|---|---|---|---|---|---|---|---|
| WT | Male | 17 | 470.41176 | 234.08333 | 56.77355 | 54795.00735 | 0 | 17 |
| | Female | 17 | 363.11765 | 121.32904 | 29.42661 | 14720.73529 | 0 | 17 |
| cKO COX-2 | Male | 17 | 286.82353 | 139.75256 | 33.89498 | 19530.77941 | 0 | 17 |
| | Female | 17 | 236.23529 | 109.75685 | 26.61995 | 12046.56618 | 0 | 17 |
| Lyz2 | Male | 9 | 493.55556 | 255.5881 | 85.19603 | 65325.27778 | 0 | 9 |
| | Female | 23 | 322.34783 | 192.23887 | 40.08458 | 36955.78261 | 0 | 23 |

*Timepoint*Gender*

| | | N | Mean | SD | SEM | Variance | Missing | NonMissing |
|---|---|---|---|---|---|---|---|---|
| 10 | Male | 13 | 202.15385 | 102.52548 | 28.43545 | 10511.47436 | 0 | 13 |
| | Female | 16 | 123.5 | 69.34647 | 17.33662 | 4808.93333 | 0 | 16 |
| 14 | Male | 16 | 536.9375 | 186.62028 | 46.65507 | 34827.12917 | 0 | 16 |
| | Female | 22 | 375.63636 | 113.83511 | 24.26973 | 12958.4329 | 0 | 22 |
| 21 | Male | 14 | 435.42857 | 217.96532 | 58.25368 | 47508.87912 | 0 | 14 |
| | Female | 19 | 387.52632 | 123.72118 | 28.38358 | 15306.92982 | 0 | 19 |

*Genotype*Timepoint*Gender*

| | | | N | Mean | SD | SEM | Variance | Missing | NonMissing |
|---|---|---|---|---|---|---|---|---|---|
| WT | 10 | Male | 6 | 247.83333 | 126.15453 | 51.50237 | 15914.96667 | 0 | 6 |
| | | Female | 4 | 195 | 89.28979 | 44.6449 | 7972.66667 | 0 | 4 |
| | 14 | Male | 7 | 597.71429 | 167.36957 | 63.25975 | 28012.57143 | 0 | 7 |
| | | Female | 6 | 403.5 | 44.72471 | 18.25879 | 2000.3 | 0 | 6 |
| | 21 | Male | 4 | 581.5 | 237.35978 | 118.67989 | 56339.66667 | 0 | 4 |
| | | Female | 7 | 424.57143 | 93.39675 | 35.30065 | 8722.95238 | 0 | 7 |
| cKO COX-2 | 10 | Male | 4 | 130 | 49.17316 | 24.58658 | 2418 | 0 | 4 |
| | | Female | 4 | 106.75 | 59.29798 | 29.64899 | 3516.25 | 0 | 4 |
| | 14 | Male | 6 | 405.33333 | 146.02557 | 59.61469 | 21323.46667 | 0 | 6 |
| | | Female | 8 | 276 | 96.8386 | 34.23762 | 9377.71429 | 0 | 8 |
| | 21 | Male | 7 | 274.85714 | 49.96141 | 18.88364 | 2496.14286 | 0 | 7 |
| | | Female | 5 | 276.2 | 84.92762 | 37.98078 | 7212.7 | 0 | 5 |
| Lyz2 | 10 | Male | 3 | 207 | 54.28628 | 31.3422 | 2947 | 0 | 3 |
| | | Female | 8 | 96.125 | 37.9866 | 13.43029 | 1442.98214 | 0 | 8 |
| | 14 | Male | 3 | 658.33333 | 192.00087 | 110.85175 | 36864.33333 | 0 | 3 |
| | | Female | 8 | 454.375 | 94.72206 | 33.4893 | 8972.26786 | 0 | 8 |
| | 21 | Male | 3 | 615.33333 | 188.00621 | 108.54543 | 35346.33333 | 0 | 3 |
| | | Female | 7 | 430 | 135.48309 | 51.20779 | 18355.66667 | 0 | 7 |

*ANOVA*

*Overall ANOVA*

| | DF | Sum of Squares | Mean Square | F Value | P Value |
|---|---|---|---|---|---|
| Genotype | 2 | 553882.2486 | 276941.1243 | 21.22007 | <0.0001 |
| Timepoint | 2 | 1545719.57225 | 772859.78613 | 59.21886 | <0.0001 |
| Gender | 1 | 305458.87804 | 305458.87804 | 23.40519 | <0.0001 |
| Genotype*Timepoint | 4 | 128930.69276 | 32232.67319 | 2.46977 | 0.051 |
| Genotype*Gender | 2 | 53467.29678 | 26733.64839 | 2.04841 | 0.13549 |
| Timepoint*Gender | 2 | 48450.29565 | 24225.14783 | 1.8562 | 0.16277 |
| Genotype*Timepoint*Gender | 4 | 14973.46858 | 3743.36714 | 0.28683 | 0.88575 |
| Model | 17 | 2623218.46 | 154306.96824 | 11.82347 | <0.0001 |
| Error | 82 | 1070174.3 | 13050.9061 | | |
| Corrected Total | 99 | 3693392.76 | | | |

At the 0.05 level, the population means of **Genotype** are **significantly** different.
At the 0.05 level, the population means of **Timepoint** are **significantly** different.
At the 0.05 level, the population means of **Gender** are **significantly** different.
At the 0.05 level, the population means of **Genotype*Timepoint** are **not significantly** different. At the 0.05 level, the population means of **Genotype*Gender** are **not significantly** different.
At the 0.05 level, the population means of **Timepoint*Gender** are **not significantly** different.
At the 0.05 level, the population means of **Genotype*Timepoint*Gender** are **not significantly** different.

*Means Comparisons  Tukey Test*

*Genotype*

| | MeanDiff | SEM | q Value | Prob | Alpha | Sig | LCL | UCL |
|---|---|---|---|---|---|---|---|---|
| WT cKO COX-2 | 163.49643 | 25.88282 | 8.93329 | <0.0001 | 0.05 | 1 | 101.71402 | 225.27884 |
| WT Lyz2 | -1.84127 | 27.3261 | -0.09529 | 0.9975 | 0.05 | 0 | -67.06877 | 63.38624 |
| cKO COX-2 Lyz2 | -165.3377 | 27.41015 | -8.53052 | <0.0001 | 0.05 | 1 | -230.76583 | -99.90956 |

*Timepoint*

| | MeanDiff | SEM | q Value | Prob | Alpha | Sig | LCL | UCL |
|---|---|---|---|---|---|---|---|---|
| 10 14 | -302.09127 | 26.9019 | -15.88072 | <0.0001 | 0.05 | 1 | -366.30623 | -237.87631 |
| 10 21 | -269.95893 | 27.73104 | -13.76723 | <0.0001 | 0.05 | 1 | -336.15305 | -203.76481 |
| 14 21 | 32.13234 | 25.98517 | 1.74877 | 0.43524 | 0.05 | 0 | -29.89437 | 94.15905 |

*Gender*

| | MeanDiff | SEM | q Value | Prob | Alpha | Sig | LCL | UCL |
|---|---|---|---|---|---|---|---|---|
| Male Female | 117.26481 | 21.9492 | 7.55552 | <0.0001 | 0.05 | 1 | 73.60084 | 160.92879 |

*Genotype  's*

| | Mean | Groups | |
|---|---|---|---|
| WT | 416.76471 | A | |
| Lyz2 | 370.5 | A | |
| cKO COX-2 | 261.52941 | | B |

Means that do not share a letter are significantly different.

*Timepoint  's*

| | Mean | Groups | |
|---|---|---|---|
| 14 | 443.55263 | A | |
| 21 | 407.84848 | A | |
| 10 | 158.75862 | | B |

Means that do not share a letter are significantly different.

*Gender  's Gr*

| | Mean | Groups | |
|---|---|---|---|
| Male | 402.67442 | A | |
| Female | 308.82456 | | B |

Means that do not share a letter are significantly different.

*Interactions ’*

| Genotype | Timepoint | Gender | Mean | Groups | | | | |
|---|---|---|---|---|---|---|---|---|
| Lyz2 | 14 | Male | 658.33333 | A | | | | |
| Lyz2 | 21 | Male | 615.33333 | A | | | | |
| WT | 14 | Male | 597.71429 | A | | | | |
| WT | 21 | Male | 581.5 | A | | | | |
| Lyz2 | 14 | Female | 454.375 | A | B | | | |
| Lyz2 | 21 | Female | 430 | A | B | C | | |
| WT | 21 | Female | 424.57143 | A | B | C | D | |
| cKO COX-2 | 14 | Male | 405.33333 | A | B | C | D | |
| WT | 14 | Female | 403.5 | A | B | C | D | |
| cKO COX-2 | 21 | Female | 276.2 | | B | C | D | E |
| cKO COX-2 | 14 | Female | 276 | | B | C | D | E |
| cKO COX-2 | 21 | Male | 274.85714 | | B | C | D | E |
| WT | 10 | Male | 247.83333 | | | C | D | E |
| Lyz2 | 10 | Male | 207 | | B | C | D | E |
| WT | 10 | Female | 195 | | | | D | E |
| cKO COX-2 | 10 | Male | 130 | | | | | E |
| cKO COX-2 | 10 | Female | 106.75 | | | | | E |
| Lyz2 | 10 | Female | 96.125 | | | | | E |

Means that do not share a letter are significantly different.

Sig equals 1 indicates that the difference of the means is significant at the 0.05 level. Sig equals 0 indicates that the difference of the means is not significant at the 0.05 level.

## S1-B. COX-2 IHC Percent COX-2+ Osteoclasts: (Percent Callus COX-2+ OCs/TRAP+OCs)

*ANOVAThreeWay (11/5/2024 15:33:0*

*Descriptive Statistics Genotype*

| | N | Mean | SD | SEM | Variance | Missing | NonMissing |
|---|---|---|---|---|---|---|---|
| WT | 23 | 91.20783 | 8.85613 | 1.84663 | 78.43105 | 0 | 23 |
| cKO COX-2 | 28 | 41.26429 | 17.57339 | 3.32106 | 308.82403 | 0 | 28 |
| Lyz2 | 27 | 86.35704 | 6.08906 | 1.17184 | 37.07671 | 0 | 27 |

*Timepoint*

| | N | Mean | SD | SEM | Variance | Missing | NonMissing |
|---|---|---|---|---|---|---|---|
| 10 | 23 | 75.97826 | 25.78658 | 5.37687 | 664.94784 | 0 | 23 |
| 14 | 28 | 78.26286 | 20.77848 | 3.92676 | 431.74535 | 0 | 28 |
| 21 | 27 | 60.96148 | 28.07388 | 5.40282 | 788.14287 | 0 | 27 |

*Gender*

| | N | Mean | SD | SEM | Variance | Missing | NonMissing |
|---|---|---|---|---|---|---|---|
| Male | 34 | 65.79618 | 29.57234 | 5.07162 | 874.52349 | 0 | 34 |
| Female | 44 | 76.08523 | 21.90238 | 3.30191 | 479.71418 | 0 | 44 |

*Genotype*Timepoint*

| | | N | Mean | SD | SEM | Variance | Missing | NonMissing |
|---|---|---|---|---|---|---|---|---|
| WT | 10 | 7 | 94.99429 | 5.40333 | 2.04227 | 29.19603 | 0 | 7 |
| | 14 | 9 | 94.49889 | 5.3147 | 1.77157 | 28.24606 | 0 | 9 |
| | 21 | 7 | 83.19 | 10.61837 | 4.01337 | 112.7498 | 0 | 7 |
| cKO COX-2 | 10 | 7 | 40.11429 | 14.45388 | 5.46305 | 208.9146 | 0 | 7 |
| | 14 | 10 | 52.248 | 9.4172 | 2.97798 | 88.68357 | 0 | 10 |
| | 21 | 11 | 32.01091 | 20.32008 | 6.12674 | 412.90571 | 0 | 11 |
| Lyz2 | 10 | 9 | 89.08222 | 3.87181 | 1.2906 | 14.99089 | 0 | 9 |
| | 14 | 9 | 90.93222 | 2.45413 | 0.81804 | 6.02277 | 0 | 9 |
| | 21 | 9 | 79.05667 | 2.76125 | 0.92042 | 7.62448 | 0 | 9 |

*Genotype*Gender*

| | | N | Mean | SD | SEM | Variance | Missing | NonMissing |
|---|---|---|---|---|---|---|---|---|
| WT | Male | 10 | 91.928 | 5.74349 | 1.81625 | 32.98768 | 0 | 10 |
| | Female | 13 | 90.65385 | 10.87588 | 3.01643 | 118.28481 | 0 | 13 |
| cKO COX-2 | Male | 15 | 37.054 | 20.33581 | 5.25068 | 413.54521 | 0 | 15 |
| | Female | 13 | 46.12231 | 12.83197 | 3.55895 | 164.65945 | 0 | 13 |
| Lyz2 | Male | 9 | 84.66444 | 6.59769 | 2.19923 | 43.52958 | 0 | 9 |
| | Female | 18 | 87.20333 | 5.82632 | 1.37328 | 33.946 | 0 | 18 |

*Timepoint*Gender*

| | | N | Mean | SD | SEM | Variance | Missing | NonMissing |
|---|---|---|---|---|---|---|---|---|
| 10 | Male | 10 | 67.012 | 31.30701 | 9.90015 | 980.12895 | 0 | 10 |
| | Female | 13 | 82.87538 | 19.11661 | 5.30199 | 365.44496 | 0 | 13 |
| 14 | Male | 11 | 77.46909 | 17.93921 | 5.40888 | 321.81523 | 0 | 11 |
| | Female | 17 | 78.77647 | 22.95043 | 5.5663 | 526.72231 | 0 | 17 |
| 21 | Male | 13 | 54.98385 | 33.89514 | 9.40082 | 1148.88079 | 0 | 13 |
| | Female | 14 | 66.51214 | 21.13926 | 5.64971 | 446.86843 | 0 | 14 |

*Genotype*Timepoint*Gender*

| | | | N | Mean | SD | SEM | Variance | Missing | NonMissing |
|---|---|---|---|---|---|---|---|---|---|
| WT | 10 | Male | 3 | 93.22667 | 6.54803 | 3.7805 | 42.87663 | 0 | 3 |
| | | Female | 4 | 96.32 | 4.93354 | 2.46677 | 24.3398 | 0 | 4 |
| | 14 | Male | 4 | 90.395 | 5.69901 | 2.84951 | 32.47877 | 0 | 4 |
| | | Female | 5 | 97.782 | 1.34819 | 0.60293 | 1.81762 | 0 | 5 |
| | 21 | Male | 3 | 92.67333 | 6.98474 | 4.03264 | 48.78653 | 0 | 3 |
| | | Female | 4 | 76.0775 | 5.96585 | 2.98293 | 35.59142 | 0 | 4 |
| cKO COX-2 | 10 | Male | 4 | 31.1625 | 4.85291 | 2.42645 | 23.55069 | 0 | 4 |
| | | Female | 3 | 52.05 | 14.74642 | 8.51385 | 217.4569 | 0 | 3 |
| | 14 | Male | 4 | 56.3775 | 10.10341 | 5.0517 | 102.07883 | 0 | 4 |
| | | Female | 6 | 49.495 | 8.69748 | 3.55073 | 75.64611 | 0 | 6 |
| | 21 | Male | 7 | 29.37857 | 23.69223 | 8.95482 | 561.32155 | 0 | 7 |
| | | Female | 4 | 36.6175 | 14.46537 | 7.23268 | 209.24689 | 0 | 4 |
| Lyz2 | 10 | Male | 3 | 88.59667 | 5.68722 | 3.28352 | 32.34443 | 0 | 3 |
| | | Female | 6 | 89.325 | 3.29173 | 1.34384 | 10.83547 | 0 | 6 |
| | 14 | Male | 3 | 88.35667 | 2.35842 | 1.36163 | 5.56213 | 0 | 3 |
| | | Female | 6 | 92.22 | 1.2006 | 0.49014 | 1.44144 | 0 | 6 |
| | 21 | Male | 3 | 77.04 | 2.31758 | 1.33806 | 5.3712 | 0 | 3 |
| | | Female | 6 | 80.065 | 2.52793 | 1.03202 | 6.39043 | 0 | 6 |

*ANOVA*

*Overall ANOVA*

| | DF | Sum of Squares | Mean Square | F Value | P Value |
|---|---|---|---|---|---|
| Genotype | 2 | 35237.4028 | 17618.7014 | 181.21818 | <0.0001 |
| Timepoint | 2 | 2515.78607 | 1257.89303 | 12.93813 | <0.0001 |
| Gender | 1 | 114.7215 | 114.7215 | 1.17997 | 0.28171 |
| Genotype*Timepoint | 4 | 408.9806 | 102.24515 | 1.05165 | 0.38837 |
| Genotype*Gender | 2 | 249.13727 | 124.56864 | 1.28126 | 0.28517 |
| Timepoint*Gender | 2 | 313.58776 | 156.79388 | 1.61271 | 0.20787 |
| Genotype*Timepoint*Gender | 4 | 1144.57085 | 286.14271 | 2.94314 | 0.02743 |
| Model | 17 | 45683.99131 | 2687.29361 | 27.64031 | <0.0001 |
| Error | 60 | 5833.42188 | 97.2237 | | |
| Corrected Total | 77 | 51517.41319 | | | |

At the 0.05 level, the population means of **Genotype** are **significantly** different.
At the 0.05 level, the population means of **Timepoint** are **significantly** different.
At the 0.05 level, the population means of **Gender** are **not significantly** different.
At the 0.05 level, the population means of **Genotype*Timepoint** are **not significantly** different. At the 0.05 level, the population means of **Genotype*Gender** are **not significantly** different.
At the 0.05 level, the population means of **Timepoint*Gender** are **not significantly** different.
At the 0.05 level, the population means of **Genotype*Timepoint*Gender** are **significantly** different.

*Means Comparisons  Tukey Test*

*Genotype*

| | MeanDiff | SEM | q Value | Prob | Alpha | Sig | LCL | UCL |
|---|---|---|---|---|---|---|---|---|
| WT cKO COX-2 | 48.56557 | 2.50041 | 27.46828 | <0.0001 | 0.05 | 1 | 42.55664 | 54.57451 |
| WT Lyz2 | 5.14519 | 2.54453 | 2.85962 | 0.11567 | 0.05 | 0 | -0.96977 | 11.26016 |
| cKO COX-2 Lyz2 | -43.42038 | 2.45147 | -25.04851 | <0.0001 | 0.05 | 1 | -49.31169 | -37.52906 |

*Timepoint*

| | MeanDiff | SEM | q Value | Prob | Alpha | Sig | LCL | UCL |
|---|---|---|---|---|---|---|---|---|
| 10 14 | -3.99089 | 2.51029 | -2.24834 | 0.25787 | 0.05 | 0 | -10.02355 | 2.04177 |
| 10 21 | 9.80482 | 2.5552 | 5.42662 | 8.6956E-4 | 0.05 | 1 | 3.66422 | 15.94542 |
| 14 21 | 13.79571 | 2.43019 | 8.0282 | <0.0001 | 0.05 | 1 | 7.95553 | 19.63589 |

*Gender*

| | MeanDiff | SEM | q Value | Prob | Alpha | Sig | LCL | UCL |
|---|---|---|---|---|---|---|---|---|
| Male Female | -2.52723 | 2.0405 | -1.75155 | 0.22034 | 0.05 | 0 | -6.60885 | 1.55438 |

*Genotype  's*

| | Mean | Groups | |
|---|---|---|---|
| WT | 91.20783 | A | |
| Lyz2 | 86.35704 | A | |
| cKO COX-2 | 41.26429 | | B |

Means that do not share a letter are significantly different.

*Timepoint  's*

| | Mean | Groups | |
|---|---|---|---|
| 14 | 78.26286 | A | |
| 10 | 75.97826 | A | |
| 21 | 60.96148 | | B |

Means that do not share a letter are significantly different.

*Gender  's Gr*

| | Mean | Groups |
|---|---|---|
| Female | 76.08523 | A |
| Male | 65.79618 | A |

Means that do not share a letter are significantly different.

*Interactions ’*

| Genotype | Timepoint | Gender | Mean | Groups | | | | | | |
|---|---|---|---|---|---|---|---|---|---|---|
| WT | 14 | Female | 97.782 | A | B | | | | | |
| WT | 10 | Female | 96.32 | A | | C | | | | |
| WT | 10 | Male | 93.22667 | A | | C | | | | |
| WT | 21 | Male | 92.67333 | A | | C | | | | |
| Lyz2 | 14 | Female | 92.22 | A | | C | | | | |
| WT | 14 | Male | 90.395 | A | | C | | | | |
| Lyz2 | 10 | Female | 89.325 | A | | C | | | | |
| Lyz2 | 10 | Male | 88.59667 | A | | C | | | | |
| Lyz2 | 14 | Male | 88.35667 | A | | C | | | | |
| Lyz2 | 21 | Female | 80.065 | A | | C | | | | |
| Lyz2 | 21 | Male | 77.04 | A | | C | D | | | |
| WT | 21 | Female | 76.0775 | | | C | D | | | |
| cKO COX-2 | 14 | Male | 56.3775 | | | | D | E | | |
| cKO COX-2 | 10 | Female | 52.05 | | | | D | E | F | |
| cKO COX-2 | 14 | Female | 49.495 | | | | | E | F | |
| cKO COX-2 | 21 | Female | 36.6175 | | | | | E | F | G |
| cKO COX-2 | 10 | Male | 31.1625 | | B | | | | F | G |
| cKO COX-2 | 21 | Male | 29.37857 | | | | | | | G |

Means that do not share a letter are significantly different.

Sig equals 1 indicates that the difference of the means is significant at the 0.05 level. Sig equals 0 indicates that the difference of the means is not significant at the 0.05 level.

*ANOVAThreeWay (11/5/2024 18:36:2*

*Descriptive Statistics Genotype*

| | N | Mean | SD | SEM | Variance | Missing | NonMissing |
|---|---|---|---|---|---|---|---|
| WT | 19 | 1.13525 | 0.53113 | 0.12185 | 0.28209 | 0 | 19 |
| Lyz2 | 17 | 1.4032 | 0.81195 | 0.20299 | 0.65927 | 1 | 16 |
| cKO COX-2 | 16 | 0.87149 | 0.67389 | 0.16847 | 0.45413 | 0 | 16 |

*Timepoint*

| | N | Mean | SD | SEM | Variance | Missing | NonMissing |
|---|---|---|---|---|---|---|---|
| 10 | 29 | 1.141 | 0.71721 | 0.13318 | 0.51438 | 0 | 29 |
| 14 | 23 | 1.13072 | 0.67741 | 0.14442 | 0.45888 | 1 | 22 |

*Gender*

| | N | Mean | SD | SEM | Variance | Missing | NonMissing |
|---|---|---|---|---|---|---|---|
| Male | 26 | 1.13197 | 0.76682 | 0.15039 | 0.58801 | 0 | 26 |
| Female | 26 | 1.14134 | 0.6238 | 0.12476 | 0.38913 | 1 | 25 |

*Genotype*Timepoint*

| | | N | Mean | SD | SEM | Variance | Missing | NonMissing |
|---|---|---|---|---|---|---|---|---|
| WT | 10 | 13 | 1.18208 | 0.62013 | 0.17199 | 0.38456 | 0 | 13 |
| | 14 | 6 | 1.03378 | 0.27302 | 0.11146 | 0.07454 | 0 | 6 |
| Lyz2 | 10 | 8 | 1.34641 | 0.86154 | 0.3046 | 0.74224 | 0 | 8 |
| | 14 | 9 | 1.45999 | 0.81431 | 0.2879 | 0.66311 | 1 | 8 |
| cKO COX-2 | 10 | 8 | 0.86884 | 0.72114 | 0.25496 | 0.52004 | 0 | 8 |
| | 14 | 8 | 0.87414 | 0.67311 | 0.23798 | 0.45308 | 0 | 8 |

*Genotype*Gender*

| | | N | Mean | SD | SEM | Variance | Missing | NonMissing |
|---|---|---|---|---|---|---|---|---|
| WT | Male | 9 | 1.08959 | 0.53306 | 0.17769 | 0.28415 | 0 | 9 |
| | Female | 10 | 1.17633 | 0.55467 | 0.1754 | 0.30765 | 0 | 10 |
| Lyz2 | Male | 8 | 1.35698 | 1.02574 | 0.36265 | 1.05213 | 0 | 8 |
| | Female | 9 | 1.44942 | 0.59641 | 0.21086 | 0.3557 | 1 | 8 |
| cKO COX-2 | Male | 9 | 0.97435 | 0.74309 | 0.2477 | 0.55218 | 0 | 9 |
| | Female | 7 | 0.73924 | 0.60234 | 0.22766 | 0.36281 | 0 | 7 |

*Timepoint*Gender*

| | | N | Mean | SD | SEM | Variance | Missing | NonMissing |
|---|---|---|---|---|---|---|---|---|
| 10 | Male | 14 | 1.1086 | 0.79713 | 0.21304 | 0.63542 | 0 | 14 |
| | Female | 15 | 1.17124 | 0.66084 | 0.17063 | 0.43671 | 0 | 15 |
| 14 | Male | 12 | 1.15924 | 0.76416 | 0.22059 | 0.58394 | 0 | 12 |
| | Female | 11 | 1.09648 | 0.5955 | 0.18831 | 0.35462 | 1 | 10 |

*Genotype*Timepoint*Gender*

| | | | N | Mean | SD | SEM | Variance | Missing | NonMissing |
|---|---|---|---|---|---|---|---|---|---|
| WT | 10 | Male | 6 | 1.1276 | 0.65804 | 0.26864 | 0.43302 | 0 | 6 |
| | | Female | 7 | 1.22877 | 0.63463 | 0.23987 | 0.40276 | 0 | 7 |
| | 14 | Male | 3 | 1.01358 | 0.20259 | 0.11697 | 0.04104 | 0 | 3 |
| | | Female | 3 | 1.05398 | 0.37959 | 0.21916 | 0.14409 | 0 | 3 |

| | | | | | | | | | |
|---|---|---|---|---|---|---|---|---|---|
| Lyz2 | 10 | Male | 4 | 1.17865 | 1.25277 | 0.62639 | 1.56944 | 0 | 4 |
| | | Female | 4 | 1.51417 | 0.29565 | 0.14782 | 0.08741 | 0 | 4 |
| | 14 | Male | 4 | 1.53531 | 0.89484 | 0.44742 | 0.80073 | 0 | 4 |
| | | Female | 5 | 1.38468 | 0.85521 | 0.42761 | 0.73139 | 1 | 4 |
| cKO COX-2 | 10 | Male | 4 | 1.01005 | 0.66492 | 0.33246 | 0.44211 | 0 | 4 |
| | | Female | 4 | 0.72763 | 0.84743 | 0.42372 | 0.71814 | 0 | 4 |
| | 14 | Male | 5 | 0.94579 | 0.87777 | 0.39255 | 0.77048 | 0 | 5 |
| | | Female | 3 | 0.75473 | 0.10296 | 0.05944 | 0.0106 | 0 | 3 |

*ANOVA*

*Overall ANOVA*

| | DF | Sum of Squares | Mean Square | F Value | P Value |
|---|---|---|---|---|---|
| Genotype | 2 | 2.3353 | 1.16765 | 2.15794 | 0.12916 |
| Timepoint | 1 | 0.00324 | 0.00324 | 0.00599 | 0.93868 |
| Gender | 1 | 0.00718 | 0.00718 | 0.01327 | 0.90886 |
| Genotype*Timepoint | 2 | 0.13473 | 0.06737 | 0.1245 | 0.88329 |
| Genotype*Gender | 2 | 0.26617 | 0.13309 | 0.24596 | 0.78316 |
| Timepoint*Gender | 1 | 0.06896 | 0.06896 | 0.12744 | 0.72302 |
| Genotype*Timepoint*Gender | 2 | 0.17748 | 0.08874 | 0.164 | 0.84932 |
| Model | 11 | 2.93779 | 0.26707 | 0.49358 | 0.89575 |
| Error | 39 | 21.10272 | 0.5411 | | |
| Corrected Total | 50 | 24.04052 | | | |

At the 0.05 level, the population means of **Genotype** are **not significantly** different.
At the 0.05 level, the population means of **Timepoint** are **not significantly** different.
At the 0.05 level, the population means of **Gender** are **not significantly** different.
At the 0.05 level, the population means of **Genotype*Timepoint** are **not significantly** different.
At the 0.05 level, the population means of **Genotype*Gender** are **not significantly** different.
At the 0.05 level, the population means of **Timepoint*Gender** are **not significantly** different.
At the 0.05 level, the population means of **Genotype*Timepoint*Gender** are **not significantly** different.

*Means Comparisons Tukey Test*

*Genotype*

| | MeanDiff | SEM | q Value | Prob | Alpha | Sig | LCL | UCL |
|---|---|---|---|---|---|---|---|---|
| WT Lyz2 | -0.29722 | 0.22607 | -1.85931 | 0.39567 | 0.05 | 0 | -0.84798 | 0.25355 |
| WT cKO COX-2 | 0.24643 | 0.22797 | 1.52878 | 0.53136 | 0.05 | 0 | -0.30895 | 0.80182 |
| Lyz2 cKO COX-2 | 0.54365 | 0.22931 | 3.3528 | 0.0579 | 0.05 | 0 | -0.01502 | 1.10232 |

*Timepoint*

| | MeanDiff | SEM | q Value | Prob | Alpha | Sig | LCL | UCL |
|---|---|---|---|---|---|---|---|---|
| 10 14 | 0.01647 | 0.18599 | 0.12521 | 0.92991 | 0.05 | 0 | -0.35973 | 0.39266 |

*Gender*

| | MeanDiff | SEM | q Value | Prob | Alpha | Sig | LCL | UCL |
|---|---|---|---|---|---|---|---|---|
| Male Female | 0.0245 | 0.18599 | 0.18633 | 0.89586 | 0.05 | 0 | -0.35169 | 0.4007 |

*Genotype 's*

| | Mean | Groups |
|---|---|---|
| Lyz2 | 1.4032 | A |
| WT | 1.13525 | A |
| cKO COX-2 | 0.87149 | A |

Means that do not share a letter are significantly different.

*Timepoint 's*

| | Mean | Groups |
|---|---|---|
| 10 | 1.141 | A |
| 14 | 1.13072 | A |

Means that do not share a letter are significantly different.

*Gender 's Gr*

| | Mean | Groups |
|---|---|---|
| Female | 1.14134 | A |
| Male | 1.13197 | A |

Means that do not share a letter are significantly different.

*Interactions '*

| Genotype | Timepoint | Gender | Mean | Groups |
|---|---|---|---|---|
| Lyz2 | 14 | Male | 1.53531 | A |
| Lyz2 | 10 | Female | 1.51417 | A |
| Lyz2 | 14 | Female | 1.38468 | A |
| WT | 10 | Female | 1.22877 | A |
| Lyz2 | 10 | Male | 1.17865 | A |
| WT | 10 | Male | 1.1276 | A |
| WT | 14 | Female | 1.05398 | A |
| WT | 14 | Male | 1.01358 | A |
| cKO COX-2 | 10 | Male | 1.01005 | A |
| cKO COX-2 | 14 | Male | 0.94579 | A |
| cKO COX-2 | 14 | Female | 0.75473 | A |
| cKO COX-2 | 10 | Female | 0.72763 | A |

Means that do not share a letter are significantly different.

Sig equals 1 indicates that the difference of the means is significant at the 0.05 level. Sig equals 0 indicates that the difference of the means is not significant at the 0.05 level.

**S1-D. Relative COX-2 (*Ptgs2*) mRNA Levels (RTqPCR)**

*ANOVAThreeWay (11/5/2024 18:20:1*

*Descriptive Statistics Genotype*

| | N | Mean | SD | SEM | Variance | Missing | NonMissing |
|---|---|---|---|---|---|---|---|
| WT | 20 | 1.64571 | 1.3223 | 0.30336 | 1.74847 | 1 | 19 |
| Lyz2 | 18 | 0.89472 | 0.47365 | 0.11841 | 0.22435 | 2 | 16 |
| cKO COX-2 | 17 | 0.06632 | 0.07361 | 0.01967 | 0.00542 | 3 | 14 |

*Timepoint*

| | N | Mean | SD | SEM | Variance | Missing | NonMissing |
|---|---|---|---|---|---|---|---|
| 10 | 32 | 1.19744 | 1.24317 | 0.23494 | 1.54547 | 4 | 28 |
| 14 | 23 | 0.61829 | 0.68312 | 0.14907 | 0.46666 | 2 | 21 |

*Gender*

| | N | Mean | SD | SEM | Variance | Missing | NonMissing |
|---|---|---|---|---|---|---|---|
| Male | 28 | 0.90314 | 1.06946 | 0.21389 | 1.14374 | 3 | 25 |
| Female | 27 | 0.99725 | 1.094 | 0.22331 | 1.19684 | 3 | 24 |

*Genotype*Timepoint*

| | | N | Mean | SD | SEM | Variance | Missing | NonMissing |
|---|---|---|---|---|---|---|---|---|
| WT | 10 | 14 | 1.78857 | 1.50312 | 0.41689 | 2.25937 | 1 | 13 |
| | 14 | 6 | 1.3362 | 0.83904 | 0.34254 | 0.70398 | 0 | 6 |
| Lyz2 | 10 | 10 | 1.25562 | 0.27894 | 0.09862 | 0.07781 | 2 | 8 |
| | 14 | 8 | 0.53382 | 0.32438 | 0.11469 | 0.10523 | 0 | 8 |
| cKO COX-2 | 10 | 8 | 0.03315 | 0.04442 | 0.01679 | 0.00197 | 1 | 7 |
| | 14 | 9 | 0.09949 | 0.08484 | 0.03207 | 0.0072 | 2 | 7 |

*Genotype*Gender*

| | | N | Mean | SD | SEM | Variance | Missing | NonMissing |
|---|---|---|---|---|---|---|---|---|
| WT | Male | 9 | 1.60787 | 1.40647 | 0.46882 | 1.97816 | 0 | 9 |
| | Female | 11 | 1.67977 | 1.31752 | 0.41664 | 1.73586 | 1 | 10 |
| Lyz2 | Male | 10 | 0.94529 | 0.46561 | 0.16462 | 0.21679 | 2 | 8 |
| | Female | 8 | 0.84415 | 0.50804 | 0.17962 | 0.2581 | 0 | 8 |
| cKO COX-2 | Male | 9 | 0.06817 | 0.08062 | 0.0285 | 0.0065 | 1 | 8 |
| | Female | 8 | 0.06386 | 0.07052 | 0.02879 | 0.00497 | 2 | 6 |

*Timepoint*Gender*

| | | N | Mean | SD | SEM | Variance | Missing | NonMissing |
|---|---|---|---|---|---|---|---|---|
| 10 | Male | 16 | 1.13731 | 1.28162 | 0.34253 | 1.64254 | 2 | 14 |
| | Female | 16 | 1.25757 | 1.24879 | 0.33375 | 1.55949 | 2 | 14 |
| 14 | Male | 12 | 0.60511 | 0.6597 | 0.19891 | 0.4352 | 1 | 11 |
| | Female | 11 | 0.6328 | 0.74365 | 0.23516 | 0.55301 | 1 | 10 |

*Genotype*Timepoint*Gender*

| | | | N | Mean | SD | SEM | Variance | Missing | NonMissing |
|---|---|---|---|---|---|---|---|---|---|
| WT | 10 | Male | 6 | 1.77452 | 1.66337 | 0.67907 | 2.7668 | 0 | 6 |
| | | Female | 8 | 1.80061 | 1.48752 | 0.56223 | 2.21271 | 1 | 7 |
| | 14 | Male | 3 | 1.27458 | 0.86354 | 0.49856 | 0.7457 | 0 | 3 |
| | | Female | 3 | 1.39781 | 1.00143 | 0.57818 | 1.00287 | 0 | 3 |

| | | | | | | | | | |
|---|---|---|---|---|---|---|---|---|---|
| Lyz2 | 10 | Male | 6 | 1.2817 | 0.22076 | 0.11038 | 0.04874 | 2 | 4 |
| | | Female | 4 | 1.22954 | 0.36193 | 0.18097 | 0.131 | 0 | 4 |
| | 14 | Male | 4 | 0.60888 | 0.39412 | 0.19706 | 0.15533 | 0 | 4 |
| | | Female | 4 | 0.45876 | 0.27418 | 0.13709 | 0.07517 | 0 | 4 |
| cKO COX-2 | 10 | Male | 4 | 0.0371 | 0.06179 | 0.03089 | 0.00382 | 0 | 4 |
| | | Female | 4 | 0.02787 | 0.01098 | 0.00634 | 1.20627E-4 | 1 | 3 |
| | 14 | Male | 5 | 0.09923 | 0.09368 | 0.04684 | 0.00878 | 1 | 4 |
| | | Female | 4 | 0.09985 | 0.0918 | 0.053 | 0.00843 | 1 | 3 |

*ANOVA*

*Overall ANOVA*

| | DF | Sum of Squares | Mean Square | F Value | P Value |
|---|---|---|---|---|---|
| Genotype | 2 | 16.70967 | 8.35484 | 9.6927 | 4.12238E-4 |
| Timepoint | 1 | 1.55717 | 1.55717 | 1.80652 | 0.18711 |
| Gender | 1 | 0.00121 | 0.00121 | 0.0014 | 0.97036 |
| Genotype*Timepoint | 2 | 1.17199 | 0.58599 | 0.67983 | 0.51292 |
| Genotype*Gender | 2 | 0.06271 | 0.03135 | 0.03637 | 0.96431 |
| Timepoint*Gender | 1 | 2.58945E-5 | 2.58945E-5 | 3.0041E-5 | 0.99566 |
| Genotype*Timepoint*Gender | 2 | 0.01931 | 0.00966 | 0.0112 | 0.98886 |
| Model | 11 | 23.19274 | 2.10843 | 2.44606 | 0.02075 |
| Error | 37 | 31.89296 | 0.86197 | | |
| Corrected Total | 48 | 55.0857 | | | |

At the 0.05 level, the population means of **Genotype** are **significantly** different.
At the 0.05 level, the population means of **Timepoint** are **not significantly** different.
At the 0.05 level, the population means of **Gender** are **not significantly** different.
At the 0.05 level, the population means of **Genotype*Timepoint** are **not significantly** different.
At the 0.05 level, the population means of **Genotype*Gender** are **not significantly** different.
At the 0.05 level, the population means of **Timepoint*Gender** are **not significantly** different.
At the 0.05 level, the population means of **Genotype*Timepoint*Gender** are **not significantly** different.

*Means Comparisons  Tukey Test*

*Genotype*

| | MeanDiff | SEM | q Value | Prob | Alpha | Sig | LCL | UCL |
|---|---|---|---|---|---|---|---|---|
| WT Lyz2 | 0.66716 | 0.28353 | 3.32768 | 0.06084 | 0.05 | 0 | -0.02507 | 1.35939 |
| WT cKO COX-2 | 1.49587 | 0.29525 | 7.16509 | <0.0001 | 0.05 | 1 | 0.77503 | 2.2167 |
| Lyz2 cKO COX-2 | 0.82871 | 0.29688 | 3.94758 | 0.02197 | 0.05 | 1 | 0.10388 | 1.55353 |

*Timepoint*

| | MeanDiff | SEM | q Value | Prob | Alpha | Sig | LCL | UCL |
|---|---|---|---|---|---|---|---|---|
| 10 14 | 0.3687 | 0.23837 | 2.18743 | 0.13044 | 0.05 | 0 | -0.11429 | 0.8517 |

*Gender*

| | MeanDiff | SEM | q Value | Prob | Alpha | Sig | LCL | UCL |
|---|---|---|---|---|---|---|---|---|
| Male Female | 0.01026 | 0.23837 | 0.06089 | 0.96589 | 0.05 | 0 | -0.47273 | 0.49326 |

*Genotype ’s*

| | Mean | Groups | |
|---|---|---|---|
| WT | 1.64571 | A | |
| Lyz2 | 0.89472 | A | |
| cKO COX-2 | 0.06632 | | B |

Means that do not share a letter are significantly different.

*Timepoint ’s*

| | Mean | Groups |
|---|---|---|
| 10 | 1.19744 | A |
| 14 | 0.61829 | A |

Means that do not share a letter are significantly different.

*Gender ’s Gr*

| | Mean | Groups |
|---|---|---|
| Female | 0.99725 | A |
| Male | 0.90314 | A |

Means that do not share a letter are significantly different.

*Interactions ’*

| Genotype | Timepoint | Gender | Mean | Groups | |
|---|---|---|---|---|---|
| WT | 10 | Female | 1.80061 | A | |
| WT | 10 | Male | 1.77452 | A | B |
| WT | 14 | Female | 1.39781 | A | B |
| Lyz2 | 10 | Male | 1.2817 | A | B |
| WT | 14 | Male | 1.27458 | A | B |
| Lyz2 | 10 | Female | 1.22954 | A | B |
| Lyz2 | 14 | Male | 0.60888 | A | B |
| Lyz2 | 14 | Female | 0.45876 | A | B |
| cKO COX-2 | 14 | Female | 0.09985 | A | B |
| cKO COX-2 | 14 | Male | 0.09923 | A | B |
| cKO COX-2 | 10 | Male | 0.0371 | | B |
| cKO COX-2 | 10 | Female | 0.02787 | A | B |

Means that do not share a letter are significantly different.

Sig equals 1 indicates that the difference of the means is significant at the 0.05 level. Sig equals 0 indicates that the difference of the means is not significant at the 0.05 level.

**S2-A. µCT Analysis: Callus Volume ($mm^3$)**

*ANOVAThreeWay (11/5/2024 19:02:2*

*Descriptive Statistics Genotype*

| | N | Mean | SD | SEM | Variance | Missing | NonMissing |
|---|---|---|---|---|---|---|---|
| WT | 16 | 25.15381 | 9.05519 | 2.2638 | 81.99653 | 0 | 16 |
| Lyz2 | 15 | 28.31547 | 13.98936 | 3.61204 | 195.70212 | 0 | 15 |
| cKO COX-2 | 16 | 26.46961 | 13.29577 | 3.32394 | 176.77757 | 0 | 16 |

*Timepoint*

| | N | Mean | SD | SEM | Variance | Missing | NonMissing |
|---|---|---|---|---|---|---|---|
| 14 | 25 | 33.23192 | 11.69128 | 2.33826 | 136.68607 | 0 | 25 |
| 21 | 22 | 19.08676 | 7.16281 | 1.52712 | 51.30578 | 0 | 22 |

*Gender*

| | N | Mean | SD | SEM | Variance | Missing | NonMissing |
|---|---|---|---|---|---|---|---|
| Male | 22 | 28.74099 | 12.86683 | 2.74322 | 165.55533 | 0 | 22 |
| Female | 25 | 24.7362 | 11.24531 | 2.24906 | 126.45704 | 0 | 25 |

*Genotype*Timepoint*

| | | N | Mean | SD | SEM | Variance | Missing | NonMissing |
|---|---|---|---|---|---|---|---|---|
| WT | 14 | 9 | 30.88876 | 7.61297 | 2.53766 | 57.95727 | 0 | 9 |
| | 21 | 7 | 17.7803 | 3.86656 | 1.46142 | 14.95032 | 0 | 7 |
| Lyz2 | 14 | 7 | 36.806 | 14.02153 | 5.29964 | 196.60318 | 0 | 7 |
| | 21 | 8 | 20.88625 | 9.3659 | 3.31135 | 87.72007 | 0 | 8 |
| cKO COX-2 | 14 | 9 | 32.79523 | 13.67772 | 4.55924 | 187.08004 | 0 | 9 |
| | 21 | 7 | 18.33666 | 7.43737 | 2.81106 | 55.31452 | 0 | 7 |

*Genotype*Gender*

| | | N | Mean | SD | SEM | Variance | Missing | NonMissing |
|---|---|---|---|---|---|---|---|---|
| WT | Male | 7 | 28.07023 | 10.71785 | 4.05097 | 114.87235 | 0 | 7 |
| | Female | 9 | 22.88548 | 7.37282 | 2.45761 | 54.35841 | 0 | 9 |
| Lyz2 | Male | 7 | 29.62629 | 14.00609 | 5.2938 | 196.17059 | 0 | 7 |
| | Female | 8 | 27.1685 | 14.83362 | 5.24448 | 220.03631 | 0 | 8 |
| cKO COX-2 | Male | 8 | 28.55326 | 15.1256 | 5.34771 | 228.78381 | 0 | 8 |
| | Female | 8 | 24.38595 | 11.83645 | 4.18482 | 140.10155 | 0 | 8 |

*Timepoint*Gender*

| | | N | Mean | SD | SEM | Variance | Missing | NonMissing |
|---|---|---|---|---|---|---|---|---|
| 14 | Male | 11 | 37.48798 | 11.84627 | 3.57178 | 140.33412 | 0 | 11 |
| | Female | 14 | 29.88787 | 10.81776 | 2.89117 | 117.02403 | 0 | 14 |
| 21 | Male | 11 | 19.99399 | 6.24582 | 1.88319 | 39.01032 | 0 | 11 |
| | Female | 11 | 18.17952 | 8.18053 | 2.46652 | 66.92105 | 0 | 11 |

*Genotype*Timepoint*Gender*

| | | | N | Mean | SD | SEM | Variance | Missing | NonMissing |
|---|---|---|---|---|---|---|---|---|---|
| WT | 14 | Male | 4 | 36.02917 | 5.65338 | 2.82669 | 31.9607 | 0 | 4 |
| | | Female | 5 | 26.77643 | 6.66192 | 2.9793 | 44.38114 | 0 | 5 |
| | 21 | Male | 3 | 17.45831 | 1.03312 | 0.59647 | 1.06733 | 0 | 3 |
| | | Female | 4 | 18.02179 | 5.38588 | 2.69294 | 29.00767 | 0 | 4 |

| | | | | | | | | | |
|---|---|---|---|---|---|---|---|---|---|
| Lyz2 | 14 | Male | 3 | 40.11533 | 12.54267 | 7.24152 | 157.31865 | 0 | 3 |
| | | Female | 4 | 34.324 | 16.40615 | 8.20308 | 269.1618 | 0 | 4 |
| | 21 | Male | 4 | 21.7595 | 9.74304 | 4.87152 | 94.92688 | 0 | 4 |
| | | Female | 4 | 20.013 | 10.37881 | 5.18941 | 107.71978 | 0 | 4 |
| cKO COX-2 | 14 | Male | 4 | 36.97628 | 17.91231 | 8.95616 | 320.85101 | 0 | 4 |
| | | Female | 5 | 29.4504 | 10.10227 | 4.51787 | 102.05584 | 0 | 5 |
| | 21 | Male | 4 | 20.13025 | 4.87704 | 2.43852 | 23.78552 | 0 | 4 |
| | | Female | 3 | 15.9452 | 10.73558 | 6.19819 | 115.25273 | 0 | 3 |

*ANOVA*

*Overall ANOVA*

| | DF | Sum of Squares | Mean Square | F Value | P Value |
|---|---|---|---|---|---|
| Genotype | 2 | 164.98144 | 82.49072 | 0.76679 | 0.47215 |
| Timepoint | 1 | 2591.0983 | 2591.0983 | 24.08554 | <0.0001 |
| Gender | 1 | 247.78832 | 247.78832 | 2.30331 | 0.13808 |
| Genotype*Timepoint | 2 | 13.59055 | 6.79528 | 0.06317 | 0.93889 |
| Genotype*Gender | 2 | 8.84772 | 4.42386 | 0.04112 | 0.95976 |
| Timepoint*Gender | 1 | 93.93786 | 93.93786 | 0.8732 | 0.35647 |
| Genotype*Timepoint*Gender | 2 | 24.30122 | 12.15061 | 0.11295 | 0.89352 |
| Model | 11 | 2934.04825 | 266.73166 | 2.4794 | 0.02035 |
| Error | 35 | 3765.26536 | 107.57901 | | |
| Corrected Total | 46 | 6699.31361 | | | |

At the 0.05 level, the population means of **Genotype** are **not significantly** different.
At the 0.05 level, the population means of **Timepoint** are **significantly** different.
At the 0.05 level, the population means of **Gender** are **not significantly** different.
At the 0.05 level, the population means of **Genotype*Timepoint** are **not significantly** different.
At the 0.05 level, the population means of **Genotype*Gender** are **not significantly** different.
At the 0.05 level, the population means of **Timepoint*Gender** are **not significantly** different.
At the 0.05 level, the population means of **Genotype*Timepoint*Gender** are **not significantly** different.

*Means Comparisons Tukey Test*

*Genotype*

| | MeanDiff | SEM | q Value | Prob | Alpha | Sig | LCL | UCL |
|---|---|---|---|---|---|---|---|---|
| WT Lyz2 | -4.48153 | 3.25548 | -1.94682 | 0.36387 | 0.05 | 0 | -12.44848 | 3.48542 |
| WT cKO COX-2 | -1.05411 | 3.2168 | -0.46342 | 0.94262 | 0.05 | 0 | -8.9264 | 6.81819 |
| Lyz2 cKO COX-2 | 3.42743 | 3.25548 | 1.48891 | 0.5492 | 0.05 | 0 | -4.53952 | 11.39438 |

*Timepoint*

| | MeanDiff | SEM | q Value | Prob | Alpha | Sig | LCL | UCL |
|---|---|---|---|---|---|---|---|---|
| 14 21 | 15.05726 | 2.6476 | 8.04281 | <0.0001 | 0.05 | 1 | 9.68232 | 20.4322 |

*Gender*

| | MeanDiff | SEM | q Value | Prob | Alpha | Sig | LCL | UCL |
|---|---|---|---|---|---|---|---|---|
| Male Female | 4.65634 | 2.6476 | 2.48718 | 0.08737 | 0.05 | 0 | -0.7186 | 10.03127 |

*Genotype 's*

| | Mean | Groups |
|---|---|---|
| Lyz2 | 28.31547 | A |
| cKO COX-2 | 26.46961 | A |
| WT | 25.15381 | A |

Means that do not share a letter are significantly different.

*Timepoint 's*

| | Mean | Groups | |
|---|---|---|---|
| 14 | 33.23192 | A | |
| 21 | 19.08676 | | B |

Means that do not share a letter are significantly different.

*Gender 's Gr*

| | Mean | Groups |
|---|---|---|
| Male | 28.74099 | A |
| Female | 24.7362 | A |

Means that do not share a letter are significantly different.

*Interactions '*

| Genotype | Timepoint | Gender | Mean | Groups |
|---|---|---|---|---|
| Lyz2 | 14 | Male | 40.11533 | A |
| cKO COX-2 | 14 | Male | 36.97628 | A |
| WT | 14 | Male | 36.02917 | A |
| Lyz2 | 14 | Female | 34.324 | A |
| cKO COX-2 | 14 | Female | 29.4504 | A |
| WT | 14 | Female | 26.77643 | A |
| Lyz2 | 21 | Male | 21.7595 | A |
| cKO COX-2 | 21 | Male | 20.13025 | A |
| Lyz2 | 21 | Female | 20.013 | A |
| WT | 21 | Female | 18.02179 | A |
| WT | 21 | Male | 17.45831 | A |
| cKO COX-2 | 21 | Female | 15.9452 | A |

Means that do not share a letter are significantly different.

Sig equals 1 indicates that the difference of the means is significant at the 0.05 level. Sig equals 0 indicates that the difference of the means is not significant at the 0.05 level.

## S2-B. µCT Analysis: Callus Bone Volume ($mm^3$)

*ANOVAThreeWay (11/5/2024 19:04:5*

*Descriptive Statistics Genotype*

| | N | Mean | SD | SEM | Variance | Missing | NonMissing |
|---|---|---|---|---|---|---|---|
| WT | 16 | 9.52134 | 2.62585 | 0.65646 | 6.89511 | 0 | 16 |
| Lyz2 | 15 | 10.53483 | 4.2272 | 1.09146 | 17.86918 | 0 | 15 |
| cKO COX-2 | 16 | 6.27302 | 2.36404 | 0.59101 | 5.58867 | 0 | 16 |

*Timepoint*

| | N | Mean | SD | SEM | Variance | Missing | NonMissing |
|---|---|---|---|---|---|---|---|
| 14 | 25 | 9.78578 | 3.85158 | 0.77032 | 14.83466 | 0 | 25 |
| 21 | 22 | 7.54944 | 2.91412 | 0.62129 | 8.49207 | 0 | 22 |

*Gender*

| | N | Mean | SD | SEM | Variance | Missing | NonMissing |
|---|---|---|---|---|---|---|---|
| Male | 22 | 9.61633 | 3.51255 | 0.74888 | 12.33803 | 0 | 22 |
| Female | 25 | 7.96692 | 3.54704 | 0.70941 | 12.58146 | 0 | 25 |

*Genotype*Timepoint*

| | | N | Mean | SD | SEM | Variance | Missing | NonMissing |
|---|---|---|---|---|---|---|---|---|
| WT | 14 | 9 | 11.02094 | 2.48386 | 0.82795 | 6.16954 | 0 | 9 |
| | 21 | 7 | 7.59329 | 1.14086 | 0.4312 | 1.30155 | 0 | 7 |
| Lyz2 | 14 | 7 | 12.84233 | 3.43962 | 1.30006 | 11.83101 | 0 | 7 |
| | 21 | 8 | 8.51576 | 3.95145 | 1.39705 | 15.61393 | 0 | 8 |
| cKO COX-2 | 14 | 9 | 6.1733 | 2.21109 | 0.73703 | 4.8889 | 0 | 9 |
| | 21 | 7 | 6.40123 | 2.72379 | 1.0295 | 7.41905 | 0 | 7 |

*Genotype*Gender*

| | | N | Mean | SD | SEM | Variance | Missing | NonMissing |
|---|---|---|---|---|---|---|---|---|
| WT | Male | 7 | 10.2469 | 3.32817 | 1.25793 | 11.07673 | 0 | 7 |
| | Female | 9 | 8.95702 | 1.94985 | 0.64995 | 3.8019 | 0 | 9 |
| Lyz2 | Male | 7 | 11.09276 | 4.42136 | 1.67112 | 19.54845 | 0 | 7 |
| | Female | 8 | 10.04664 | 4.28939 | 1.51653 | 18.39888 | 0 | 8 |
| cKO COX-2 | Male | 8 | 7.77271 | 2.07412 | 0.73331 | 4.30199 | 0 | 8 |
| | Female | 8 | 4.77333 | 1.59153 | 0.56269 | 2.53297 | 0 | 8 |

*Timepoint*Gender*

| | | N | Mean | SD | SEM | Variance | Missing | NonMissing |
|---|---|---|---|---|---|---|---|---|
| 14 | Male | 11 | 11.28219 | 3.81417 | 1.15001 | 14.54788 | 0 | 11 |
| | Female | 14 | 8.61003 | 3.57952 | 0.95667 | 12.81294 | 0 | 14 |
| 21 | Male | 11 | 7.95048 | 2.29278 | 0.6913 | 5.25683 | 0 | 11 |
| | Female | 11 | 7.1484 | 3.4961 | 1.05411 | 12.2227 | 0 | 11 |

*Genotype*Timepoint*Gender*

| | | | N | Mean | SD | SEM | Variance | Missing | NonMissing |
|---|---|---|---|---|---|---|---|---|---|
| WT | 14 | Male | 4 | 12.33899 | 2.73175 | 1.36588 | 7.46248 | 0 | 4 |
| | | Female | 5 | 9.96651 | 1.90137 | 0.85032 | 3.6152 | 0 | 5 |
| | 21 | Male | 3 | 7.45745 | 1.26936 | 0.73287 | 1.61128 | 0 | 3 |
| | | Female | 4 | 7.69516 | 1.22337 | 0.61168 | 1.49663 | 0 | 4 |

| | | | | | | | | | | |
|---|---|---|---|---|---|---|---|---|---|---|
| Lyz2 | 14 | Male | 3 | 14.713 | 3.34267 | 1.92989 | 11.17342 | 0 | 3 |
| | | Female | 4 | 11.43933 | 3.17634 | 1.58817 | 10.0891 | 0 | 4 |
| | 21 | Male | 4 | 8.37758 | 2.95164 | 1.47582 | 8.71218 | 0 | 4 |
| | | Female | 4 | 8.65395 | 5.26017 | 2.63009 | 27.66941 | 0 | 4 |
| cKO COX-2 | 14 | Male | 4 | 7.65228 | 1.65939 | 0.82969 | 2.75357 | 0 | 4 |
| | | Female | 5 | 4.99012 | 1.94303 | 0.86895 | 3.77537 | 0 | 5 |
| | 21 | Male | 4 | 7.89315 | 2.69179 | 1.34589 | 7.24573 | 0 | 4 |
| | | Female | 3 | 4.412 | 1.00067 | 0.57774 | 1.00134 | 0 | 3 |

*ANOVA*

*Overall ANOVA*

| | DF | Sum of Squares | Mean Square | F Value | P Value |
|---|---|---|---|---|---|
| Genotype | 2 | 165.44826 | 82.72413 | 11.42501 | 1.51663E-4 |
| Timepoint | 1 | 87.5946 | 87.5946 | 12.09767 | 0.00137 |
| Gender | 1 | 40.35996 | 40.35996 | 5.5741 | 0.02393 |
| Genotype*Timepoint | 2 | 40.53881 | 20.26941 | 2.7994 | 0.07451 |
| Genotype*Gender | 2 | 8.58977 | 4.29488 | 0.59317 | 0.55803 |
| Timepoint*Gender | 1 | 9.05677 | 9.05677 | 1.25083 | 0.27101 |
| Genotype*Timepoint*Gender | 2 | 10.0938 | 5.0469 | 0.69703 | 0.50485 |
| Model | 11 | 339.46852 | 30.86077 | 4.26217 | 4.88435E-4 |
| Error | 35 | 253.42164 | 7.24062 | | |
| Corrected Total | 46 | 592.89016 | | | |

At the 0.05 level, the population means of **Genotype** are **significantly** different.
At the 0.05 level, the population means of **Timepoint** are **significantly** different.
At the 0.05 level, the population means of **Gender** are **significantly** different.
At the 0.05 level, the population means of **Genotype*Timepoint** are **not significantly** different.
At the 0.05 level, the population means of **Genotype*Gender** are **not significantly** different.
At the 0.05 level, the population means of **Timepoint*Gender** are **not significantly** different.
At the 0.05 level, the population means of **Genotype*Timepoint*Gender** are **not significantly** different.

*Means Comparisons Tukey Test*

*Genotype*

| | MeanDiff | SEM | q Value | Prob | Alpha | Sig | LCL | UCL |
|---|---|---|---|---|---|---|---|---|
| WT Lyz2 | -1.43143 | 0.84458 | -2.39689 | 0.22138 | 0.05 | 0 | -3.49832 | 0.63545 |
| WT cKO COX-2 | 3.12764 | 0.83454 | 5.3001 | 0.00182 | 0.05 | 1 | 1.08531 | 5.16997 |
| Lyz2 cKO COX-2 | 4.55908 | 0.84458 | 7.63401 | <0.0001 | 0.05 | 1 | 2.49219 | 6.62596 |

*Timepoint*

| | MeanDiff | SEM | q Value | Prob | Alpha | Sig | LCL | UCL |
|---|---|---|---|---|---|---|---|---|
| 14 21 | 2.76849 | 0.68687 | 5.70008 | 2.85833E-4 | 0.05 | 1 | 1.37406 | 4.16292 |

*Gender*

| | MeanDiff | SEM | q Value | Prob | Alpha | Sig | LCL | UCL |
|---|---|---|---|---|---|---|---|---|
| Male Female | 1.87923 | 0.68687 | 3.86917 | 0.0097 | 0.05 | 1 | 0.4848 | 3.27366 |

*Genotype 's*

| | Mean | Groups | |
|---|---|---|---|
| Lyz2 | 10.53483 | A | |
| WT | 9.52134 | A | |
| cKO COX-2 | 6.27302 | | B |

Means that do not share a letter are significantly different.

*Timepoint 's*

| | Mean | Groups | |
|---|---|---|---|
| 14 | 9.78578 | A | |
| 21 | 7.54944 | | B |

Means that do not share a letter are significantly different.

*Gender 's Gr*

| | Mean | Groups | |
|---|---|---|---|
| Male | 9.61633 | A | |
| Female | 7.96692 | | B |

Means that do not share a letter are significantly different.

*Interactions '*

| Genotype | Timepoint | Gender | Mean | Groups | | |
|---|---|---|---|---|---|---|
| Lyz2 | 14 | Male | 14.713 | A | | |
| WT | 14 | Male | 12.33899 | A | B | |
| Lyz2 | 14 | Female | 11.43933 | A | B | |
| WT | 14 | Female | 9.96651 | A | B | C |
| Lyz2 | 21 | Female | 8.65395 | A | B | C |
| Lyz2 | 21 | Male | 8.37758 | | B | C |
| cKO COX-2 | 21 | Male | 7.89315 | | B | C |
| WT | 21 | Female | 7.69516 | | B | C |
| cKO COX-2 | 14 | Male | 7.65228 | | B | C |
| WT | 21 | Male | 7.45745 | | B | C |
| cKO COX-2 | 14 | Female | 4.99012 | | | C |
| cKO COX-2 | 21 | Female | 4.412 | | | C |

Means that do not share a letter are significantly different.

Sig equals 1 indicates that the difference of the means is significant at the 0.05 level. Sig equals 0 indicates that the difference of the means is not significant at the 0.05 level.

*ANOVAThreeWay (11/5/2024 19:08:1*

*Descriptive Statistics Genotype*

| | N | Mean | SD | SEM | Variance | Missing | NonMissing |
|---|---|---|---|---|---|---|---|
| WT | 16 | 39.38627 | 6.65071 | 1.66268 | 44.23192 | 0 | 16 |
| Lyz2 | 15 | 39.62406 | 7.24718 | 1.87121 | 52.52161 | 0 | 15 |
| cKO COX-2 | 16 | 27.26599 | 11.34036 | 2.83509 | 128.60369 | 0 | 16 |

*Timepoint*

| | N | Mean | SD | SEM | Variance | Missing | NonMissing |
|---|---|---|---|---|---|---|---|
| 14 | 25 | 30.62504 | 10.02383 | 2.00477 | 100.47723 | 0 | 25 |
| 21 | 22 | 40.68959 | 7.90208 | 1.68473 | 62.44294 | 0 | 22 |

*Gender*

| | N | Mean | SD | SEM | Variance | Missing | NonMissing |
|---|---|---|---|---|---|---|---|
| Male | 22 | 35.78567 | 8.51186 | 1.81473 | 72.4517 | 0 | 22 |
| Female | 25 | 34.9405 | 11.8643 | 2.37286 | 140.76162 | 0 | 25 |

*Genotype*Timepoint*

| | | N | Mean | SD | SEM | Variance | Missing | NonMissing |
|---|---|---|---|---|---|---|---|---|
| WT | 14 | 9 | 36.23945 | 5.11659 | 1.70553 | 26.17946 | 0 | 9 |
| | 21 | 7 | 43.43217 | 6.45931 | 2.44139 | 41.72264 | 0 | 7 |
| Lyz2 | 14 | 7 | 36.71598 | 6.21866 | 2.35043 | 38.67172 | 0 | 7 |
| | 21 | 8 | 42.16863 | 7.48594 | 2.64668 | 56.03926 | 0 | 8 |
| cKO COX-2 | 14 | 9 | 20.27324 | 7.60064 | 2.53355 | 57.7698 | 0 | 9 |
| | 21 | 7 | 36.25668 | 8.7653 | 3.31297 | 76.83053 | 0 | 7 |

*Genotype*Gender*

| | | N | Mean | SD | SEM | Variance | Missing | NonMissing |
|---|---|---|---|---|---|---|---|---|
| WT | Male | 7 | 37.78522 | 6.67963 | 2.52466 | 44.61748 | 0 | 7 |
| | Female | 9 | 40.63153 | 6.74421 | 2.24807 | 45.48432 | 0 | 9 |
| Lyz2 | Male | 7 | 39.09813 | 5.38832 | 2.03659 | 29.03399 | 0 | 7 |
| | Female | 8 | 40.08425 | 8.92403 | 3.15512 | 79.63832 | 0 | 8 |
| cKO COX-2 | Male | 8 | 31.13765 | 10.70347 | 3.78425 | 114.56428 | 0 | 8 |
| | Female | 8 | 23.39434 | 11.25846 | 3.98047 | 126.75285 | 0 | 8 |

*Timepoint*Gender*

| | | N | Mean | SD | SEM | Variance | Missing | NonMissing |
|---|---|---|---|---|---|---|---|---|
| 14 | Male | 11 | 31.17424 | 8.25375 | 2.4886 | 68.12438 | 0 | 11 |
| | Female | 14 | 30.19354 | 11.51683 | 3.078 | 132.63732 | 0 | 14 |
| 21 | Male | 11 | 40.39709 | 6.10251 | 1.83998 | 37.24059 | 0 | 11 |
| | Female | 11 | 40.98209 | 9.67995 | 2.91861 | 93.70137 | 0 | 11 |

*Genotype*Timepoint*Gender*

| | | | N | Mean | SD | SEM | Variance | Missing | NonMissing |
|---|---|---|---|---|---|---|---|---|---|
| WT | 14 | Male | 4 | 34.10016 | 3.97195 | 1.98597 | 15.77636 | 0 | 4 |
| | | Female | 5 | 37.95089 | 5.68232 | 2.54121 | 32.28878 | 0 | 5 |
| | 21 | Male | 3 | 42.69864 | 6.84222 | 3.95036 | 46.81593 | 0 | 3 |
| | | Female | 4 | 43.98232 | 7.16192 | 3.58096 | 51.29304 | 0 | 4 |

| | | | | | | | | | |
|---|---|---|---|---|---|---|---|---|---|
| Lyz2 | 14 | Male | 3 | 37.51651 | 4.31648 | 2.49212 | 18.63196 | 0 | 3 |
| | | Female | 4 | 36.11558 | 7.98753 | 3.99377 | 63.80064 | 0 | 4 |
| | 21 | Male | 4 | 40.28435 | 6.4241 | 3.21205 | 41.269 | 0 | 4 |
| | | Female | 4 | 44.05292 | 8.94546 | 4.47273 | 80.02118 | 0 | 4 |
| cKO COX-2 | 14 | Male | 4 | 23.49162 | 8.27935 | 4.13967 | 68.54762 | 0 | 4 |
| | | Female | 5 | 17.69854 | 6.74423 | 3.01611 | 45.48462 | 0 | 5 |
| | 21 | Male | 4 | 38.78368 | 6.54758 | 3.27379 | 42.87083 | 0 | 4 |
| | | Female | 3 | 32.88733 | 11.6784 | 6.74253 | 136.3851 | 0 | 3 |

*ANOVA*

*Overall ANOVA*

| | DF | Sum of Squares | Mean Square | F Value | P Value |
|---|---|---|---|---|---|
| Genotype | 2 | 1325.39891 | 662.69945 | 12.8466 | <0.0001 |
| Timepoint | 1 | 989.0223 | 989.0223 | 19.17245 | 1.03052E-4 |
| Gender | 1 | 5.56635 | 5.56635 | 0.10791 | 0.7445 |
| Genotype*Timepoint | 2 | 209.34088 | 104.67044 | 2.02906 | 0.14664 |
| Genotype*Gender | 2 | 156.87295 | 78.43648 | 1.52051 | 0.23269 |
| Timepoint*Gender | 1 | 1.98281 | 1.98281 | 0.03844 | 0.8457 |
| Genotype*Timepoint*Gender | 2 | 29.38139 | 14.6907 | 0.28478 | 0.7539 |
| Model | 11 | 3102.62807 | 282.0571 | 5.46775 | <0.0001 |
| Error | 35 | 1805.49555 | 51.58559 | | |
| Corrected Total | 46 | 4908.12362 | | | |

At the 0.05 level, the population means of **Genotype** are **significantly** different.
At the 0.05 level, the population means of **Timepoint** are **significantly** different.
At the 0.05 level, the population means of **Gender** are **not significantly** different.
At the 0.05 level, the population means of **Genotype*Timepoint** are **not significantly** different.
At the 0.05 level, the population means of **Genotype*Gender** are **not significantly** different.
At the 0.05 level, the population means of **Timepoint*Gender** are **not significantly** different.
At the 0.05 level, the population means of **Genotype*Timepoint*Gender** are **not significantly** different.

*Means Comparisons Tukey Test*

*Genotype*

| | MeanDiff | SEM | q Value | Prob | Alpha | Sig | LCL | UCL |
|---|---|---|---|---|---|---|---|---|
| WT Lyz2 | 0.19066 | 2.25432 | 0.11961 | 0.99606 | 0.05 | 0 | -5.32621 | 5.70753 |
| WT cKO COX-2 | 11.46771 | 2.22753 | 7.2806 | <0.0001 | 0.05 | 1 | 6.01639 | 16.91903 |
| Lyz2 cKO COX-2 | 11.27705 | 2.25432 | 7.07449 | <0.0001 | 0.05 | 1 | 5.76018 | 16.79392 |

*Timepoint*

| | MeanDiff | SEM | q Value | Prob | Alpha | Sig | LCL | UCL |
|---|---|---|---|---|---|---|---|---|
| 14 21 | -9.30266 | 1.83338 | -7.17577 | <0.0001 | 0.05 | 1 | -13.02464 | -5.58068 |

*Gender*

| | MeanDiff | SEM | q Value | Prob | Alpha | Sig | LCL | UCL |
|---|---|---|---|---|---|---|---|---|
| Male Female | 0.69789 | 1.83338 | 0.53833 | 0.70576 | 0.05 | 0 | -3.02408 | 4.41987 |

*Genotype ’s*

| | Mean | Groups | |
|---|---|---|---|
| Lyz2 | 39.62406 | A | |
| WT | 39.38627 | A | |
| cKO COX-2 | 27.26599 | | B |

Means that do not share a letter are significantly different.

*Timepoint ’s*

| | Mean | Groups | |
|---|---|---|---|
| 21 | 40.68959 | A | |
| 14 | 30.62504 | | B |

Means that do not share a letter are significantly different.

*Gender ’s Gr*

| | Mean | Groups |
|---|---|---|
| Male | 35.78567 | A |
| Female | 34.9405 | A |

Means that do not share a letter are significantly different.

*Interactions ’*

| Genotype | Timepoint | Gender | Mean | Groups | | |
|---|---|---|---|---|---|---|
| Lyz2 | 21 | Female | 44.05292 | A | | |
| WT | 21 | Female | 43.98232 | A | | |
| WT | 21 | Male | 42.69864 | A | | |
| Lyz2 | 21 | Male | 40.28435 | A | | |
| cKO COX-2 | 21 | Male | 38.78368 | A | B | |
| WT | 14 | Female | 37.95089 | A | B | |
| Lyz2 | 14 | Male | 37.51651 | A | B | |
| Lyz2 | 14 | Female | 36.11558 | A | B | |
| WT | 14 | Male | 34.10016 | A | B | |
| cKO COX-2 | 21 | Female | 32.88733 | A | B | C |
| cKO COX-2 | 14 | Male | 23.49162 | | B | C |
| cKO COX-2 | 14 | Female | 17.69854 | | | C |

Means that do not share a letter are significantly different.

Sig equals 1 indicates that the difference of the means is significant at the 0.05 level. Sig equals 0 indicates that the difference of the means is not significant at the 0.05 level.

Figure 3: No Statical Analyses Applied to this Figure; this page is left intentionally Blank.

*ANOVAThreeWay (3/13/2025 14:59:57)*
*Descriptive Statistics Genotype*

**S4-A. Histomorphometry: Callus Percent Cartilage Area: (Cartilage Area/Callus Area)**

| | N | Mean | SD | SEM | Variance | Missing | NonMissing |
|---|---|---|---|---|---|---|---|
| WT | 23 | 21.61435 | 10.95207 | 2.28366 | 119.94775 | 0 | 23 |
| Lyz2 | 26 | 24.24923 | 10.72899 | 2.10413 | 115.11124 | 0 | 26 |
| cKO COX-2 | 21 | 19.16143 | 7.50195 | 1.63706 | 56.27926 | 0 | 21 |

*Timepoint*

| | N | Mean | SD | SEM | Variance | Missing | NonMissing |
|---|---|---|---|---|---|---|---|
| 10 dpf | 29 | 29.51759 | 8.42234 | 1.56399 | 70.93584 | 0 | 29 |
| 14 dpf | 41 | 16.43878 | 7.16459 | 1.11892 | 51.33134 | 0 | 41 |

*Gender*

| | N | Mean | SD | SEM | Variance | Missing | NonMissing |
|---|---|---|---|---|---|---|---|
| Male | 28 | 23.33571 | 10.1694 | 1.92184 | 103.41663 | 0 | 28 |
| Female | 42 | 20.87143 | 9.93967 | 1.53372 | 98.7971 | 0 | 42 |

*Genotype*Timepoint*

| | | N | Mean | SD | SEM | Variance | Missing | NonMissing |
|---|---|---|---|---|---|---|---|---|
| WT | 10 dpf | 10 | 31.375 | 8.09026 | 2.55836 | 65.45225 | 0 | 10 |
| | 14 dpf | 13 | 14.10615 | 5.5093 | 1.528 | 30.35236 | 0 | 13 |
| Lyz2 | 10 dpf | 11 | 33.49091 | 6.11464 | 1.84363 | 37.38877 | 0 | 11 |
| | 14 dpf | 15 | 17.472 | 7.90766 | 2.04175 | 62.53112 | 0 | 15 |
| cKO COX-2 | 10 dpf | 8 | 21.7325 | 6.86841 | 2.42835 | 47.17499 | 0 | 8 |
| | 14 dpf | 13 | 17.57923 | 7.69163 | 2.13327 | 59.16112 | 0 | 13 |

*Genotype*Gender*

| | | N | Mean | SD | SEM | Variance | Missing | NonMissing |
|---|---|---|---|---|---|---|---|---|
| WT | Male | 13 | 23.51154 | 10.24866 | 2.84247 | 105.03511 | 0 | 13 |
| | Female | 10 | 19.148 | 11.8828 | 3.75767 | 141.20102 | 0 | 10 |
| Lyz2 | Male | 6 | 24.66 | 15.35681 | 6.26939 | 235.83164 | 0 | 6 |
| | Female | 20 | 24.126 | 9.45156 | 2.11343 | 89.33193 | 0 | 20 |
| cKO COX-2 | Male | 9 | 22.19889 | 6.42372 | 2.14124 | 41.26421 | 0 | 9 |
| | Female | 12 | 16.88333 | 7.688 | 2.21934 | 59.10541 | 0 | 12 |

*Timepoint*Gender*

| | | N | Mean | SD | SEM | Variance | Missing | NonMissing |
|---|---|---|---|---|---|---|---|---|
| 10 dpf | Male | 13 | 30.17769 | 9.36536 | 2.59748 | 87.71 | 0 | 13 |
| | Female | 16 | 28.98125 | 7.84609 | 1.96152 | 61.56109 | 0 | 16 |
| 14 dpf | Male | 15 | 17.406 | 6.56692 | 1.69557 | 43.1244 | 0 | 15 |
| | Female | 26 | 15.88077 | 7.55615 | 1.48188 | 57.09534 | 0 | 26 |

*Genotype*Timepoint*Gender*

| | | | N | Mean | SD | SEM | Variance | Missing | NonMissing |
|---|---|---|---|---|---|---|---|---|---|
| WT | 10 dpf | Male | 6 | 31.815 | 7.58471 | 3.09645 | 57.52787 | 0 | 6 |
| | | Female | 4 | 30.715 | 9.97542 | 4.98771 | 99.50897 | 0 | 4 |
| | 14 dpf | Male | 7 | 16.39429 | 5.83824 | 2.20665 | 34.08503 | 0 | 7 |
| | | Female | 6 | 11.43667 | 4.0078 | 1.63618 | 16.06247 | 0 | 6 |

| | | | | | | | | | |
|---|---|---|---|---|---|---|---|---|---|
| Lyz2 | 10 dpf | Male | 3 | 38.06333 | 5.3942 | 3.11434 | 29.09743 | 0 | 3 |
| | | Female | 8 | 31.77625 | 5.72527 | 2.02419 | 32.77874 | 0 | 8 |
| | 14 dpf | Male | 3 | 11.25667 | 4.64043 | 2.67916 | 21.53363 | 0 | 3 |
| | | Female | 12 | 19.02583 | 7.90572 | 2.28218 | 62.50041 | 0 | 12 |
| cKO COX-2 | 10 dpf | Male | 4 | 21.8075 | 8.63734 | 4.31867 | 74.60356 | 0 | 4 |
| | | Female | 4 | 21.6575 | 5.95453 | 2.97726 | 35.45643 | 0 | 4 |
| | 14 dpf | Male | 5 | 22.512 | 5.12835 | 2.29347 | 26.30002 | 0 | 5 |
| | | Female | 8 | 14.49625 | 7.62547 | 2.69601 | 58.14774 | 0 | 8 |

*ANOVA*

*Overall ANOVA*

| | DF | Sum of Squares | Mean Square | F Value | P Value |
|---|---|---|---|---|---|
| Genotype | 2 | 227.21172 | 113.60586 | 2.41217 | 0.09856 |
| Timepoint | 1 | 2685.1021 | 2685.1021 | 57.01228 | <0.0001 |
| Gender | 1 | 66.91163 | 66.91163 | 1.42072 | 0.23814 |
| Genotype*Timepoint | 2 | 773.37603 | 386.68802 | 8.21048 | 7.24854E-4 |
| Genotype*Gender | 2 | 60.51009 | 30.25504 | 0.6424 | 0.52973 |
| Timepoint*Gender | 1 | 2.24316 | 2.24316 | 0.04763 | 0.82801 |
| Genotype*Timepoint*Gender | 2 | 320.89231 | 160.44615 | 3.40672 | 0.03991 |
| Model | 11 | 4213.33044 | 383.03004 | 8.13281 | <0.0001 |
| Error | 58 | 2731.62078 | 47.09691 | | |
| Corrected Total | 69 | 6944.95123 | | | |

**At the 0.05 level, the population means of Genotype are not significantly different. At the 0.05 level, the population means of Timepoint are significantly different.**

**At the 0.05 level, the population means of Gender are not significantly different.**

**At the 0.05 level, the population means of Genotype*Timepoint are significantly different. At the 0.05 level, the population means of Genotype*Gender are not significantly different. At the 0.05 level, the population means of Timepoint*Gender are not significantly different.**

**At the 0.05 level, the population means of Genotype*Timepoint*Gender are significantly different.**

*Means Comparisons Tukey Test*

*Genotype*

| | MeanDiff | SEM | q Value | Prob | Alpha | Sig | LCL | UCL |
|---|---|---|---|---|---|---|---|---|
| WT Lyz2 | -2.44028 | 1.97616 | -1.74635 | 0.43773 | 0.05 | 0 | -7.19349 | 2.31293 |
| WT cKO COX-2 | 2.47193 | 1.94506 | 1.79728 | 0.41721 | 0.05 | 0 | -2.20648 | 7.15033 |
| Lyz2 cKO COX-2 | 4.91221 | 2.03622 | 3.41166 | 0.04917 | 0.05 | 1 | 0.01453 | 9.80988 |

*Timepoint*

| | MeanDiff | SEM | q Value | Prob | Alpha | Sig | LCL | UCL |
|---|---|---|---|---|---|---|---|---|
| 10 dpf 14 dpf | 13.45215 | 1.62171 | 11.73098 | <0.0001 | 0.05 | 1 | 10.20594 | 16.69835 |

*Gender*

| | MeanDiff | SEM | q Value | Prob | Alpha | Sig | LCL | UCL |
|---|---|---|---|---|---|---|---|---|
| Male Female | 2.12355 | 1.62171 | 1.85184 | 0.19554 | 0.05 | 0 | -1.12265 | 5.36975 |

*Genotype 's G*

| | Mean | Groups | |
|---|---|---|---|
| Lyz2 | 24.24923 | A | |
| WT | 21.61435 | A | B |
| cKO COX-2 | 19.16143 | | B |

Means that do not share a letter are significantly different.

*Timepoint 's G*

| | Mean | Groups | |
|---|---|---|---|
| 10 dpf | 29.51759 | A | |
| 14 dpf | 16.43878 | | B |

Means that do not share a letter are significantly different.

*Gender 's Gro*

| | Mean | Groups |
|---|---|---|
| Male | 23.33571 | A |
| Female | 20.87143 | A |

Means that do not share a letter are significantly different.

*Interactions 's*

| Genotype | Timepoint | Gender | Mean | Groups | | | |
|---|---|---|---|---|---|---|---|
| Lyz2 | 10 dpf | Male | 38.06333 | A | | | |
| WT | 10 dpf | Male | 31.815 | A | B | | |
| Lyz2 | 10 dpf | Female | 31.77625 | A | B | | |
| WT | 10 dpf | Female | 30.715 | A | B | C | |
| cKO COX-2 | 14 dpf | Male | 22.512 | | B | C | D |
| cKO COX-2 | 10 dpf | Male | 21.8075 | | B | C | D |
| cKO COX-2 | 10 dpf | Female | 21.6575 | | B | C | D |
| Lyz2 | 14 dpf | Female | 19.02583 | | | C | D |
| WT | 14 dpf | Male | 16.39429 | | | | D |
| cKO COX-2 | 14 dpf | Female | 14.49625 | | | | D |
| WT | 14 dpf | Female | 11.43667 | | | | D |
| Lyz2 | 14 dpf | Male | 11.25667 | | | | D |

Means that do not share a letter are significantly different.

Sig equals 1 indicates that the difference of the means is significant at the 0.05 level. Sig equals 0 indicates that the difference of the means is not significant at the 0.05 level.

ANOVAThreeWay (3/13/2025 15:05:13)

**S4-B. Cartilage IHC Callus Percent**
**COL10A1: (COL10A1 area/Callus Area)**

Descriptive Statistics Genotype

| | N | Mean | SD | SEM | Variance | Missing | NonMissing |
|---|---|---|---|---|---|---|---|
| WT | 12 | 30.925 | 8.16529 | 2.35712 | 66.67197 | 0 | 12 |
| Lyz2 | 18 | 33.54 | 5.59352 | 1.3184 | 31.28745 | 0 | 18 |
| cKO COX-2 | 13 | 32.06923 | 10.14797 | 2.81454 | 102.98122 | 0 | 13 |

Timepoint

| | N | Mean | SD | SEM | Variance | Missing | NonMissing |
|---|---|---|---|---|---|---|---|
| 10 dpf | 22 | 32.30409 | 7.51224 | 1.60162 | 56.4338 | 0 | 22 |
| 14 dpf | 21 | 32.43 | 8.26633 | 1.80386 | 68.33223 | 0 | 21 |

Gender

| | N | Mean | SD | SEM | Variance | Missing | NonMissing |
|---|---|---|---|---|---|---|---|
| Male | 18 | 34.05722 | 7.90359 | 1.8629 | 62.46681 | 0 | 18 |
| Female | 25 | 31.1476 | 7.64142 | 1.52828 | 58.39134 | 0 | 25 |

Genotype*Timepoint

| | | N | Mean | SD | SEM | Variance | Missing | NonMissing |
|---|---|---|---|---|---|---|---|---|
| WT | 10 dpf | 6 | 30.17333 | 8.85852 | 3.61648 | 78.47343 | 0 | 6 |
| | 14 dpf | 6 | 31.67667 | 8.17612 | 3.33789 | 66.84891 | 0 | 6 |
| Lyz2 | 10 dpf | 9 | 32.72778 | 4.40765 | 1.46922 | 19.42734 | 0 | 9 |
| | 14 dpf | 9 | 34.35222 | 6.75086 | 2.25029 | 45.57414 | 0 | 9 |
| cKO COX-2 | 10 dpf | 7 | 33.58571 | 9.97471 | 3.77009 | 99.49493 | 0 | 7 |
| | 14 dpf | 6 | 30.3 | 10.99023 | 4.48674 | 120.7852 | 0 | 6 |

Genotype*Gender

| | | N | Mean | SD | SEM | Variance | Missing | NonMissing |
|---|---|---|---|---|---|---|---|---|
| WT | Male | 6 | 30.58 | 8.80429 | 3.59434 | 77.51548 | 0 | 6 |
| | Female | 6 | 31.27 | 8.29923 | 3.38815 | 68.8772 | 0 | 6 |
| Lyz2 | Male | 6 | 34.18333 | 5.5504 | 2.26594 | 30.80695 | 0 | 6 |
| | Female | 12 | 33.21833 | 5.83194 | 1.68354 | 34.01154 | 0 | 12 |
| cKO COX-2 | Male | 6 | 37.40833 | 8.72126 | 3.56044 | 76.06042 | 0 | 6 |
| | Female | 7 | 27.49286 | 9.46779 | 3.57849 | 89.63902 | 0 | 7 |

Timepoint*Gender

| | | N | Mean | SD | SEM | Variance | Missing | NonMissing |
|---|---|---|---|---|---|---|---|---|
| 10 dpf | Male | 9 | 33.65667 | 9.64277 | 3.21426 | 92.98298 | 0 | 9 |
| | Female | 13 | 31.36769 | 5.86928 | 1.62785 | 34.44849 | 0 | 13 |
| 14 dpf | Male | 9 | 34.45778 | 6.27678 | 2.09226 | 39.39799 | 0 | 9 |
| | Female | 12 | 30.90917 | 9.471 | 2.73404 | 89.69986 | 0 | 12 |

Genotype*Timepoint*Gender

| | | | N | Mean | SD | SEM | Variance | Missing | NonMissing |
|---|---|---|---|---|---|---|---|---|---|
| WT | 10 dpf | Male | 3 | 26.78333 | 10.87401 | 6.27811 | 118.24403 | 0 | 3 |
| | | Female | 3 | 33.56333 | 6.59267 | 3.80628 | 43.46323 | 0 | 3 |
| | 14 dpf | Male | 3 | 34.37667 | 5.68336 | 3.28129 | 32.30063 | 0 | 3 |
| | | Female | 3 | 28.97667 | 10.62787 | 6.136 | 112.95163 | 0 | 3 |

| | | | | | | | | | |
|---|---|---|---|---|---|---|---|---|---|
| Lyz2 | 10 dpf | Male | 3 | 36.69333 | 2.66226 | 1.53706 | 7.08763 | 0 | 3 |
| | | Female | 6 | 30.745 | 3.75441 | 1.53273 | 14.09563 | 0 | 6 |
| | 14 dpf | Male | 3 | 31.67333 | 7.14349 | 4.1243 | 51.02943 | 0 | 3 |
| | | Female | 6 | 35.69167 | 6.78587 | 2.77032 | 46.04806 | 0 | 6 |
| cKO COX-2 | 10 dpf | Male | 3 | 37.49333 | 11.82473 | 6.82701 | 139.82413 | 0 | 3 |
| | | Female | 4 | 30.655 | 8.89113 | 4.44556 | 79.05217 | 0 | 4 |
| | 14 dpf | Male | 3 | 37.32333 | 7.09262 | 4.09493 | 50.30523 | 0 | 3 |
| | | Female | 3 | 23.27667 | 10.18215 | 5.87867 | 103.67613 | 0 | 3 |

*ANOVA*

*Overall ANOVA*

| | DF | Sum of Squares | Mean Square | F Value | P Value |
|---|---|---|---|---|---|
| Genotype | 2 | 53.75876 | 26.87938 | 0.44904 | 0.64232 |
| Timepoint | 1 | 5.94369 | 5.94369 | 0.09929 | 0.75479 |
| Gender | 1 | 128.22118 | 128.22118 | 2.14204 | 0.15338 |
| Genotype*Timepoint | 2 | 46.53338 | 23.26669 | 0.38869 | 0.6812 |
| Genotype*Gender | 2 | 232.58385 | 116.29193 | 1.94275 | 0.16036 |
| Timepoint*Gender | 1 | 24.77241 | 24.77241 | 0.41384 | 0.52476 |
| Genotype*Timepoint*Gender | 2 | 242.61612 | 121.30806 | 2.02655 | 0.14888 |
| Model | 11 | 696.28553 | 63.29868 | 1.05746 | 0.42428 |
| Error | 31 | 1855.63913 | 59.85933 | | |
| Corrected Total | 42 | 2551.92466 | | | |

**At the 0.05 level, the population means of Genotype are not significantly different. At the 0.05 level, the population means of Timepoint are not significantly different. At the 0.05 level, the population means of Gender are not significantly different.**
**At the 0.05 level, the population means of Genotype*Timepoint are not significantly different. At the 0.05 level, the population means of Genotype*Gender are not significantly different.**
**At the 0.05 level, the population means of Timepoint*Gender are not significantly different.**
**At the 0.05 level, the population means of Genotype*Timepoint*Gender are not significantly different.**

*Means Comparisons Tukey Test*

*Genotype*

| | MeanDiff | SEM | q Value | Prob | Alpha | Sig | LCL | UCL |
|---|---|---|---|---|---|---|---|---|
| WT Lyz2 | -2.77583 | 2.50865 | -1.56483 | 0.51734 | 0.05 | 0 | -8.95003 | 3.39836 |
| WT cKO COX-2 | -1.26208 | 2.63963 | -0.67618 | 0.88209 | 0.05 | 0 | -7.75863 | 5.23446 |
| Lyz2 cKO COX-2 | 1.51375 | 2.46345 | 0.86901 | 0.81333 | 0.05 | 0 | -4.54919 | 7.57669 |

*Timepoint*

| | MeanDiff | SEM | q Value | Prob | Alpha | Sig | LCL | UCL |
|---|---|---|---|---|---|---|---|---|
| 10 dpf 14 dpf | 0.76917 | 2.07255 | 0.52484 | 0.71307 | 0.05 | 0 | -3.45784 | 4.99617 |

*Gender*

| | MeanDiff | SEM | q Value | Prob | Alpha | Sig | LCL | UCL |
|---|---|---|---|---|---|---|---|---|
| Male Female | 3.5725 | 2.07255 | 2.43771 | 0.09472 | 0.05 | 0 | -0.6545 | 7.7995 |

*Genotype ’s G*

| | Mean | Groups |
|---|---|---|
| Lyz2 | 33.54 | A |
| cKO COX-2 | 32.06923 | A |
| WT | 30.925 | A |

Means that do not share a letter are significantly different.

*Timepoint ’s G*

| | Mean | Groups |
|---|---|---|
| 14 dpf | 32.43 | A |
| 10 dpf | 32.30409 | A |

Means that do not share a letter are significantly different.

*Gender ’s Gro*

| | Mean | Groups |
|---|---|---|
| Male | 34.05722 | A |
| Female | 31.1476 | A |

Means that do not share a letter are significantly different.

*Interactions ’s*

| Genotype | Timepoint | Gender | Mean | Groups |
|---|---|---|---|---|
| cKO COX-2 | 10 dpf | Male | 37.49333 | A |
| cKO COX-2 | 14 dpf | Male | 37.32333 | A |
| Lyz2 | 10 dpf | Male | 36.69333 | A |
| Lyz2 | 14 dpf | Female | 35.69167 | A |
| WT | 14 dpf | Male | 34.37667 | A |
| WT | 10 dpf | Female | 33.56333 | A |
| Lyz2 | 14 dpf | Male | 31.67333 | A |
| Lyz2 | 10 dpf | Female | 30.745 | A |
| cKO COX-2 | 10 dpf | Female | 30.655 | A |
| WT | 14 dpf | Female | 28.97667 | A |
| WT | 10 dpf | Male | 26.78333 | A |
| cKO COX-2 | 14 dpf | Female | 23.27667 | A |

Means that do not share a letter are significantly different.

Sig equals 1 indicates that the difference of the means is significant at the 0.05 level. Sig equals 0 indicates that the difference of the means is not significant at the 0.05 level.

*ANOVAThreeWay (11/5/2024 16:52:2*

*Descriptive Statistics Genotype*

| | N | Mean | SD | SEM | Variance | Missing | NonMissing |
|---|---|---|---|---|---|---|---|
| WT | 18 | 43.69333 | 12.09664 | 2.85121 | 146.32879 | 0 | 18 |
| cKO COX-2 | 14 | 28.43714 | 10.67725 | 2.85361 | 114.00361 | 0 | 14 |
| Lyz2 | 17 | 39.81706 | 11.32927 | 2.74775 | 128.35243 | 0 | 17 |

*Timepoint*

| | N | Mean | SD | SEM | Variance | Missing | NonMissing |
|---|---|---|---|---|---|---|---|
| 10 | 25 | 28.3408 | 7.65612 | 1.53122 | 58.61624 | 0 | 25 |
| 14 | 24 | 48.04042 | 8.8081 | 1.79795 | 77.5826 | 0 | 24 |

*Gender*

| | N | Mean | SD | SEM | Variance | Missing | NonMissing |
|---|---|---|---|---|---|---|---|
| Male | 21 | 36.63714 | 13.5446 | 2.95567 | 183.45612 | 0 | 21 |
| Female | 28 | 39.00393 | 12.48226 | 2.35893 | 155.80683 | 0 | 28 |

*Genotype*Timepoint*

| | | N | Mean | SD | SEM | Variance | Missing | NonMissing |
|---|---|---|---|---|---|---|---|---|
| WT | 10 | 9 | 32.83889 | 6.08347 | 2.02782 | 37.00859 | 0 | 9 |
| | 14 | 9 | 54.54778 | 2.97446 | 0.99149 | 8.84742 | 0 | 9 |
| cKO COX-2 | 10 | 7 | 20.75 | 8.31043 | 3.14105 | 69.0632 | 0 | 7 |
| | 14 | 7 | 36.12429 | 6.32953 | 2.39234 | 40.0629 | 0 | 7 |
| Lyz2 | 10 | 9 | 29.74667 | 3.41169 | 1.13723 | 11.6396 | 0 | 9 |
| | 14 | 8 | 51.14625 | 1.73215 | 0.61241 | 3.00034 | 0 | 8 |

*Genotype*Gender*

| | | N | Mean | SD | SEM | Variance | Missing | NonMissing |
|---|---|---|---|---|---|---|---|---|
| WT | Male | 8 | 41.49375 | 12.2086 | 4.31639 | 149.04988 | 0 | 8 |
| | Female | 10 | 45.453 | 12.3584 | 3.90807 | 152.73007 | 0 | 10 |
| cKO COX-2 | Male | 7 | 27.56857 | 13.02387 | 4.92256 | 169.62125 | 0 | 7 |
| | Female | 7 | 29.30571 | 8.69634 | 3.28691 | 75.62626 | 0 | 7 |
| Lyz2 | Male | 6 | 40.74167 | 12.18685 | 4.97526 | 148.51942 | 0 | 6 |
| | Female | 11 | 39.31273 | 11.4154 | 3.44187 | 130.31146 | 0 | 11 |

*Timepoint*Gender*

| | | N | Mean | SD | SEM | Variance | Missing | NonMissing |
|---|---|---|---|---|---|---|---|---|
| 10 | Male | 12 | 27.9375 | 8.50944 | 2.45646 | 72.41051 | 0 | 12 |
| | Female | 13 | 28.71308 | 7.10939 | 1.97179 | 50.54339 | 0 | 13 |
| 14 | Male | 9 | 48.23667 | 9.70477 | 3.23492 | 94.1826 | 0 | 9 |
| | Female | 15 | 47.92267 | 8.57898 | 2.21508 | 73.59888 | 0 | 15 |

*Genotype*Timepoint*Gender*

| | | | N | Mean | SD | SEM | Variance | Missing | NonMissing |
|---|---|---|---|---|---|---|---|---|---|
| WT | 10 | Male | 5 | 33.004 | 4.06312 | 1.81708 | 16.50893 | 0 | 5 |
| | | Female | 4 | 32.6325 | 8.75074 | 4.37537 | 76.57543 | 0 | 4 |
| | 14 | Male | 3 | 55.64333 | 2.855 | 1.64834 | 8.15103 | 0 | 3 |
| | | Female | 6 | 54 | 3.13293 | 1.27901 | 9.81524 | 0 | 6 |

| | | | | | | | | | |
|---|---|---|---|---|---|---|---|---|---|
| cKO COX-2 | 10 | Male | 4 | 19.945 | 9.36313 | 4.68156 | 87.66817 | 0 | 4 |
| | | Female | 3 | 21.82333 | 8.52427 | 4.92149 | 72.66323 | 0 | 3 |
| | 14 | Male | 3 | 37.73333 | 10.30244 | 5.94812 | 106.14023 | 0 | 3 |
| | | Female | 4 | 34.9175 | 2.19882 | 1.09941 | 4.83482 | 0 | 4 |
| Lyz2 | 10 | Male | 3 | 30.15 | 5.87037 | 3.38926 | 34.4613 | 0 | 3 |
| | | Female | 6 | 29.545 | 2.1662 | 0.88435 | 4.69243 | 0 | 6 |
| | 14 | Male | 3 | 51.33333 | 0.53575 | 0.30932 | 0.28703 | 0 | 3 |
| | | Female | 5 | 51.034 | 2.25057 | 1.00649 | 5.06508 | 0 | 5 |

*ANOVA*

*Overall ANOVA*

| | DF | Sum of Squares | Mean Square | F Value | P Value |
|---|---|---|---|---|---|
| Genotype | 2 | 1877.58486 | 938.79243 | 31.30788 | <0.0001 |
| Timepoint | 1 | 4387.53824 | 4387.53824 | 146.32044 | <0.0001 |
| Gender | 1 | 4.72187 | 4.72187 | 0.15747 | 0.69378 |
| Genotype*Timepoint | 2 | 94.70864 | 47.35432 | 1.57922 | 0.21971 |
| Genotype*Gender | 2 | 0.80081 | 0.40041 | 0.01335 | 0.98674 |
| Timepoint*Gender | 1 | 10.17123 | 10.17123 | 0.3392 | 0.56382 |
| Genotype*Timepoint*Gender | 2 | 11.75961 | 5.87981 | 0.19609 | 0.82279 |
| Model | 11 | 6833.65169 | 621.24106 | 20.71783 | <0.0001 |
| Error | 37 | 1109.47531 | 29.98582 | | |
| Corrected Total | 48 | 7943.12699 | | | |

At the 0.05 level, the population means of **Genotype** are **significantly** different.
At the 0.05 level, the population means of **Timepoint** are **significantly** different.
At the 0.05 level, the population means of **Gender** are **not significantly** different.
At the 0.05 level, the population means of **Genotype*Timepoint** are **not significantly** different.
At the 0.05 level, the population means of **Genotype*Gender** are **not significantly** different.
At the 0.05 level, the population means of **Timepoint*Gender** are **not significantly** different.
At the 0.05 level, the population means of **Genotype*Timepoint*Gender** are **not significantly** different.

*Means Comparisons Tukey Test*

*Genotype*

| | MeanDiff | SEM | q Value | Prob | Alpha | Sig | LCL | UCL |
|---|---|---|---|---|---|---|---|---|
| WT cKO COX-2 | 15.21517 | 1.73072 | 12.43268 | <0.0001 | 0.05 | 1 | 10.9897 | 19.44064 |
| WT Lyz2 | 3.30438 | 1.67532 | 2.78937 | 0.1333 | 0.05 | 0 | -0.78585 | 7.3946 |
| cKO COX-2 Lyz2 | -11.91079 | 1.76446 | -9.54649 | <0.0001 | 0.05 | 1 | -16.21864 | -7.60295 |

*Timepoint*

| | MeanDiff | SEM | q Value | Prob | Alpha | Sig | LCL | UCL |
|---|---|---|---|---|---|---|---|---|
| 10 14 | -19.59361 | 1.40755 | -19.68633 | <0.0001 | 0.05 | 1 | -22.44559 | -16.74163 |

*Gender*

| | MeanDiff | SEM | q Value | Prob | Alpha | Sig | LCL | UCL |
|---|---|---|---|---|---|---|---|---|
| Male Female | 0.64278 | 1.40755 | 0.64582 | 0.65058 | 0.05 | 0 | -2.2092 | 3.49476 |

*Genotype 's*

| | Mean | Groups | |
|---|---|---|---|
| WT | 43.69333 | A | |
| Lyz2 | 39.81706 | A | |
| cKO COX-2 | 28.43714 | | B |

Means that do not share a letter are significantly different.

*Timepoint 's*

| | Mean | Groups | |
|---|---|---|---|
| 14 | 48.04042 | A | |
| 10 | 28.3408 | | B |

Means that do not share a letter are significantly different.

*Gender 's Gr*

| | Mean | Groups |
|---|---|---|
| Female | 39.00393 | A |
| Male | 36.63714 | A |

Means that do not share a letter are significantly different.

*Interactions '*

| Genotype | Timepoint | Gender | Mean | Groups | | | |
|---|---|---|---|---|---|---|---|
| WT | 14 | Male | 55.64333 | A | | | |
| WT | 14 | Female | 54 | A | | | |
| Lyz2 | 14 | Male | 51.33333 | A | | | |
| Lyz2 | 14 | Female | 51.034 | A | | | |
| cKO COX-2 | 14 | Male | 37.73333 | | B | | |
| cKO COX-2 | 14 | Female | 34.9175 | | B | | |
| WT | 10 | Male | 33.004 | | B | C | |
| WT | 10 | Female | 32.6325 | | B | C | |
| Lyz2 | 10 | Male | 30.15 | | B | C | D |
| Lyz2 | 10 | Female | 29.545 | | B | C | D |
| cKO COX-2 | 10 | Female | 21.82333 | | | C | D |
| cKO COX-2 | 10 | Male | 19.945 | | | | D |

Means that do not share a letter are significantly different.

Sig equals 1 indicates that the difference of the means is significant at the 0.05 level. Sig equals 0 indicates that the difference of the means is not significant at the 0.05 level.

*ANOVAThreeWay (11/5/2024 18:38:5*

*Descriptive Statistics Genotype*

| | N | Mean | SD | SEM | Variance | Missing | NonMissing |
|---|---|---|---|---|---|---|---|
| WT | 20 | 1.39399 | 1.02532 | 0.23523 | 1.05129 | 1 | 19 |
| Lyz2 | 20 | 1.12632 | 0.61705 | 0.14966 | 0.38076 | 3 | 17 |
| cKO COX-2 | 17 | 1.11685 | 0.90087 | 0.22522 | 0.81157 | 1 | 16 |

*Timepoint*

| | N | Mean | SD | SEM | Variance | Missing | NonMissing |
|---|---|---|---|---|---|---|---|
| 10 | 32 | 0.99829 | 0.92561 | 0.16899 | 0.85676 | 2 | 30 |
| 14 | 25 | 1.52519 | 0.68012 | 0.145 | 0.46256 | 3 | 22 |

*Gender*

| | N | Mean | SD | SEM | Variance | Missing | NonMissing |
|---|---|---|---|---|---|---|---|
| Male | 30 | 1.04178 | 0.65028 | 0.12753 | 0.42287 | 4 | 26 |
| Female | 27 | 1.40065 | 1.01661 | 0.19937 | 1.0335 | 1 | 26 |

*Genotype*Timepoint*

| | | N | Mean | SD | SEM | Variance | Missing | NonMissing |
|---|---|---|---|---|---|---|---|---|
| WT | 10 | 13 | 1.42382 | 1.12142 | 0.31103 | 1.25759 | 0 | 13 |
| | 14 | 7 | 1.32938 | 0.87126 | 0.35569 | 0.75909 | 1 | 6 |
| Lyz2 | 10 | 11 | 0.9387 | 0.63007 | 0.21002 | 0.39699 | 2 | 9 |
| | 14 | 9 | 1.3374 | 0.56605 | 0.20013 | 0.32041 | 1 | 8 |
| cKO COX-2 | 10 | 8 | 0.37384 | 0.39161 | 0.13846 | 0.15336 | 0 | 8 |
| | 14 | 9 | 1.85985 | 0.56912 | 0.20121 | 0.3239 | 1 | 8 |

*Genotype*Gender*

| | | N | Mean | SD | SEM | Variance | Missing | NonMissing |
|---|---|---|---|---|---|---|---|---|
| WT | Male | 10 | 1.04416 | 0.33016 | 0.11005 | 0.109 | 1 | 9 |
| | Female | 10 | 1.70884 | 1.3316 | 0.42109 | 1.77315 | 0 | 10 |
| Lyz2 | Male | 11 | 1.02515 | 0.64776 | 0.21592 | 0.4196 | 2 | 9 |
| | Female | 9 | 1.24015 | 0.60232 | 0.21295 | 0.36279 | 1 | 8 |
| cKO COX-2 | Male | 9 | 1.05779 | 0.95156 | 0.33643 | 0.90547 | 1 | 8 |
| | Female | 8 | 1.1759 | 0.90865 | 0.32126 | 0.82565 | 0 | 8 |

*Timepoint*Gender*

| | | N | Mean | SD | SEM | Variance | Missing | NonMissing |
|---|---|---|---|---|---|---|---|---|
| 10 | Male | 16 | 0.70495 | 0.51869 | 0.13393 | 0.26904 | 1 | 15 |
| | Female | 16 | 1.29164 | 1.14946 | 0.29679 | 1.32127 | 1 | 15 |
| 14 | Male | 14 | 1.50109 | 0.5275 | 0.15905 | 0.27826 | 3 | 11 |
| | Female | 11 | 1.5493 | 0.83177 | 0.25079 | 0.69184 | 0 | 11 |

*Genotype*Timepoint*Gender*

| | | | N | Mean | SD | SEM | Variance | Missing | NonMissing |
|---|---|---|---|---|---|---|---|---|---|
| WT | 10 | Male | 6 | 1.02559 | 0.25095 | 0.10245 | 0.06298 | 0 | 6 |
| | | Female | 7 | 1.76516 | 1.47247 | 0.55654 | 2.16818 | 0 | 7 |
| | 14 | Male | 4 | 1.08131 | 0.52485 | 0.30302 | 0.27547 | 1 | 3 |
| | | Female | 3 | 1.57744 | 1.19902 | 0.69226 | 1.43766 | 0 | 3 |

| | | | | | | | | | |
|---|---|---|---|---|---|---|---|---|---|
| Lyz2 | 10 | Male | 6 | 0.68753 | 0.65673 | 0.2937 | 0.43129 | 1 | 5 |
| | | Female | 5 | 1.25267 | 0.497 | 0.2485 | 0.24701 | 1 | 4 |
| | 14 | Male | 5 | 1.44718 | 0.34118 | 0.17059 | 0.11641 | 1 | 4 |
| | | Female | 4 | 1.22762 | 0.77401 | 0.387 | 0.59909 | 0 | 4 |
| cKO COX-2 | 10 | Male | 4 | 0.24575 | 0.29706 | 0.14853 | 0.08824 | 0 | 4 |
| | | Female | 4 | 0.50194 | 0.47523 | 0.23761 | 0.22584 | 0 | 4 |
| | 14 | Male | 5 | 1.86983 | 0.51584 | 0.25792 | 0.26609 | 1 | 4 |
| | | Female | 4 | 1.84986 | 0.69957 | 0.34979 | 0.4894 | 0 | 4 |

*ANOVA*

*Overall ANOVA*

| | DF | Sum of Squares | Mean Square | F Value | P Value |
|---|---|---|---|---|---|
| Genotype | 2 | 0.57261 | 0.28631 | 0.46608 | 0.63082 |
| Timepoint | 1 | 4.36671 | 4.36671 | 7.10855 | 0.01102 |
| Gender | 1 | 1.12887 | 1.12887 | 1.83769 | 0.18283 |
| Genotype*Timepoint | 2 | 5.18328 | 2.59164 | 4.21892 | 0.02175 |
| Genotype*Gender | 2 | 0.61432 | 0.30716 | 0.50002 | 0.61026 |
| Timepoint*Gender | 1 | 0.58137 | 0.58137 | 0.9464 | 0.33648 |
| Genotype*Timepoint*Gender | 2 | 0.1915 | 0.09575 | 0.15587 | 0.85619 |
| Model | 11 | 13.51178 | 1.22834 | 1.99962 | 0.05462 |
| Error | 40 | 24.5716 | 0.61429 | | |
| Corrected Total | 51 | 38.08339 | | | |

At the 0.05 level, the population means of **Genotype** are **not significantly** different.
At the 0.05 level, the population means of **Timepoint** are **significantly** different.
At the 0.05 level, the population means of **Gender** are **not significantly** different.
At the 0.05 level, the population means of **Genotype*Timepoint** are **significantly** different.
At the 0.05 level, the population means of **Genotype*Gender** are **not significantly** different.
At the 0.05 level, the population means of **Timepoint*Gender** are **not significantly** different.
At the 0.05 level, the population means of **Genotype*Timepoint*Gender** are **not significantly** different.

*Means Comparisons Tukey Test*

*Genotype*

| | MeanDiff | SEM | q Value | Prob | Alpha | Sig | LCL | UCL |
|---|---|---|---|---|---|---|---|---|
| WT Lyz2 | 0.20862 | 0.23851 | 1.23702 | 0.65918 | 0.05 | 0 | -0.37188 | 0.78913 |
| WT cKO COX-2 | 0.24553 | 0.24158 | 1.4373 | 0.57104 | 0.05 | 0 | -0.34246 | 0.83352 |
| Lyz2 cKO COX-2 | 0.0369 | 0.23998 | 0.21748 | 0.98705 | 0.05 | 0 | -0.54718 | 0.62098 |

*Timepoint*

| | MeanDiff | SEM | q Value | Prob | Alpha | Sig | LCL | UCL |
|---|---|---|---|---|---|---|---|---|
| 10 14 | -0.59577 | 0.19598 | -4.2991 | 0.00416 | 0.05 | 1 | -0.99186 | -0.19967 |

*Gender*

| | MeanDiff | SEM | q Value | Prob | Alpha | Sig | LCL | UCL |
|---|---|---|---|---|---|---|---|---|
| Male Female | -0.30292 | 0.19598 | -2.18586 | 0.13007 | 0.05 | 0 | -0.69901 | 0.09318 |

*Genotype 's*

| | Mean | Groups |
|---|---|---|
| WT | 1.39399 | A |
| Lyz2 | 1.12632 | A |
| cKO COX-2 | 1.11685 | A |

Means that do not share a letter are significantly different.

*Timepoint 's*

| | Mean | Groups | |
|---|---|---|---|
| 14 | 1.52519 | A | |
| 10 | 0.99829 | | B |

Means that do not share a letter are significantly different.

*Gender 's Gr*

| | Mean | Groups |
|---|---|---|
| Female | 1.40065 | A |
| Male | 1.04178 | A |

Means that do not share a letter are significantly different.

*Interactions '*

| Genotype | Timepoint | Gender | Mean | Groups | |
|---|---|---|---|---|---|
| cKO COX-2 | 14 | Male | 1.86983 | A | B |
| cKO COX-2 | 14 | Female | 1.84986 | A | B |
| WT | 10 | Female | 1.76516 | A | |
| WT | 14 | Female | 1.57744 | A | B |
| Lyz2 | 14 | Male | 1.44718 | A | B |
| Lyz2 | 10 | Female | 1.25267 | A | B |
| Lyz2 | 14 | Female | 1.22762 | A | B |
| WT | 14 | Male | 1.08131 | A | B |
| WT | 10 | Male | 1.02559 | A | B |
| Lyz2 | 10 | Male | 0.68753 | A | B |
| cKO COX-2 | 10 | Female | 0.50194 | A | B |
| cKO COX-2 | 10 | Male | 0.24575 | | B |

Means that do not share a letter are significantly different.

Sig equals 1 indicates that the difference of the means is significant at the 0.05 level. Sig equals 0 indicates that the difference of the means is not significant at the 0.05 level.

*ANOVAThreeWay (11/5/2024 18:41:1*

*Descriptive Statistics  Genotype*

| | N | Mean | SD | SEM | Variance | Missing | NonMissing |
|---|---|---|---|---|---|---|---|
| WT | 16 | 1.08848 | 0.4712 | 0.13602 | 0.22203 | 4 | 12 |
| Lyz2 | 17 | 0.80087 | 0.54853 | 0.15835 | 0.30088 | 5 | 12 |
| cKO COX-2 | 14 | 0.78562 | 0.70152 | 0.19457 | 0.49213 | 1 | 13 |

*Timepoint*

| | N | Mean | SD | SEM | Variance | Missing | NonMissing |
|---|---|---|---|---|---|---|---|
| 10 | 25 | 0.7041 | 0.60238 | 0.13819 | 0.36286 | 6 | 19 |
| 14 | 22 | 1.08375 | 0.5147 | 0.12131 | 0.26491 | 4 | 18 |

*Gender*

| | N | Mean | SD | SEM | Variance | Missing | NonMissing |
|---|---|---|---|---|---|---|---|
| Male | 25 | 0.89935 | 0.59458 | 0.13641 | 0.35353 | 6 | 19 |
| Female | 22 | 0.87765 | 0.59405 | 0.14002 | 0.3529 | 4 | 18 |

*Genotype*Timepoint*

| | | N | Mean | SD | SEM | Variance | Missing | NonMissing |
|---|---|---|---|---|---|---|---|---|
| WT | 10 | 9 | 1.13273 | 0.61189 | 0.2498 | 0.37441 | 3 | 6 |
| | 14 | 7 | 1.04422 | 0.33068 | 0.135 | 0.10935 | 1 | 6 |
| Lyz2 | 10 | 8 | 0.73313 | 0.56482 | 0.23059 | 0.31903 | 2 | 6 |
| | 14 | 9 | 0.86861 | 0.57611 | 0.2352 | 0.3319 | 3 | 6 |
| cKO COX-2 | 10 | 8 | 0.3118 | 0.38287 | 0.14471 | 0.14659 | 1 | 7 |
| | 14 | 6 | 1.3384 | 0.5694 | 0.23246 | 0.32422 | 0 | 6 |

*Genotype*Gender*

| | | N | Mean | SD | SEM | Variance | Missing | NonMissing |
|---|---|---|---|---|---|---|---|---|
| WT | Male | 8 | 1.08142 | 0.47729 | 0.19485 | 0.22781 | 2 | 6 |
| | Female | 8 | 1.09554 | 0.51042 | 0.20838 | 0.26053 | 2 | 6 |
| Lyz2 | Male | 10 | 0.78749 | 0.2838 | 0.11586 | 0.08054 | 4 | 6 |
| | Female | 7 | 0.81425 | 0.76221 | 0.31117 | 0.58097 | 1 | 6 |
| cKO COX-2 | Male | 7 | 0.83916 | 0.86818 | 0.32814 | 0.75374 | 0 | 7 |
| | Female | 7 | 0.72315 | 0.51761 | 0.21131 | 0.26792 | 1 | 6 |

*Timepoint*Gender*

| | | N | Mean | SD | SEM | Variance | Missing | NonMissing |
|---|---|---|---|---|---|---|---|---|
| 10 | Male | 13 | 0.61681 | 0.52527 | 0.16611 | 0.27591 | 3 | 10 |
| | Female | 12 | 0.80108 | 0.69708 | 0.23236 | 0.48593 | 3 | 9 |
| 14 | Male | 12 | 1.21328 | 0.52382 | 0.17461 | 0.27439 | 3 | 9 |
| | Female | 10 | 0.95421 | 0.5008 | 0.16693 | 0.2508 | 1 | 9 |

*Genotype*Timepoint*Gender*

| | | | N | Mean | SD | SEM | Variance | Missing | NonMissing |
|---|---|---|---|---|---|---|---|---|---|
| WT | 10 | Male | 4 | 1.10208 | 0.62304 | 0.35972 | 0.38818 | 1 | 3 |
| | | Female | 5 | 1.16339 | 0.73826 | 0.42623 | 0.54503 | 2 | 3 |
| | 14 | Male | 4 | 1.06076 | 0.42433 | 0.24498 | 0.18005 | 1 | 3 |
| | | Female | 3 | 1.02769 | 0.30413 | 0.17559 | 0.09249 | 0 | 3 |

| | | | | | | | | | |
|---|---|---|---|---|---|---|---|---|---|
| Lyz2 | 10 | Male | 5 | 0.74201 | 0.20709 | 0.11956 | 0.04288 | 2 | 3 |
| | | Female | 3 | 0.72426 | 0.86859 | 0.50148 | 0.75444 | 0 | 3 |
| | 14 | Male | 5 | 0.83298 | 0.39022 | 0.22529 | 0.15227 | 2 | 3 |
| | | Female | 4 | 0.90425 | 0.82078 | 0.47388 | 0.67368 | 1 | 3 |
| cKO COX-2 | 10 | Male | 4 | 0.15896 | 0.09843 | 0.04921 | 0.00969 | 0 | 4 |
| | | Female | 4 | 0.51559 | 0.56234 | 0.32467 | 0.31623 | 1 | 3 |
| | 14 | Male | 3 | 1.74609 | 0.29591 | 0.17085 | 0.08757 | 0 | 3 |
| | | Female | 3 | 0.93071 | 0.47365 | 0.27346 | 0.22434 | 0 | 3 |

*ANOVA*

*Overall ANOVA*

| | DF | Sum of Squares | Mean Square | F Value | P Value |
|---|---|---|---|---|---|
| Genotype | 2 | 0.59158 | 0.29579 | 1.065 | 0.35985 |
| Timepoint | 1 | 1.12188 | 1.12188 | 4.03937 | 0.05536 |
| Gender | 1 | 0.03629 | 0.03629 | 0.13066 | 0.72079 |
| Genotype*Timepoint | 2 | 2.06992 | 1.03496 | 3.72642 | 0.03833 |
| Genotype*Gender | 2 | 0.13048 | 0.06524 | 0.2349 | 0.79237 |
| Timepoint*Gender | 1 | 0.35393 | 0.35393 | 1.27432 | 0.26967 |
| Genotype*Timepoint*Gender | 2 | 0.72601 | 0.363 | 1.30701 | 0.28849 |
| Model | 11 | 5.4238 | 0.49307 | 1.77533 | 0.11367 |
| Error | 25 | 6.94341 | 0.27774 | | |
| Corrected Total | 36 | 12.3672 | | | |

At the 0.05 level, the population means of **Genotype** are **not significantly** different.
At the 0.05 level, the population means of **Timepoint** are **not significantly** different.
At the 0.05 level, the population means of **Gender** are **not significantly** different.
At the 0.05 level, the population means of **Genotype*Timepoint** are **significantly** different.
At the 0.05 level, the population means of **Genotype*Gender** are **not significantly** different.
At the 0.05 level, the population means of **Timepoint*Gender** are **not significantly** different.
At the 0.05 level, the population means of **Genotype*Timepoint*Gender** are **not significantly** different.

*Means Comparisons  Tukey Test*

*Genotype*

| | MeanDiff | SEM | q Value | Prob | Alpha | Sig | LCL | UCL |
|---|---|---|---|---|---|---|---|---|
| WT Lyz2 | 0.2876 | 0.17685 | 2.29986 | 0.25352 | 0.05 | 0 | -0.1529 | 0.72811 |
| WT cKO COX-2 | 0.25064 | 0.17407 | 2.03633 | 0.33643 | 0.05 | 0 | -0.18293 | 0.68421 |
| Lyz2 cKO COX-2 | -0.03697 | 0.17407 | -0.30033 | 0.97547 | 0.05 | 0 | -0.47053 | 0.3966 |

*Timepoint*

| | MeanDiff | SEM | q Value | Prob | Alpha | Sig | LCL | UCL |
|---|---|---|---|---|---|---|---|---|
| 10 14 | -0.34937 | 0.14289 | -3.45782 | 0.02187 | 0.05 | 1 | -0.64365 | -0.05508 |

*Gender*

| | MeanDiff | SEM | q Value | Prob | Alpha | Sig | LCL | UCL |
|---|---|---|---|---|---|---|---|---|
| Male Female | 0.06283 | 0.14289 | 0.62189 | 0.6639 | 0.05 | 0 | -0.23145 | 0.35712 |

*Genotype 's*

| | Mean | Groups |
|---|---|---|
| WT | 1.08848 | A |
| Lyz2 | 0.80087 | A |
| cKO COX-2 | 0.78562 | A |

Means that do not share a letter are significantly different.

*Timepoint 's*

| | Mean | Groups | |
|---|---|---|---|
| 14 | 1.08375 | A | |
| 10 | 0.7041 | | B |

Means that do not share a letter are significantly different.

*Gender 's Gr*

| | Mean | Groups |
|---|---|---|
| Male | 0.89935 | A |
| Female | 0.87765 | A |

Means that do not share a letter are significantly different.

*Interactions '*

| Genotype | Timepoint | Gender | Mean | Groups | |
|---|---|---|---|---|---|
| cKO COX-2 | 14 | Male | 1.74609 | A | |
| WT | 10 | Female | 1.16339 | A | B |
| WT | 10 | Male | 1.10208 | A | B |
| WT | 14 | Male | 1.06076 | A | B |
| WT | 14 | Female | 1.02769 | A | B |
| cKO COX-2 | 14 | Female | 0.93071 | A | B |
| Lyz2 | 14 | Female | 0.90425 | A | B |
| Lyz2 | 14 | Male | 0.83298 | A | B |
| Lyz2 | 10 | Male | 0.74201 | A | B |
| Lyz2 | 10 | Female | 0.72426 | A | B |
| cKO COX-2 | 10 | Female | 0.51559 | A | B |
| cKO COX-2 | 10 | Male | 0.15896 | | B |

Means that do not share a letter are significantly different.

Sig equals 1 indicates that the difference of the means is significant at the 0.05 level. Sig equals 0 indicates that the difference of the means is not significant at the 0.05 level.

## S4-F. Relative *Mmp13* mRNA Levels (RTqPCR)

*ANOVAThreeWay (11/5/2024 18:28:3*

*Descriptive Statistics Genotype*

| | N | Mean | SD | SEM | Variance | Missing | NonMissing |
|---|---|---|---|---|---|---|---|
| WT | 20 | 1.17895 | 0.65709 | 0.15075 | 0.43176 | 1 | 19 |
| Lyz2 | 20 | 0.7238 | 0.2961 | 0.07403 | 0.08768 | 4 | 16 |
| cKO COX-2 | 16 | 0.12208 | 0.12204 | 0.03151 | 0.01489 | 1 | 15 |

*Timepoint*

| | N | Mean | SD | SEM | Variance | Missing | NonMissing |
|---|---|---|---|---|---|---|---|
| 10 | 33 | 0.80833 | 0.73141 | 0.13822 | 0.53496 | 5 | 28 |
| 14 | 23 | 0.59903 | 0.41838 | 0.0892 | 0.17505 | 1 | 22 |

*Gender*

| | N | Mean | SD | SEM | Variance | Missing | NonMissing |
|---|---|---|---|---|---|---|---|
| Male | 29 | 0.66102 | 0.53116 | 0.10417 | 0.28213 | 3 | 26 |
| Female | 27 | 0.77606 | 0.7053 | 0.14397 | 0.49744 | 3 | 24 |

*Genotype*Timepoint*

| | | N | Mean | SD | SEM | Variance | Missing | NonMissing |
|---|---|---|---|---|---|---|---|---|
| WT | 10 | 14 | 1.25019 | 0.77669 | 0.21541 | 0.60324 | 1 | 13 |
| | 14 | 6 | 1.02458 | 0.2545 | 0.1039 | 0.06477 | 0 | 6 |
| Lyz2 | 10 | 11 | 0.74766 | 0.31124 | 0.11004 | 0.09687 | 3 | 8 |
| | 14 | 9 | 0.69994 | 0.29951 | 0.10589 | 0.08971 | 1 | 8 |
| cKO COX-2 | 10 | 8 | 0.05707 | 0.03795 | 0.01434 | 0.00144 | 1 | 7 |
| | 14 | 8 | 0.17896 | 0.14364 | 0.05079 | 0.02063 | 0 | 8 |

*Genotype*Gender*

| | | N | Mean | SD | SEM | Variance | Missing | NonMissing |
|---|---|---|---|---|---|---|---|---|
| WT | Male | 9 | 1.11231 | 0.51355 | 0.17118 | 0.26373 | 0 | 9 |
| | Female | 11 | 1.23892 | 0.78782 | 0.24913 | 0.62066 | 1 | 10 |
| Lyz2 | Male | 11 | 0.71569 | 0.31208 | 0.11034 | 0.09739 | 3 | 8 |
| | Female | 9 | 0.73192 | 0.30056 | 0.10626 | 0.09034 | 1 | 8 |
| cKO COX-2 | Male | 9 | 0.16115 | 0.13963 | 0.04654 | 0.0195 | 0 | 9 |
| | Female | 7 | 0.06347 | 0.06034 | 0.02463 | 0.00364 | 1 | 6 |

*Timepoint*Gender*

| | | N | Mean | SD | SEM | Variance | Missing | NonMissing |
|---|---|---|---|---|---|---|---|---|
| 10 | Male | 16 | 0.71151 | 0.63568 | 0.16989 | 0.40409 | 2 | 14 |
| | Female | 17 | 0.90515 | 0.82873 | 0.22149 | 0.68679 | 3 | 14 |
| 14 | Male | 13 | 0.60212 | 0.39574 | 0.11424 | 0.15661 | 1 | 12 |
| | Female | 10 | 0.59532 | 0.46583 | 0.14731 | 0.217 | 0 | 10 |

*Genotype*Timepoint*Gender*

| | | | N | Mean | SD | SEM | Variance | Missing | NonMissing |
|---|---|---|---|---|---|---|---|---|---|
| WT | 10 | Male | 6 | 1.1657 | 0.63644 | 0.25983 | 0.40506 | 0 | 6 |
| | | Female | 8 | 1.32261 | 0.92503 | 0.34963 | 0.85568 | 1 | 7 |
| | 14 | Male | 3 | 1.00552 | 0.12888 | 0.07441 | 0.01661 | 0 | 3 |
| | | Female | 3 | 1.04364 | 0.37977 | 0.21926 | 0.14422 | 0 | 3 |

| | | | | | | | | | |
|---|---|---|---|---|---|---|---|---|---|
| Lyz2 | 10 | Male | 6 | 0.68829 | 0.28863 | 0.14431 | 0.08331 | 2 | 4 |
| | | Female | 5 | 0.80703 | 0.36513 | 0.18257 | 0.13332 | 1 | 4 |
| | 14 | Male | 5 | 0.74309 | 0.37675 | 0.18837 | 0.14194 | 1 | 4 |
| | | Female | 4 | 0.6568 | 0.24984 | 0.12492 | 0.06242 | 0 | 4 |
| cKO COX-2 | 10 | Male | 4 | 0.05345 | 0.04434 | 0.02217 | 0.00197 | 0 | 4 |
| | | Female | 4 | 0.06191 | 0.03618 | 0.02089 | 0.00131 | 1 | 3 |
| | 14 | Male | 5 | 0.24732 | 0.12899 | 0.05769 | 0.01664 | 0 | 5 |
| | | Female | 3 | 0.06502 | 0.08823 | 0.05094 | 0.00778 | 0 | 3 |

*ANOVA*

*Overall ANOVA*

| | DF | Sum of Squares | Mean Square | F Value | P Value |
|---|---|---|---|---|---|
| Genotype | 2 | 8.12003 | 4.06001 | 17.4632 | <0.0001 |
| Timepoint | 1 | 0.03685 | 0.03685 | 0.15852 | 0.69275 |
| Gender | 1 | 9.30998E-4 | 9.30998E-4 | 0.004 | 0.94987 |
| Genotype*Timepoint | 2 | 0.19481 | 0.09741 | 0.41897 | 0.66073 |
| Genotype*Gender | 2 | 0.06507 | 0.03253 | 0.13994 | 0.86986 |
| Timepoint*Gender | 1 | 0.08562 | 0.08562 | 0.36826 | 0.54756 |
| Genotype*Timepoint*Gender | 2 | 0.0043 | 0.00215 | 0.00925 | 0.9908 |
| Model | 11 | 9.82496 | 0.89318 | 3.8418 | 9.2308E-4 |
| Error | 38 | 8.83461 | 0.23249 | | |
| Corrected Total | 49 | 18.65956 | | | |

At the 0.05 level, the population means of **Genotype** are **significantly** different.
At the 0.05 level, the population means of **Timepoint** are **not significantly** different.
At the 0.05 level, the population means of **Gender** are **not significantly** different.
At the 0.05 level, the population means of **Genotype*Timepoint** are **not significantly** different.
At the 0.05 level, the population means of **Genotype*Gender** are **not significantly** different.
At the 0.05 level, the population means of **Timepoint*Gender** are **not significantly** different.
At the 0.05 level, the population means of **Genotype*Timepoint*Gender** are **not significantly** different.

*Means Comparisons  Tukey Test*

*Genotype*

| | MeanDiff | SEM | q Value | Prob | Alpha | Sig | LCL | UCL |
|---|---|---|---|---|---|---|---|---|
| WT Lyz2 | 0.41056 | 0.14773 | 3.93037 | 0.02242 | 0.05 | 1 | 0.05029 | 0.77084 |
| WT cKO COX-2 | 1.02744 | 0.15203 | 9.55772 | <0.0001 | 0.05 | 1 | 0.65668 | 1.3982 |
| Lyz2 cKO COX-2 | 0.61688 | 0.15289 | 5.70611 | 7.29504E-4 | 0.05 | 1 | 0.24402 | 0.98974 |

*Timepoint*

| | MeanDiff | SEM | q Value | Prob | Alpha | Sig | LCL | UCL |
|---|---|---|---|---|---|---|---|---|
| 10 14 | 0.05627 | 0.12321 | 0.64588 | 0.65049 | 0.05 | 0 | -0.19315 | 0.30569 |

*Gender*

| | MeanDiff | SEM | q Value | Prob | Alpha | Sig | LCL | UCL |
|---|---|---|---|---|---|---|---|---|
| Male Female | -0.00894 | 0.12321 | -0.10266 | 0.94251 | 0.05 | 0 | -0.25836 | 0.24048 |

*Genotype 's*

| | Mean | Groups | | |
|---|---|---|---|---|
| WT | 1.17895 | A | | |
| Lyz2 | 0.7238 | | B | |
| cKO COX-2 | 0.12208 | | | C |

Means that do not share a letter are significantly different.

*Timepoint 's*

| | Mean | Groups |
|---|---|---|
| 10 | 0.80833 | A |
| 14 | 0.59903 | A |

Means that do not share a letter are significantly different.

*Gender 's Gr*

| | Mean | Groups |
|---|---|---|
| Female | 0.77606 | A |
| Male | 0.66102 | A |

Means that do not share a letter are significantly different.

*Interactions '*

| Genotype | Timepoint | Gender | Mean | Groups | |
|---|---|---|---|---|---|
| WT | 10 | Female | 1.32261 | A | |
| WT | 10 | Male | 1.1657 | A | |
| WT | 14 | Female | 1.04364 | A | B |
| WT | 14 | Male | 1.00552 | A | B |
| Lyz2 | 10 | Female | 0.80703 | A | B |
| Lyz2 | 14 | Male | 0.74309 | A | B |
| Lyz2 | 10 | Male | 0.68829 | A | B |
| Lyz2 | 14 | Female | 0.6568 | A | B |
| cKO COX-2 | 14 | Male | 0.24732 | | B |
| cKO COX-2 | 14 | Female | 0.06502 | | B |
| cKO COX-2 | 10 | Female | 0.06191 | | B |
| cKO COX-2 | 10 | Male | 0.05345 | | B |

Means that do not share a letter are significantly different.

Sig equals 1 indicates that the difference of the means is significant at the 0.05 level. Sig equals 0 indicates that the difference of the means is not significant at the 0.05 level.

*ANOVAThreeWay (11/5/2024 15:54:4*

*Descriptive Statistics Genotype*

| | N | Mean | SD | SEM | Variance | Missing | NonMissing |
|---|---|---|---|---|---|---|---|
| WT | 18 | 197.91288 | 72.8342 | 17.16719 | 5304.82035 | 0 | 18 |
| cKO COX-2 | 20 | 152.22272 | 83.02933 | 18.56592 | 6893.86991 | 0 | 20 |
| Lyz2 | 24 | 195.42363 | 66.79996 | 13.63549 | 4462.23487 | 0 | 24 |

*Timepoint*

| | N | Mean | SD | SEM | Variance | Missing | NonMissing |
|---|---|---|---|---|---|---|---|
| 7 | 18 | 128.96895 | 33.30644 | 7.8504 | 1109.3187 | 0 | 18 |
| 10 | 22 | 152.42487 | 46.32148 | 9.87577 | 2145.67985 | 0 | 22 |
| 14 | 22 | 255.5575 | 68.17191 | 14.5343 | 4647.40909 | 0 | 22 |

*Gender*

| | N | Mean | SD | SEM | Variance | Missing | NonMissing |
|---|---|---|---|---|---|---|---|
| Male | 28 | 170.90349 | 65.50376 | 12.37905 | 4290.74299 | 0 | 28 |
| Female | 34 | 191.52222 | 83.12688 | 14.25614 | 6910.07773 | 0 | 34 |

*Genotype*Timepoint*

| | | N | Mean | SD | SEM | Variance | Missing | NonMissing |
|---|---|---|---|---|---|---|---|---|
| WT | 7 | 6 | 138.19563 | 12.26493 | 5.00714 | 150.4286 | 0 | 6 |
| | 10 | 6 | 190.60303 | 22.60385 | 9.22798 | 510.93419 | 0 | 6 |
| | 14 | 6 | 264.93998 | 87.40919 | 35.68465 | 7640.36722 | 0 | 6 |
| cKO COX-2 | 7 | 6 | 91.53861 | 27.6527 | 11.28917 | 764.67165 | 0 | 6 |
| | 10 | 7 | 106.65606 | 29.98516 | 11.33332 | 899.1097 | 0 | 7 |
| | 14 | 7 | 249.80434 | 55.49878 | 20.97657 | 3080.11453 | 0 | 7 |
| Lyz2 | 7 | 6 | 157.17262 | 10.89278 | 4.44696 | 118.65269 | 0 | 6 |
| | 10 | 9 | 162.57073 | 39.96026 | 13.32009 | 1596.82229 | 0 | 9 |
| | 14 | 9 | 253.77719 | 70.82078 | 23.60693 | 5015.58304 | 0 | 9 |

*Genotype*Gender*

| | | N | Mean | SD | SEM | Variance | Missing | NonMissin g |
|---|---|---|---|---|---|---|---|---|
| WT | Male | 9 | 165.87297 | 31.03157 | 10.34386 | 962.9584 | 0 | 9 |
| | Female | 9 | 229.9528 | 89.4429 | 29.8143 | 8000.03325 | 0 | 9 |
| cKO COX-2 | Male | 10 | 153.02972 | 98.33453 | 31.09611 | 9669.67927 | 0 | 10 |
| | Female | 10 | 151.41573 | 69.8756 | 22.0966 | 4882.59888 | 0 | 10 |
| Lyz2 | Male | 9 | 195.79377 | 38.92473 | 12.97491 | 1515.13439 | 0 | 9 |
| | Female | 15 | 195.20154 | 80.40449 | 20.76035 | 6464.88244 | 0 | 15 |

*Timepoint*Gender*

| | | N | Mean | SD | SEM | Variance | Missing | NonMissing |
|---|---|---|---|---|---|---|---|---|
| 7 | Male | 9 | 123.61606 | 38.33083 | 12.77694 | 1469.25272 | 0 | 9 |
| | Female | 9 | 134.32185 | 28.69807 | 9.56602 | 823.57912 | 0 | 9 |
| 10 | Male | 10 | 150.6613 | 41.6273 | 13.16371 | 1732.83246 | 0 | 10 |
| | Female | 12 | 153.89452 | 51.70437 | 14.92577 | 2673.34224 | 0 | 12 |
| 14 | Male | 9 | 240.68226 | 50.56739 | 16.8558 | 2557.06075 | 0 | 9 |
| | Female | 13 | 265.85575 | 78.40544 | 21.74576 | 6147.41237 | 0 | 13 |

*Genotype*Timepoint*Gender*

| | | | N | Mean | SD | SEM | Variance | Missing | NonMissing |
|---|---|---|---|---|---|---|---|---|---|
| WT | 7 | Male | 3 | 133.62127 | 13.98062 | 8.07172 | 195.45787 | 0 | 3 |
| | | Female | 3 | 142.77 | 10.85537 | 6.26735 | 117.83913 | 0 | 3 |
| | 10 | Male | 3 | 172.63043 | 6.59422 | 3.80717 | 43.4837 | 0 | 3 |
| | | Female | 3 | 208.57563 | 16.27294 | 9.39519 | 264.80871 | 0 | 3 |
| | 14 | Male | 3 | 191.3672 | 31.76799 | 18.34125 | 1009.20488 | 0 | 3 |
| | | Female | 3 | 338.51277 | 43.04474 | 24.85189 | 1852.84982 | 0 | 3 |
| cKO COX-2 | 7 | Male | 3 | 78.40877 | 22.25451 | 12.84865 | 495.26336 | 0 | 3 |
| | | Female | 3 | 104.66845 | 29.98729 | 17.31317 | 899.23743 | 0 | 3 |
| | 10 | Male | 4 | 107.23338 | 23.93707 | 11.96853 | 572.9833 | 0 | 4 |
| | | Female | 3 | 105.8863 | 42.85206 | 24.74065 | 1836.29877 | 0 | 3 |
| | 14 | Male | 3 | 288.71247 | 44.68855 | 25.80095 | 1997.06636 | 0 | 3 |
| | | Female | 4 | 220.62325 | 46.68648 | 23.34324 | 2179.62782 | 0 | 4 |
| Lyz2 | 7 | Male | 3 | 158.81813 | 10.57381 | 6.10479 | 111.80539 | 0 | 3 |
| | | Female | 3 | 155.5271 | 13.29297 | 7.6747 | 176.70315 | 0 | 3 |
| | 10 | Male | 3 | 186.59607 | 21.49169 | 12.40823 | 461.89267 | 0 | 3 |
| | | Female | 6 | 150.55807 | 43.01934 | 17.56257 | 1850.66361 | 0 | 6 |
| | 14 | Male | 3 | 241.9671 | 10.54427 | 6.08774 | 111.1817 | 0 | 3 |
| | | Female | 6 | 259.68223 | 88.62804 | 36.18225 | 7854.92981 | 0 | 6 |

*ANOVA*

*Overall ANOVA*

| | DF | Sum of Squares | Mean Square | F Value | P Value |
|---|---|---|---|---|---|
| Genotype | 2 | 25585.45503 | 12792.72752 | 7.41295 | 0.00168 |
| Timepoint | 2 | 180456.79931 | 90228.39965 | 52.28428 | <0.0001 |
| Gender | 1 | 2953.31749 | 2953.31749 | 1.71135 | 0.1976 |
| Genotype*Timepoint | 4 | 14995.36382 | 3748.84096 | 2.17233 | 0.0878 |
| Genotype*Gender | 2 | 17616.34599 | 8808.173 | 5.10404 | 0.01015 |
| Timepoint*Gender | 2 | 2839.93694 | 1419.96847 | 0.82282 | 0.44583 |
| Genotype*Timepoint*Gender | 4 | 24188.21761 | 6047.0544 | 3.50406 | 0.0144 |
| Model | 17 | 274478.47275 | 16145.79251 | 9.35594 | <0.0001 |
| Error | 44 | 75931.98635 | 1725.72696 | | |
| Corrected Total | 61 | 350410.4591 | | | |

At the 0.05 level, the population means of **Genotype** are **significantly** different. At the 0.05 level, the population means of **Timepoint** are **significantly** different. At the 0.05 level, the population means of **Gender** are **not significantly** different.

At the 0.05 level, the population means of **Genotype*Timepoint** are **not significantly** different. At the 0.05 level, the population means of **Genotype*Gender** are **significantly** different.

At the 0.05 level, the population means of **Timepoint*Gender** are **not significantly** different.

At the 0.05 level, the population means of **Genotype*Timepoint*Gender** are **significantly** different.

*Means Comparisons Tukey Test*

*Genotype*

| | MeanDiff | SEM | q Value | Prob | Alpha | Sig | LCL | UCL |
|---|---|---|---|---|---|---|---|---|
| WT cKO COX-2 | 46.99078 | 11.41967 | 5.81934 | 4.82526E-4 | 0.05 | 1 | 19.293 | 74.68857 |
| WT Lyz2 | 5.72143 | 11.16866 | 0.72447 | 0.86578 | 0.05 | 0 | -21.36754 | 32.8104 |
| cKO COX-2 Lyz2 | -41.26935 | 10.91187 | -5.34864 | 0.00133 | 0.05 | 1 | -67.7355 | -14.8032 |

*Timepoint*

| | MeanDiff | SEM | q Value | Prob | Alpha | Sig | LCL | UCL |
|---|---|---|---|---|---|---|---|---|
| 7 10 | -26.27769 | 11.29486 | -3.29019 | 0.0625 | 0.05 | 0 | -53.67276 | 1.11738 |
| 7 14 | -127.84188 | 11.29486 | -16.0069 | <0.0001 | 0.05 | 1 | -155.23695 | -100.44681 |
| 10 14 | -101.56419 | 10.91187 | -13.16304 | <0.0001 | 0.05 | 1 | -128.03034 | -75.09804 |

*Gender*

| | MeanDiff | SEM | q Value | Prob | Alpha | Sig | LCL | UCL |
|---|---|---|---|---|---|---|---|---|
| Male Female | -14.161 | 9.11917 | -2.19611 | 0.12762 | 0.05 | 0 | -32.53951 | 4.21751 |

*Genotype 's*

| | Mean | Groups | |
|---|---|---|---|
| WT | 197.91288 | A | |
| Lyz2 | 195.42363 | A | |
| cKO COX-2 | 152.22272 | | B |

Means that do not share a letter are significantly different.

*Timepoint 's*

| | Mean | Groups | |
|---|---|---|---|
| 14 | 255.5575 | A | |
| 10 | 152.42487 | | B |
| 7 | 128.96895 | | B |

Means that do not share a letter are significantly different.

*Gender 's Gr*

| | Mean | Groups |
|---|---|---|
| Female | 191.52222 | A |
| Male | 170.90349 | A |

Means that do not share a letter are significantly different.

*Interactions '*

| Genotype | Timepoint | Gender | Mean | Groups | | | | | | | |
|---|---|---|---|---|---|---|---|---|---|---|---|
| WT | 14 | Female | 338.51277 | A | | | | | | | |
| cKO COX-2 | 14 | Male | 288.71247 | A | B | | | | | | |
| Lyz2 | 14 | Female | 259.68223 | A | B | C | | | | | |
| Lyz2 | 14 | Male | 241.9671 | A | B | C | D | | | | |
| cKO COX-2 | 14 | Female | 220.62325 | | B | C | D | E | | | |
| WT | 10 | Female | 208.57563 | | B | C | D | E | F | | |
| WT | 14 | Male | 191.3672 | | B | C | D | E | F | G | |
| Lyz2 | 10 | Male | 186.59607 | | B | C | D | E | F | G | |
| WT | 10 | Male | 172.63043 | | | C | D | E | F | G | H |
| Lyz2 | 7 | Male | 158.81813 | | | | D | E | F | G | H |
| Lyz2 | 7 | Female | 155.5271 | | | | D | E | F | G | H |
| Lyz2 | 10 | Female | 150.55807 | | | | D | E | F | G | H |
| WT | 7 | Female | 142.77 | | | | D | E | F | G | H |
| WT | 7 | Male | 133.62127 | | | | | E | F | G | H |
| cKO COX-2 | 10 | Male | 107.23338 | | | | | | | G | H |
| cKO COX-2 | 10 | Female | 105.8863 | | | | | | F | G | H |
| cKO COX-2 | 7 | Female | 104.66845 | | | | | | F | G | H |
| cKO COX-2 | 7 | Male | 78.40877 | | | | | | | | H |

Means that do not share a letter are significantly different.

Sig equals 1 indicates that the difference of the means is significant at the 0.05 level. Sig equals 0 indicates that the difference of the means is not significant at the 0.05 level.

*ANOVAThreeWay (11/5/2024 18:31:0*

*Descriptive Statistics Genotype*

| | N | Mean | SD | SEM | Variance | Missing | NonMissing |
|---|---|---|---|---|---|---|---|
| WT | 19 | 1.51864 | 1.42619 | 0.32719 | 2.03403 | 0 | 19 |
| Lyz2 | 20 | 1.2014 | 1.44571 | 0.35064 | 2.09007 | 3 | 17 |
| cKO COX-2 | 17 | 0.34033 | 0.36646 | 0.09161 | 0.13429 | 1 | 16 |

*Timepoint*

| | N | Mean | SD | SEM | Variance | Missing | NonMissing |
|---|---|---|---|---|---|---|---|
| 10 | 31 | 1.50718 | 1.58239 | 0.29904 | 2.50397 | 3 | 28 |
| 14 | 25 | 0.52175 | 0.44386 | 0.0906 | 0.19702 | 1 | 24 |

*Gender*

| | N | Mean | SD | SEM | Variance | Missing | NonMissing |
|---|---|---|---|---|---|---|---|
| Male | 29 | 0.62216 | 0.43336 | 0.0834 | 0.1878 | 2 | 27 |
| Female | 27 | 1.51699 | 1.70063 | 0.34013 | 2.89213 | 2 | 25 |

*Genotype*Timepoint*

| | | N | Mean | SD | SEM | Variance | Missing | NonMissing |
|---|---|---|---|---|---|---|---|---|
| WT | 10 | 13 | 1.7356 | 1.68455 | 0.46721 | 2.8377 | 0 | 13 |
| | 14 | 6 | 1.04855 | 0.35278 | 0.14402 | 0.12445 | 0 | 6 |
| Lyz2 | 10 | 10 | 2.10182 | 1.709 | 0.60422 | 2.92069 | 2 | 8 |
| | 14 | 10 | 0.40102 | 0.30508 | 0.10169 | 0.09307 | 1 | 9 |
| cKO COX-2 | 10 | 8 | 0.4034 | 0.42321 | 0.15996 | 0.17911 | 1 | 7 |
| | 14 | 9 | 0.29128 | 0.33358 | 0.11119 | 0.11127 | 0 | 9 |

*Genotype*Gender*

| | | N | Mean | SD | SEM | Variance | Missing | NonMissing |
|---|---|---|---|---|---|---|---|---|
| WT | Male | 9 | 1.03636 | 0.29118 | 0.09706 | 0.08478 | 0 | 9 |
| | Female | 10 | 1.95268 | 1.88435 | 0.59588 | 3.55077 | 0 | 10 |
| Lyz2 | Male | 11 | 0.51011 | 0.31629 | 0.10543 | 0.10004 | 2 | 9 |
| | Female | 9 | 1.9791 | 1.8323 | 0.64782 | 3.35732 | 1 | 8 |
| cKO COX-2 | Male | 9 | 0.32001 | 0.34011 | 0.11337 | 0.11568 | 0 | 9 |
| | Female | 8 | 0.36647 | 0.42435 | 0.16039 | 0.18007 | 1 | 7 |

*Timepoint*Gender*

| | | N | Mean | SD | SEM | Variance | Missing | NonMissing |
|---|---|---|---|---|---|---|---|---|
| 10 | Male | 15 | 0.6801 | 0.38658 | 0.10332 | 0.14944 | 1 | 14 |
| | Female | 16 | 2.33427 | 1.89149 | 0.50552 | 3.57775 | 2 | 14 |
| 14 | Male | 14 | 0.55976 | 0.48669 | 0.13498 | 0.23686 | 1 | 13 |
| | Female | 11 | 0.47683 | 0.40596 | 0.1224 | 0.1648 | 0 | 11 |

*Genotype*Timepoint*Gender*

| | | | N | Mean | SD | SEM | Variance | Missing | NonMissing |
|---|---|---|---|---|---|---|---|---|---|
| WT | 10 | Male | 6 | 1.01702 | 0.20044 | 0.08183 | 0.04018 | 0 | 6 |
| | | Female | 7 | 2.35152 | 2.16402 | 0.81792 | 4.68298 | 0 | 7 |
| | 14 | Male | 3 | 1.07504 | 0.48511 | 0.28008 | 0.23533 | 0 | 3 |
| | | Female | 3 | 1.02206 | 0.27146 | 0.15673 | 0.07369 | 0 | 3 |

| | | | | | | | | | |
|---|---|---|---|---|---|---|---|---|---|
| Lyz2 | 10 | Male | 5 | 0.59429 | 0.16629 | 0.08314 | 0.02765 | 1 | 4 |
| | | Female | 5 | 3.60936 | 0.85255 | 0.42628 | 0.72684 | 1 | 4 |
| | 14 | Male | 6 | 0.44276 | 0.40815 | 0.18253 | 0.16659 | 1 | 5 |
| | | Female | 4 | 0.34884 | 0.1398 | 0.0699 | 0.01954 | 0 | 4 |
| cKO COX-2 | 10 | Male | 4 | 0.26054 | 0.28534 | 0.14267 | 0.08142 | 0 | 4 |
| | | Female | 4 | 0.59388 | 0.56565 | 0.32658 | 0.31996 | 1 | 3 |
| | 14 | Male | 5 | 0.36758 | 0.40488 | 0.18107 | 0.16393 | 0 | 5 |
| | | Female | 4 | 0.19591 | 0.23735 | 0.11867 | 0.05633 | 0 | 4 |

*ANOVA*

*Overall ANOVA*

| | DF | Sum of Squares | Mean Square | F Value | P Value |
|---|---|---|---|---|---|
| Genotype | 2 | 9.69445 | 4.84722 | 5.76807 | 0.00629 |
| Timepoint | 1 | 8.36112 | 8.36112 | 9.94952 | 0.00305 |
| Gender | 1 | 6.43595 | 6.43595 | 7.65862 | 0.00852 |
| Genotype*Timepoint | 2 | 5.19494 | 2.59747 | 3.09092 | 0.05646 |
| Genotype*Gender | 2 | 3.90806 | 1.95403 | 2.32525 | 0.11083 |
| Timepoint*Gender | 1 | 8.45233 | 8.45233 | 10.05805 | 0.00291 |
| Genotype*Timepoint*Gender | 2 | 3.57176 | 1.78588 | 2.12515 | 0.1327 |
| Model | 11 | 51.07388 | 4.64308 | 5.52514 | <0.0001 |
| Error | 40 | 33.61418 | 0.84035 | | |
| Corrected Total | 51 | 84.68805 | | | |

At the 0.05 level, the population means of **Genotype** are **significantly** different.
At the 0.05 level, the population means of **Timepoint** are **significantly** different.
At the 0.05 level, the population means of **Gender** are **significantly** different.
At the 0.05 level, the population means of **Genotype*Timepoint** are **not significantly** different.
At the 0.05 level, the population means of **Genotype*Gender** are **not significantly** different.
At the 0.05 level, the population means of **Timepoint*Gender** are **significantly** different.
At the 0.05 level, the population means of **Genotype*Timepoint*Gender** are **not significantly** different

*Means Comparisons Tukey Test*

*Genotype*

| | MeanDiff | SEM | q Value | Prob | Alpha | Sig | LCL | UCL |
|---|---|---|---|---|---|---|---|---|
| WT Lyz2 | 0.1176 | 0.27896 | 0.59617 | 0.90692 | 0.05 | 0 | -0.56137 | 0.79657 |
| WT cKO COX-2 | 1.01193 | 0.28494 | 5.02251 | 0.00281 | 0.05 | 1 | 0.31843 | 1.70543 |
| Lyz2 cKO COX-2 | 0.89433 | 0.28307 | 4.46805 | 0.00828 | 0.05 | 1 | 0.20537 | 1.5833 |

*Timepoint*

| | MeanDiff | SEM | q Value | Prob | Alpha | Sig | LCL | UCL |
|---|---|---|---|---|---|---|---|---|
| 10 14 | 0.82907 | 0.23053 | 5.08613 | 8.76968E-4 | 0.05 | 1 | 0.36316 | 1.29498 |

*Gender*

| | MeanDiff | SEM | q Value | Prob | Alpha | Sig | LCL | UCL |
|---|---|---|---|---|---|---|---|---|
| Male Female | -0.72739 | 0.23053 | -4.46233 | 0.00304 | 0.05 | 1 | -1.1933 | -0.26148 |

*Genotype 's*

| | Mean | Groups | |
|---|---|---|---|
| WT | 1.51864 | A | |
| Lyz2 | 1.2014 | A | |
| cKO COX-2 | 0.34033 | | B |

Means that do not share a letter are significantly different.

*Timepoint 's*

| | Mean | Groups | |
|---|---|---|---|
| 10 | 1.50718 | A | |
| 14 | 0.52175 | | B |

Means that do not share a letter are significantly different.

*Gender 's Gr*

| | Mean | Groups | |
|---|---|---|---|
| Female | 1.51699 | A | |
| Male | 0.62216 | | B |

Means that do not share a letter are significantly different.

*Interactions '*

| Genotype | Timepoint | Gender | Mean | Groups | | |
|---|---|---|---|---|---|---|
| Lyz2 | 10 | Female | 3.60936 | A | | |
| WT | 10 | Female | 2.35152 | A | B | |
| WT | 14 | Male | 1.07504 | | B | C |
| WT | 14 | Female | 1.02206 | | B | C |
| WT | 10 | Male | 1.01702 | | B | C |
| Lyz2 | 10 | Male | 0.59429 | | | C |
| cKO COX-2 | 10 | Female | 0.59388 | | B | C |
| Lyz2 | 14 | Male | 0.44276 | | | C |
| cKO COX-2 | 14 | Male | 0.36758 | | | C |
| Lyz2 | 14 | Female | 0.34884 | | | C |
| cKO COX-2 | 10 | Male | 0.26054 | | | C |
| cKO COX-2 | 14 | Female | 0.19591 | | | C |

Means that do not share a letter are significantly different.

Sig equals 1 indicates that the difference of the means is significant at the 0.05 level. Sig equals 0 indicates that the difference of the means is not significant at the 0.05 level.

*ANOVAThreeWay (11/5/2024 18:48:3*

*Descriptive Statistics Genotype*

| | N | Mean | SD | SEM | Variance | Missing | NonMissing |
|---|---|---|---|---|---|---|---|
| WT | 16 | 1.12327 | 0.51912 | 0.14986 | 0.26949 | 4 | 12 |
| Lyz2 | 15 | 1.40155 | 0.90643 | 0.26166 | 0.82161 | 3 | 12 |
| cKO COX-2 | 14 | 0.81785 | 0.90187 | 0.25013 | 0.81336 | 1 | 13 |

*Timepoint*

| | N | Mean | SD | SEM | Variance | Missing | NonMissing |
|---|---|---|---|---|---|---|---|
| 10 | 23 | 0.93854 | 0.71484 | 0.164 | 0.511 | 4 | 19 |
| 14 | 22 | 1.28321 | 0.89435 | 0.2108 | 0.79986 | 4 | 18 |

*Gender*

| | N | Mean | SD | SEM | Variance | Missing | NonMissing |
|---|---|---|---|---|---|---|---|
| Male | 23 | 1.30488 | 0.92247 | 0.21163 | 0.85094 | 4 | 19 |
| Female | 22 | 0.89651 | 0.6433 | 0.15163 | 0.41383 | 4 | 18 |

*Genotype*Timepoint*

| | | N | Mean | SD | SEM | Variance | Missing | NonMissing |
|---|---|---|---|---|---|---|---|---|
| WT | 10 | 9 | 1.13993 | 0.59984 | 0.24489 | 0.35981 | 3 | 6 |
| | 14 | 7 | 1.10661 | 0.48207 | 0.19681 | 0.23239 | 1 | 6 |
| Lyz2 | 10 | 6 | 1.41811 | 0.64804 | 0.26456 | 0.41996 | 0 | 6 |
| | 14 | 9 | 1.385 | 1.17768 | 0.48079 | 1.38693 | 3 | 6 |
| cKO COX-2 | 10 | 8 | 0.35486 | 0.46389 | 0.17533 | 0.21519 | 1 | 7 |
| | 14 | 6 | 1.35801 | 1.02157 | 0.41706 | 1.04361 | 0 | 6 |

*Genotype*Gender*

| | | N | Mean | SD | SEM | Variance | Missing | NonMissing |
|---|---|---|---|---|---|---|---|---|
| WT | Male | 8 | 1.16697 | 0.61275 | 0.25016 | 0.37547 | 2 | 6 |
| | Female | 8 | 1.07956 | 0.46133 | 0.18834 | 0.21282 | 2 | 6 |
| Lyz2 | Male | 8 | 1.61453 | 1.04057 | 0.42481 | 1.08279 | 2 | 6 |
| | Female | 7 | 1.18858 | 0.78479 | 0.32039 | 0.61589 | 1 | 6 |
| cKO COX-2 | Male | 7 | 1.15768 | 1.09427 | 0.4136 | 1.19744 | 0 | 7 |
| | Female | 7 | 0.4214 | 0.40604 | 0.16576 | 0.16487 | 1 | 6 |

*Timepoint*Gender*

| | | N | Mean | SD | SEM | Variance | Missing | NonMissing |
|---|---|---|---|---|---|---|---|---|
| 10 | Male | 11 | 0.81176 | 0.63494 | 0.20078 | 0.40314 | 1 | 10 |
| | Female | 12 | 1.07942 | 0.80858 | 0.26953 | 0.6538 | 3 | 9 |
| 14 | Male | 12 | 1.8528 | 0.9052 | 0.30173 | 0.81938 | 3 | 9 |
| | Female | 10 | 0.71361 | 0.38772 | 0.12924 | 0.15033 | 1 | 9 |

*Genotype*Timepoint*Gender*

| | | | N | Mean | SD | SEM | Variance | Missing | NonMissing |
|---|---|---|---|---|---|---|---|---|---|
| WT | 10 | Male | 4 | 1.1466 | 0.6731 | 0.38862 | 0.45307 | 1 | 3 |
| | | Female | 5 | 1.13326 | 0.66808 | 0.38572 | 0.44633 | 2 | 3 |
| | 14 | Male | 4 | 1.18735 | 0.69596 | 0.40181 | 0.48436 | 1 | 3 |
| | | Female | 3 | 1.02587 | 0.27761 | 0.16028 | 0.07707 | 0 | 3 |

| | | | | | | | | | |
|---|---|---|---|---|---|---|---|---|---|
| Lyz2 | 10 | Male | 3 | 1.11494 | 0.50577 | 0.29201 | 0.2558 | 0 | 3 |
| | | Female | 3 | 1.72128 | 0.71997 | 0.41568 | 0.51836 | 0 | 3 |
| | 14 | Male | 5 | 2.11412 | 1.30477 | 0.75331 | 1.70242 | 2 | 3 |
| | | Female | 4 | 0.65588 | 0.41239 | 0.23809 | 0.17007 | 1 | 3 |
| cKO COX-2 | 10 | Male | 4 | 0.33323 | 0.47697 | 0.23848 | 0.2275 | 0 | 4 |
| | | Female | 4 | 0.38371 | 0.54968 | 0.31736 | 0.30215 | 1 | 3 |
| | 14 | Male | 3 | 2.25693 | 0.28123 | 0.16237 | 0.07909 | 0 | 3 |
| | | Female | 3 | 0.45909 | 0.3252 | 0.18775 | 0.10575 | 0 | 3 |

*ANOVA*

*Overall ANOVA*

| | DF | Sum of Squares | Mean Square | F Value | P Value |
|---|---|---|---|---|---|
| Genotype | 2 | 1.82831 | 0.91416 | 2.31516 | 0.11955 |
| Timepoint | 1 | 0.88921 | 0.88921 | 2.25199 | 0.14597 |
| Gender | 1 | 1.96483 | 1.96483 | 4.97605 | 0.03491 |
| Genotype*Timepoint | 2 | 2.22593 | 1.11296 | 2.81865 | 0.07873 |
| Genotype*Gender | 2 | 0.96641 | 0.4832 | 1.22374 | 0.31115 |
| Timepoint*Gender | 1 | 4.21072 | 4.21072 | 10.66392 | 0.00316 |
| Genotype*Timepoint*Gender | 2 | 1.66451 | 0.83225 | 2.10773 | 0.14259 |
| Model | 11 | 14.02219 | 1.27474 | 3.22837 | 0.00734 |
| Error | 25 | 9.87142 | 0.39486 | | |
| Corrected Total | 36 | 23.89362 | | | |

At the 0.05 level, the population means of **Genotype** are **not significantly** different.
At the 0.05 level, the population means of **Timepoint** are **not significantly** different.
At the 0.05 level, the population means of **Gender** are **significantly** different.
At the 0.05 level, the population means of **Genotype*Timepoint** are **not significantly** different.
At the 0.05 level, the population means of **Genotype*Gender** are **not significantly** different.
At the 0.05 level, the population means of **Timepoint*Gender** are **significantly** different.
At the 0.05 level, the population means of **Genotype*Timepoint*Gender** are **not significantly** different.

*Means Comparisons Tukey Test*

*Genotype*

| | MeanDiff | SEM | q Value | Prob | Alpha | Sig | LCL | UCL |
|---|---|---|---|---|---|---|---|---|
| WT Lyz2 | -0.27829 | 0.21087 | -1.86635 | 0.39776 | 0.05 | 0 | -0.80352 | 0.24695 |
| WT cKO COX-2 | 0.26503 | 0.20755 | 1.80588 | 0.42093 | 0.05 | 0 | -0.25193 | 0.78199 |
| Lyz2 cKO COX-2 | 0.54331 | 0.20755 | 3.70209 | 0.03805 | 0.05 | 1 | 0.02635 | 1.06028 |

*Timepoint*

| | MeanDiff | SEM | q Value | Prob | Alpha | Sig | LCL | UCL |
|---|---|---|---|---|---|---|---|---|
| 10 14 | -0.31104 | 0.17037 | -2.58184 | 0.07987 | 0.05 | 0 | -0.66192 | 0.03985 |

*Gender*

| | MeanDiff | SEM | q Value | Prob | Alpha | Sig | LCL | UCL |
|---|---|---|---|---|---|---|---|---|
| Male Female | 0.46235 | 0.17037 | 3.83785 | 0.01187 | 0.05 | 1 | 0.11146 | 0.81324 |

*Genotype 's*

| | Mean | Groups | |
|---|---|---|---|
| Lyz2 | 1.40155 | A | |
| WT | 1.12327 | A | B |
| cKO COX-2 | 0.81785 | | B |

Means that do not share a letter are significantly different.

*Timepoint 's*

| | Mean | Groups |
|---|---|---|
| 14 | 1.28321 | A |
| 10 | 0.93854 | A |

Means that do not share a letter are significantly different.

*Gender 's Gr*

| | Mean | Groups | |
|---|---|---|---|
| Male | 1.30488 | A | |
| Female | 0.89651 | | B |

Means that do not share a letter are significantly different.

*Interactions '*

| Genotype | Timepoint | Gender | Mean | Groups | | |
|---|---|---|---|---|---|---|
| cKO COX-2 | 14 | Male | 2.25693 | A | | |
| Lyz2 | 14 | Male | 2.11412 | A | B | |
| Lyz2 | 10 | Female | 1.72128 | A | B | C |
| WT | 14 | Male | 1.18735 | A | B | C |
| WT | 10 | Male | 1.1466 | A | B | C |
| WT | 10 | Female | 1.13326 | A | B | C |
| Lyz2 | 10 | Male | 1.11494 | A | B | C |
| WT | 14 | Female | 1.02587 | A | B | C |
| Lyz2 | 14 | Female | 0.65588 | | B | C |
| cKO COX-2 | 14 | Female | 0.45909 | | | C |
| cKO COX-2 | 10 | Female | 0.38371 | | | C |
| cKO COX-2 | 10 | Male | 0.33323 | | | C |

Means that do not share a letter are significantly different.

Sig equals 1 indicates that the difference of the means is significant at the 0.05 level. Sig equals 0 indicates that the difference of the means is not significant at the 0.05 level.

**S6-A. F4-80+ Macrophage Density:**
**(F4-80+ cells per callus mm$^2$)**

*ANOVAThreeWay (11/5/2024 17:57:3*

*Descriptive Statistics Genotype*

| | N | Mean | SD | SEM | Variance | Missing | NonMissing |
|---|---|---|---|---|---|---|---|
| WT | 18 | 185.58452 | 20.41972 | 4.81297 | 416.96483 | 0 | 18 |
| cKO COX-2 | 18 | 118.74742 | 38.86128 | 9.15969 | 1510.1992 | 0 | 18 |
| Lyz2 | 18 | 171.15506 | 23.65152 | 5.57472 | 559.39425 | 0 | 18 |

*Timepoint*

| | N | Mean | SD | SEM | Variance | Missing | NonMissing |
|---|---|---|---|---|---|---|---|
| 7 | 18 | 136.00342 | 37.94526 | 8.94378 | 1439.84272 | 0 | 18 |
| 10 | 18 | 153.83125 | 37.21454 | 8.77155 | 1384.92183 | 0 | 18 |
| 14 | 18 | 185.65233 | 30.68795 | 7.23322 | 941.75006 | 0 | 18 |

*Gender*

| | N | Mean | SD | SEM | Variance | Missing | NonMissing |
|---|---|---|---|---|---|---|---|
| Male | 27 | 161.20339 | 39.27846 | 7.55914 | 1542.79779 | 0 | 27 |
| Female | 27 | 155.78794 | 42.19672 | 8.12076 | 1780.5634 | 0 | 27 |

*Genotype*Timepoint*

| | | N | Mean | SD | SEM | Variance | Missing | NonMissing |
|---|---|---|---|---|---|---|---|---|
| WT | 7 | 6 | 163.44408 | 12.83464 | 5.23972 | 164.72795 | 0 | 6 |
| | 10 | 6 | 189.5085 | 4.92987 | 2.01261 | 24.3036 | 0 | 6 |
| | 14 | 6 | 203.80098 | 14.95746 | 6.10636 | 223.72555 | 0 | 6 |
| cKO COX-2 | 7 | 6 | 85.29382 | 7.64989 | 3.12305 | 58.52082 | 0 | 6 |
| | 10 | 6 | 109.9768 | 14.84473 | 6.06034 | 220.36604 | 0 | 6 |
| | 14 | 6 | 160.97163 | 35.79174 | 14.61192 | 1281.04858 | 0 | 6 |
| Lyz2 | 7 | 6 | 159.27237 | 5.75666 | 2.35015 | 33.13918 | 0 | 6 |
| | 10 | 6 | 162.00845 | 23.42072 | 9.56147 | 548.52992 | 0 | 6 |
| | 14 | 6 | 192.18437 | 22.7983 | 9.30737 | 519.7626 | 0 | 6 |

*Genotype*Gender*

| | | N | Mean | SD | SEM | Variance | Missing | NonMissing |
|---|---|---|---|---|---|---|---|---|
| WT | Male | 9 | 183.45246 | 21.15489 | 7.05163 | 447.52942 | 0 | 9 |
| | Female | 9 | 187.71659 | 20.69524 | 6.89841 | 428.293 | 0 | 9 |
| cKO COX-2 | Male | 9 | 124.51387 | 41.99454 | 13.99818 | 1763.54168 | 0 | 9 |
| | Female | 9 | 112.98097 | 37.02452 | 12.34151 | 1370.81475 | 0 | 9 |
| Lyz2 | Male | 9 | 175.64384 | 22.29673 | 7.43224 | 497.14436 | 0 | 9 |
| | Female | 9 | 166.66628 | 25.42111 | 8.4737 | 646.23277 | 0 | 9 |

*Timepoint*Gender*

| | | N | Mean | SD | SEM | Variance | Missing | NonMissing |
|---|---|---|---|---|---|---|---|---|
| 7 | Male | 9 | 137.39344 | 35.95531 | 11.9851 | 1292.78464 | 0 | 9 |
| | Female | 9 | 134.6134 | 41.98254 | 13.99418 | 1762.53378 | 0 | 9 |
| 10 | Male | 9 | 154.72893 | 38.41201 | 12.804 | 1475.48283 | 0 | 9 |
| | Female | 9 | 152.93357 | 38.28398 | 12.76133 | 1465.66293 | 0 | 9 |
| 14 | Male | 9 | 191.48779 | 23.0022 | 7.6674 | 529.10132 | 0 | 9 |
| | Female | 9 | 179.81687 | 37.35638 | 12.45213 | 1395.4992 | 0 | 9 |

*Genotype*Timepoint*Gender*

| | | | N | Mean | SD | SEM | Variance | Missing | NonMissing |
|---|---|---|---|---|---|---|---|---|---|
| WT | 7 | Male | 3 | 159.9234 | 17.99199 | 10.38768 | 323.71162 | 0 | 3 |
| | | Female | 3 | 166.96477 | 7.13601 | 4.11998 | 50.92262 | 0 | 3 |
| | 10 | Male | 3 | 187.34673 | 4.0129 | 2.31685 | 16.10339 | 0 | 3 |
| | | Female | 3 | 191.67027 | 5.53497 | 3.19562 | 30.63591 | 0 | 3 |
| | 14 | Male | 3 | 203.08723 | 4.34087 | 2.5062 | 18.84313 | 0 | 3 |
| | | Female | 3 | 204.51473 | 23.21513 | 13.40326 | 538.94242 | 0 | 3 |
| cKO COX-2 | 7 | Male | 3 | 91.35207 | 5.04402 | 2.91216 | 25.4421 | 0 | 3 |
| | | Female | 3 | 79.23557 | 3.27914 | 1.89321 | 10.75277 | 0 | 3 |
| | 10 | Male | 3 | 106.16723 | 16.57831 | 9.57149 | 274.84051 | 0 | 3 |
| | | Female | 3 | 113.78637 | 15.24914 | 8.80409 | 232.53621 | 0 | 3 |
| | 14 | Male | 3 | 176.0223 | 24.89478 | 14.37301 | 619.75015 | 0 | 3 |
| | | Female | 3 | 145.92097 | 43.62687 | 25.18798 | 1903.3036 | 0 | 3 |
| Lyz2 | 7 | Male | 3 | 160.90487 | 7.18981 | 4.15104 | 51.69333 | 0 | 3 |
| | | Female | 3 | 157.63987 | 4.81243 | 2.77846 | 23.15946 | 0 | 3 |
| | 10 | Male | 3 | 170.67283 | 9.82019 | 5.66969 | 96.43611 | 0 | 3 |
| | | Female | 3 | 153.34407 | 32.39867 | 18.70538 | 1049.67406 | 0 | 3 |
| | 14 | Male | 3 | 195.35383 | 29.91321 | 17.2704 | 894.79988 | 0 | 3 |
| | | Female | 3 | 189.0149 | 19.35123 | 11.17244 | 374.47007 | 0 | 3 |

*ANOVA*

*Overall ANOVA*

| | DF | Sum of Squares | Mean Square | F Value | P Value |
|---|---|---|---|---|---|
| Genotype | 2 | 44531.81534 | 22265.90767 | 61.31966 | <0.0001 |
| Timepoint | 2 | 22772.55754 | 11386.27877 | 31.35748 | <0.0001 |
| Gender | 1 | 395.91502 | 395.91502 | 1.09034 | 0.30336 |
| Genotype*Timepoint | 4 | 4128.31195 | 1032.07799 | 2.84231 | 0.03806 |
| Genotype*Gender | 2 | 647.12791 | 323.56396 | 0.89109 | 0.41906 |
| Timepoint*Gender | 2 | 266.31584 | 133.15792 | 0.36671 | 0.69557 |
| Genotype*Timepoint*Gender | 4 | 989.22777 | 247.30694 | 0.68108 | 0.60962 |
| Model | 17 | 73731.27137 | 4337.13361 | 11.94434 | <0.0001 |
| Error | 36 | 13072.03467 | 363.11207 | | |
| Corrected Total | 53 | 86803.30605 | | | |

At the 0.05 level, the population means of **Genotype** are **significantly** different.
At the 0.05 level, the population means of **Timepoint** are **significantly** different.
At the 0.05 level, the population means of **Gender** are **not significantly** different.
At the 0.05 level, the population means of **Genotype*Timepoint** are **significantly** different.
At the 0.05 level, the population means of **Genotype*Gender** are **not significantly** different.
At the 0.05 level, the population means of **Timepoint*Gender** are **not significantly** different.
At the 0.05 level, the population means of **Genotype*Timepoint*Gender** are **not significantly** different.

*Means Comparisons Tukey Test*

*Genotype*

| | MeanDiff | SEM | q Value | Prob | Alpha | Sig | LCL | UCL |
|---|---|---|---|---|---|---|---|---|
| WT cKO COX-2 | 66.83711 | 5.18625 | 18.22549 | <0.0001 | 0.05 | 1 | 54.16054 | 79.51367 |
| WT Lyz2 | 14.42946 | 5.18625 | 3.9347 | 0.02269 | 0.05 | 1 | 1.7529 | 27.10602 |
| cKO COX-2 Lyz2 | -52.40764 | 5.18625 | -14.29079 | <0.0001 | 0.05 | 1 | -65.08421 | -39.73108 |

*Timepoint*

| | MeanDiff | SEM | q Value | Prob | Alpha | Sig | LCL | UCL |
|---|---|---|---|---|---|---|---|---|
| 7 10 | -17.82783 | 5.18625 | -4.86138 | 0.00418 | 0.05 | 1 | -30.50439 | -5.15127 |
| 7 14 | -49.64891 | 5.18625 | -13.53852 | <0.0001 | 0.05 | 1 | -62.32547 | -36.97234 |
| 10 14 | -31.82108 | 5.18625 | -8.67714 | <0.0001 | 0.05 | 1 | -44.49764 | -19.14452 |

*Gender*

| | MeanDiff | SEM | q Value | Prob | Alpha | Sig | LCL | UCL |
|---|---|---|---|---|---|---|---|---|
| Male Female | 5.41544 | 4.23456 | 1.80859 | 0.20913 | 0.05 | 0 | -3.17266 | 14.00355 |

*Genotype 's*

| | Mean | Groups | | |
|---|---|---|---|---|
| WT | 185.58452 | A | | |
| Lyz2 | 171.15506 | | B | |
| cKO COX-2 | 118.74742 | | | C |

Means that do not share a letter are significantly different.

*Timepoint 's*

| | Mean | Groups | | |
|---|---|---|---|---|
| 14 | 185.65233 | A | | |
| 10 | 153.83125 | | B | |
| 7 | 136.00342 | | | C |

Means that do not share a letter are significantly different.

*Gender 's Gr*

| | Mean | Groups |
|---|---|---|
| Male | 161.20339 | A |
| Female | 155.78794 | A |

Means that do not share a letter are significantly different.

*Interactions '*

| Genotype | Timepoint | Gender | Mean | Groups | | | | | |
|---|---|---|---|---|---|---|---|---|---|
| WT | 14 | Female | 204.51473 | A | | | | | |
| WT | 14 | Male | 203.08723 | A | | | | | |
| Lyz2 | 14 | Male | 195.35383 | A | B | | | | |
| WT | 10 | Female | 191.67027 | A | B | C | | | |
| Lyz2 | 14 | Female | 189.0149 | A | B | C | | | |
| WT | 10 | Male | 187.34673 | A | B | C | | | |
| cKO COX-2 | 14 | Male | 176.0223 | A | B | C | | | |
| Lyz2 | 10 | Male | 170.67283 | A | B | C | | | |
| WT | 7 | Female | 166.96477 | A | B | C | | | |
| Lyz2 | 7 | Male | 160.90487 | A | B | C | D | | |
| WT | 7 | Male | 159.9234 | A | B | C | D | | |
| Lyz2 | 7 | Female | 157.63987 | A | B | C | D | | |
| Lyz2 | 10 | Female | 153.34407 | | B | C | D | E | |
| cKO COX-2 | 14 | Female | 145.92097 | | | C | D | E | |
| cKO COX-2 | 10 | Female | 113.78637 | | | | D | E | F |
| cKO COX-2 | 10 | Male | 106.16723 | | | | | E | F |
| cKO COX-2 | 7 | Male | 91.35207 | | | | | | F |
| cKO COX-2 | 7 | Female | 79.23557 | | | | | | F |

Means that do not share a letter are significantly different.

Sig equals 1 indicates that the difference of the means is significant at the 0.05 level. Sig equals 0 indicates that the difference of the means is not significant at the 0.05 level.

**S6-B. F4-80$^{+}$ Macrophage EO Zone Density: (F4-80+ cells per Bone Perimeter mm)**

*Descriptive Statistics Genotype*

| | N | Mean | SD | SEM | Variance | Missing | NonMissing |
|---|---|---|---|---|---|---|---|
| WT | 18 | 80.12072 | 24.41888 | 5.75558 | 596.28165 | 0 | 18 |
| Lyz2 | 18 | 76.32356 | 20.49835 | 4.83151 | 420.18232 | 0 | 18 |
| cKO COX-2 | 18 | 56.82997 | 18.8486 | 4.44266 | 355.26976 | 0 | 18 |

*Timepoint*

| | N | Mean | SD | SEM | Variance | Missing | NonMissing |
|---|---|---|---|---|---|---|---|
| 7 dpf | 18 | 62.62448 | 20.24421 | 4.77161 | 409.82795 | 0 | 18 |
| 10 dpf | 18 | 74.70104 | 19.12987 | 4.50895 | 365.95187 | 0 | 18 |
| 14 dpf | 18 | 75.94872 | 28.49444 | 6.7162 | 811.93337 | 0 | 18 |

*Gender*

| | N | Mean | SD | SEM | Variance | Missing | NonMissing |
|---|---|---|---|---|---|---|---|
| Male | 27 | 75.65222 | 25.3667 | 4.88182 | 643.46943 | 0 | 27 |
| Female | 27 | 66.53061 | 20.65027 | 3.97415 | 426.43385 | 0 | 27 |

*Genotype*Timepoint*

| | | N | Mean | SD | SEM | Variance | Missing | NonMissing |
|---|---|---|---|---|---|---|---|---|
| WT | 7 dpf | 6 | 66.17297 | 8.04597 | 3.28475 | 64.73759 | 0 | 6 |
| | 10 dpf | 6 | 77.40758 | 10.33626 | 4.21976 | 106.83822 | 0 | 6 |
| | 14 dpf | 6 | 96.7816 | 35.78266 | 14.60821 | 1280.39877 | 0 | 6 |
| Lyz2 | 7 dpf | 6 | 79.75088 | 19.17192 | 7.8269 | 367.56254 | 0 | 6 |
| | 10 dpf | 6 | 79.67308 | 25.64609 | 10.46997 | 657.72176 | 0 | 6 |
| | 14 dpf | 6 | 69.5467 | 17.90714 | 7.31056 | 320.66556 | 0 | 6 |
| cKO COX-2 | 7 dpf | 6 | 41.9496 | 9.00486 | 3.67622 | 81.08755 | 0 | 6 |
| | 10 dpf | 6 | 67.02247 | 19.24754 | 7.85778 | 370.46789 | 0 | 6 |
| | 14 dpf | 6 | 61.51785 | 18.42866 | 7.52347 | 339.6154 | 0 | 6 |

*Genotype*Gender*

| | | N | Mean | SD | SEM | Variance | Missing | NonMissing |
|---|---|---|---|---|---|---|---|---|
| WT | Male | 9 | 81.40414 | 26.28755 | 8.76252 | 691.03504 | 0 | 9 |
| | Female | 9 | 78.83729 | 23.92399 | 7.97466 | 572.3573 | 0 | 9 |
| Lyz2 | Male | 9 | 90.10984 | 15.69446 | 5.23149 | 246.31603 | 0 | 9 |
| | Female | 9 | 62.53727 | 14.79637 | 4.93212 | 218.93243 | 0 | 9 |
| cKO COX-2 | Male | 9 | 55.44267 | 20.54445 | 6.84815 | 422.07429 | 0 | 9 |
| | Female | 9 | 58.21728 | 18.12577 | 6.04192 | 328.54356 | 0 | 9 |

*Timepoint*Gender*

| | | N | Mean | SD | SEM | Variance | Missing | NonMissing |
|---|---|---|---|---|---|---|---|---|
| 7 dpf | Male | 9 | 65.9971 | 24.64927 | 8.21642 | 607.58636 | 0 | 9 |
| | Female | 9 | 59.25187 | 15.41769 | 5.13923 | 237.70531 | 0 | 9 |
| 10 dpf | Male | 9 | 81.78628 | 22.58109 | 7.52703 | 509.90563 | 0 | 9 |
| | Female | 9 | 67.61581 | 12.4415 | 4.14717 | 154.7909 | 0 | 9 |
| 14 dpf | Male | 9 | 79.17328 | 28.5067 | 9.50223 | 812.63207 | 0 | 9 |
| | Female | 9 | 72.72416 | 29.82166 | 9.94055 | 889.3313 | 0 | 9 |

*Genotype*Timepoint*Gender*

| | | | N | Mean | SD | SEM | Variance | Missing | NonMissing |
|---|---|---|---|---|---|---|---|---|---|
| WT | 7 dpf | Male | 3 | 67.41293 | 9.55239 | 5.51507 | 91.24815 | 0 | 3 |
| | | Female | 3 | 64.933 | 8.12301 | 4.68982 | 65.98328 | 0 | 3 |
| | 10 dpf | Male | 3 | 84.09727 | 9.51045 | 5.49086 | 90.44863 | 0 | 3 |
| | | Female | 3 | 70.7179 | 6.51086 | 3.75905 | 42.39132 | 0 | 3 |
| | 14 dpf | Male | 3 | 92.70223 | 45.67782 | 26.3721 | 2086.46281 | 0 | 3 |
| | | Female | 3 | 100.86097 | 32.62837 | 18.838 | 1064.61043 | 0 | 3 |
| Lyz2 | 7 dpf | Male | 3 | 90.59247 | 17.1378 | 9.89451 | 293.70423 | 0 | 3 |
| | | Female | 3 | 68.9093 | 16.51007 | 9.53209 | 272.58234 | 0 | 3 |
| | 10 dpf | Male | 3 | 98.65117 | 19.71362 | 11.38166 | 388.62683 | 0 | 3 |
| | | Female | 3 | 60.695 | 13.23536 | 7.64144 | 175.17463 | 0 | 3 |
| | 14 dpf | Male | 3 | 81.0859 | 8.42645 | 4.86501 | 71.00505 | 0 | 3 |
| | | Female | 3 | 58.0075 | 18.19889 | 10.50713 | 331.19944 | 0 | 3 |
| cKO COX-2 | 7 dpf | Male | 3 | 39.9859 | 10.95967 | 6.32757 | 120.1143 | 0 | 3 |
| | | Female | 3 | 43.9133 | 8.4283 | 4.86608 | 71.03623 | 0 | 3 |
| | 10 dpf | Male | 3 | 62.6104 | 23.96514 | 13.83628 | 574.32792 | 0 | 3 |
| | | Female | 3 | 71.43453 | 17.13017 | 9.89011 | 293.44282 | 0 | 3 |
| | 14 dpf | Male | 3 | 63.7317 | 21.33918 | 12.32018 | 455.36079 | 0 | 3 |
| | | Female | 3 | 59.304 | 19.46726 | 11.23943 | 378.97432 | 0 | 3 |

*ANOVA*

*Overall ANOVA*

| | DF | Sum of Squares | Mean Square | F Value | P Value |
|---|---|---|---|---|---|
| Genotype | 2 | 5621.262 | 2810.631 | 7.36764 | 0.00208 |
| Timepoint | 2 | 1949.61125 | 974.80562 | 2.55531 | 0.09168 |
| Gender | 1 | 1123.25024 | 1123.25024 | 2.94443 | 0.09477 |
| Genotype*Timepoint | 4 | 3424.3857 | 856.09642 | 2.24413 | 0.08351 |
| Genotype*Gender | 2 | 2362.15393 | 1181.07696 | 3.09601 | 0.05745 |
| Timepoint*Gender | 2 | 172.2614 | 86.1307 | 0.22578 | 0.79902 |
| Genotype*Timepoint*Gender | 4 | 554.42388 | 138.60597 | 0.36333 | 0.83305 |
| Model | 17 | 15207.34839 | 894.54991 | 2.34493 | 0.01559 |
| Error | 36 | 13733.38702 | 381.48297 | | |
| Corrected Total | 53 | 28940.73542 | | | |

**At the 0.05 level, the population means of Genotype are significantly different.**
**At the 0.05 level, the population means of Timepoint are not significantly different.**
**At the 0.05 level, the population means of Gender are not significantly different.**
**At the 0.05 level, the population means of Genotype*Timepoint are not significantly different. At the 0.05 level, the population means of Genotype*Gender are not significantly different.**
**At the 0.05 level, the population means of Timepoint*Gender are not significantly different.**
**At the 0.05 level, the population means of Genotype*Timepoint*Gender are not significantly different.**

*Means Comparisons Tukey Test*

*Genotype*

| | MeanDiff | SEM | q Value | Prob | Alpha | Sig | LCL | UCL |
|---|---|---|---|---|---|---|---|---|
| WT Lyz2 | 3.79716 | 5.31583 | 1.01019 | 0.75667 | 0.05 | 0 | -9.19612 | 16.79044 |
| WT cKO COX-2 | 23.29074 | 5.31583 | 6.19623 | 2.81994E-4 | 0.05 | 1 | 10.29747 | 36.28402 |
| Lyz2 cKO COX-2 | 19.49358 | 5.31583 | 5.18604 | 0.00222 | 0.05 | 1 | 6.50031 | 32.48686 |

*Timepoint*

| | MeanDiff | SEM | q Value | Prob | Alpha | Sig | LCL | UCL |
|---|---|---|---|---|---|---|---|---|
| 7 dpf 10 dpf | -12.07656 | 5.31583 | -3.21283 | 0.07289 | 0.05 | 0 | -25.06984 | 0.91672 |
| 7 dpf 14 dpf | -13.32423 | 5.31583 | -3.54476 | 0.04345 | 0.05 | 1 | -26.31751 | -0.33096 |
| 10 dpf 14 dpf | -1.24767 | 5.31583 | -0.33193 | 0.97011 | 0.05 | 0 | -14.24095 | 11.74561 |

*Gender*

| | MeanDiff | SEM | q Value | Prob | Alpha | Sig | LCL | UCL |
|---|---|---|---|---|---|---|---|---|
| Male Female | 9.12161 | 4.34035 | 2.97209 | 0.04265 | 0.05 | 1 | 0.31894 | 17.92428 |

*Genotype 's G*

| | Mean | Groups | |
|---|---|---|---|
| WT | 80.12072 | A | |
| Lyz2 | 76.32356 | A | |
| cKO COX-2 | 56.82997 | | B |

Means that do not share a letter are significantly different.

*Timepoint 's G*

| | Mean | Groups | |
|---|---|---|---|
| 14 dpf | 75.94872 | A | |
| 10 dpf | 74.70104 | A | B |
| 7 dpf | 62.62448 | | B |

Means that do not share a letter are significantly different.

*Gender 's Gro*

| | Mean | Groups | |
|---|---|---|---|
| Male | 75.65222 | A | |
| Female | 66.53061 | | B |

Means that do not share a letter are significantly different.

*Interactions 's*

| Genotype | Timepoint | Gender | Mean | Groups | | |
|---|---|---|---|---|---|---|
| WT | 14 dpf | Female | 100.86097 | A | | |
| Lyz2 | 10 dpf | Male | 98.65117 | A | | |
| WT | 14 dpf | Male | 92.70223 | A | B | |
| Lyz2 | 7 dpf | Male | 90.59247 | A | B | |
| WT | 10 dpf | Male | 84.09727 | A | B | C |
| Lyz2 | 14 dpf | Male | 81.0859 | A | B | C |
| cKO COX-2 | 10 dpf | Female | 71.43453 | A | B | C |
| WT | 10 dpf | Female | 70.7179 | A | B | C |
| Lyz2 | 7 dpf | Female | 68.9093 | A | B | C |
| WT | 7 dpf | Male | 67.41293 | A | B | C |
| WT | 7 dpf | Female | 64.933 | A | B | C |
| cKO COX-2 | 14 dpf | Male | 63.7317 | A | B | C |
| cKO COX-2 | 10 dpf | Male | 62.6104 | A | B | C |
| Lyz2 | 10 dpf | Female | 60.695 | A | B | C |
| cKO COX-2 | 14 dpf | Female | 59.304 | A | B | C |
| Lyz2 | 14 dpf | Female | 58.0075 | A | B | C |
| cKO COX-2 | 7 dpf | Female | 43.9133 | | B | C |
| cKO COX-2 | 7 dpf | Male | 39.9859 | | | C |

Means that do not share a letter are significantly different.

Sig equals 1 indicates that the difference of the means is significant at the 0.05 level. Sig equals 0 indicates that the difference of the means is not significant at the 0.05 level.

**S6-C. Relative F4/80 (*Adgre1*) mRNA Levels (RTqPCR)**

*ANOVAThreeWay (11/5/2024 18:51:3*

*Descriptive Statistics Genotype*

| | N | Mean | SD | SEM | Variance | Missing | NonMissing |
|---|---|---|---|---|---|---|---|
| WT | 16 | 1.13667 | 0.68803 | 0.19862 | 0.47339 | 4 | 12 |
| Lyz2 | 15 | 0.98949 | 1.03401 | 0.29849 | 1.06917 | 3 | 12 |
| cKO COX-2 | 14 | 0.34428 | 0.31137 | 0.08636 | 0.09695 | 1 | 13 |

*Timepoint*

| | N | Mean | SD | SEM | Variance | Missing | NonMissing |
|---|---|---|---|---|---|---|---|
| 10 | 23 | 0.81043 | 0.90274 | 0.2071 | 0.81493 | 4 | 19 |
| 14 | 22 | 0.81064 | 0.68383 | 0.16118 | 0.46763 | 4 | 18 |

*Gender*

| | N | Mean | SD | SEM | Variance | Missing | NonMissing |
|---|---|---|---|---|---|---|---|
| Male | 23 | 0.61568 | 0.54389 | 0.12478 | 0.29582 | 4 | 19 |
| Female | 22 | 1.0162 | 0.96439 | 0.22731 | 0.93005 | 4 | 18 |

*Genotype*Timepoint*

| | | N | Mean | SD | SEM | Variance | Missing | NonMissing |
|---|---|---|---|---|---|---|---|---|
| WT | 10 | 9 | 1.03578 | 0.30665 | 0.12519 | 0.09404 | 3 | 6 |
| | 14 | 7 | 1.23756 | 0.96072 | 0.39221 | 0.92299 | 1 | 6 |
| Lyz2 | 10 | 6 | 1.36238 | 1.32876 | 0.54247 | 1.76561 | 0 | 6 |
| | 14 | 9 | 0.6166 | 0.50285 | 0.20529 | 0.25286 | 3 | 6 |
| cKO COX-2 | 10 | 8 | 0.14417 | 0.14753 | 0.05576 | 0.02177 | 1 | 7 |
| | 14 | 6 | 0.57775 | 0.29171 | 0.11909 | 0.0851 | 0 | 6 |

*Genotype*Gender*

| | | N | Mean | SD | SEM | Variance | Missing | NonMissing |
|---|---|---|---|---|---|---|---|---|
| WT | Male | 8 | 1.06372 | 0.41277 | 0.16851 | 0.17038 | 2 | 6 |
| | Female | 8 | 1.20962 | 0.92645 | 0.37822 | 0.85831 | 2 | 6 |
| Lyz2 | Male | 8 | 0.59728 | 0.65749 | 0.26842 | 0.43229 | 2 | 6 |
| | Female | 7 | 1.3817 | 1.24528 | 0.50838 | 1.55071 | 1 | 6 |
| cKO COX-2 | Male | 7 | 0.24742 | 0.16104 | 0.06087 | 0.02594 | 0 | 7 |
| | Female | 7 | 0.45729 | 0.41606 | 0.16985 | 0.1731 | 1 | 6 |

*Timepoint*Gender*

| | | N | Mean | SD | SEM | Variance | Missing | NonMissing |
|---|---|---|---|---|---|---|---|---|
| 10 | Male | 11 | 0.593 | 0.62652 | 0.19812 | 0.39253 | 1 | 10 |
| | Female | 12 | 1.05202 | 1.12572 | 0.37524 | 1.26725 | 3 | 9 |
| 14 | Male | 12 | 0.64089 | 0.47185 | 0.15728 | 0.22265 | 3 | 9 |
| | Female | 10 | 0.98039 | 0.84037 | 0.28012 | 0.70622 | 1 | 9 |

*Genotype*Timepoint*Gender*

| | | | N | Mean | SD | SEM | Variance | Missing | NonMissing |
|---|---|---|---|---|---|---|---|---|---|
| WT | 10 | Male | 4 | 1.04924 | 0.40854 | 0.23587 | 0.16691 | 1 | 3 |
| | | Female | 5 | 1.02233 | 0.26009 | 0.15016 | 0.06764 | 2 | 3 |
| | 14 | Male | 4 | 1.07821 | 0.50834 | 0.29349 | 0.25841 | 1 | 3 |
| | | Female | 3 | 1.39692 | 1.40459 | 0.81094 | 1.97288 | 0 | 3 |

| | | | | | | | | | |
|---|---|---|---|---|---|---|---|---|---|
| Lyz2 | 10 | Male | 3 | 0.72548 | 0.915 | 0.52828 | 0.83722 | 0 | 3 |
| | | Female | 3 | 1.99928 | 1.53618 | 0.88692 | 2.35986 | 0 | 3 |
| | 14 | Male | 5 | 0.46909 | 0.44068 | 0.25443 | 0.1942 | 2 | 3 |
| | | Female | 4 | 0.76411 | 0.61046 | 0.35245 | 0.37266 | 1 | 3 |
| cKO COX-2 | 10 | Male | 4 | 0.15146 | 0.15004 | 0.07502 | 0.02251 | 0 | 4 |
| | | Female | 4 | 0.13444 | 0.17686 | 0.10211 | 0.03128 | 1 | 3 |
| | 14 | Male | 3 | 0.37536 | 0.0327 | 0.01888 | 0.00107 | 0 | 3 |
| | | Female | 3 | 0.78013 | 0.29797 | 0.17203 | 0.08879 | 0 | 3 |

*ANOVA*

*Overall ANOVA*

| | DF | Sum of Squares | Mean Square | F Value | P Value |
|---|---|---|---|---|---|
| Genotype | 2 | 4.25241 | 2.1262 | 4.1627 | 0.02751 |
| Timepoint | 1 | 0.01218 | 0.01218 | 0.02385 | 0.87852 |
| Gender | 1 | 1.29069 | 1.29069 | 2.52692 | 0.12449 |
| Genotype*Timepoint | 2 | 2.38938 | 1.19469 | 2.33897 | 0.11717 |
| Genotype*Gender | 2 | 0.76514 | 0.38257 | 0.74899 | 0.48316 |
| Timepoint*Gender | 1 | 0.01141 | 0.01141 | 0.02233 | 0.8824 |
| Genotype*Timepoint*Gender | 2 | 0.94223 | 0.47111 | 0.92235 | 0.41069 |
| Model | 11 | 9.84902 | 0.89537 | 1.75295 | 0.11872 |
| Error | 25 | 12.7694 | 0.51078 | | |
| Corrected Total | 36 | 22.61842 | | | |

At the 0.05 level, the population means of **Genotype** are **significantly** different.
At the 0.05 level, the population means of **Timepoint** are **not significantly** different.
At the 0.05 level, the population means of **Gender** are **not significantly** different.
At the 0.05 level, the population means of **Genotype*Timepoint** are **not significantly** different.
At the 0.05 level, the population means of **Genotype*Gender** are **not significantly** different.
At the 0.05 level, the population means of **Timepoint*Gender** are **not significantly** different.
At the 0.05 level, the population means of **Genotype*Timepoint*Gender** are **not significantly** different.

*Means Comparisons  Tukey Test*

*Genotype*

| | MeanDiff | SEM | q Value | Prob | Alpha | Sig | LCL | UCL |
|---|---|---|---|---|---|---|---|---|
| WT Lyz2 | 0.14719 | 0.23983 | 0.8679 | 0.81402 | 0.05 | 0 | -0.45019 | 0.74456 |
| WT cKO COX-2 | 0.77632 | 0.23606 | 4.65097 | 0.00811 | 0.05 | 1 | 0.18836 | 1.36429 |
| Lyz2 cKO COX-2 | 0.62914 | 0.23606 | 3.76919 | 1 | 0.05 | 0 | 0.04117 | 1.21711 |

*Timepoint*

| | MeanDiff | SEM | q Value | Prob | Alpha | Sig | LCL | UCL |
|---|---|---|---|---|---|---|---|---|
| 10 14 | 0.0364 | 0.19377 | 0.26568 | 0.8525 | 0.05 | 0 | -0.36268 | 0.43549 |

*Gender*

| | MeanDiff | SEM | q Value | Prob | Alpha | Sig | LCL | UCL |
|---|---|---|---|---|---|---|---|---|
| Male Female | -0.37473 | 0.19377 | -2.7349 | 0.06453 | 0.05 | 0 | -0.77381 | 0.02435 |

*Genotype ’s*

| | Mean | Groups | |
|---|---|---|---|
| WT | 1.13667 | A | |
| Lyz2 | 0.98949 | A | B |
| cKO COX-2 | 0.34428 | | B |

Means that do not share a letter are significantly different.

*Timepoint ’s*

| | Mean | Groups |
|---|---|---|
| 14 | 0.81064 | A |
| 10 | 0.81043 | A |

Means that do not share a letter are significantly different.

*Gender ’s Gr*

| | Mean | Groups |
|---|---|---|
| Female | 1.0162 | A |
| Male | 0.61568 | A |

Means that do not share a letter are significantly different.

*Interactions ’*

| Genotype | Timepoint | Gender | Mean | Groups | |
|---|---|---|---|---|---|
| Lyz2 | 10 | Female | 1.99928 | A | |
| WT | 14 | Female | 1.39692 | A | B |
| WT | 14 | Male | 1.07821 | A | B |
| WT | 10 | Male | 1.04924 | A | B |
| WT | 10 | Female | 1.02233 | A | B |
| cKO COX-2 | 14 | Female | 0.78013 | A | B |
| Lyz2 | 14 | Female | 0.76411 | A | B |
| Lyz2 | 10 | Male | 0.72548 | A | B |
| Lyz2 | 14 | Male | 0.46909 | A | B |
| cKO COX-2 | 14 | Male | 0.37536 | A | B |
| cKO COX-2 | 10 | Male | 0.15146 | | B |
| cKO COX-2 | 10 | Female | 0.13444 | | B |

Means that do not share a letter are significantly different.

Sig equals 1 indicates that the difference of the means is significant at the 0.05 level. Sig equals 0 indicates that the difference of the means is not significant at the 0.05 level.

*ANOVAThreeWay (11/5/2024 15:14:0*

*Descriptive Statistics Genotype*

| | N | Mean | SD | SEM | Variance | Missing | NonMissing |
|---|---|---|---|---|---|---|---|
| WT | 42 | 28.93786 | 17.12701 | 2.64275 | 293.33442 | 0 | 42 |
| cKO COX-2 | 42 | 24.78095 | 12.84567 | 1.98213 | 165.01122 | 0 | 42 |
| Lyz2 | 45 | 32.49089 | 16.9583 | 2.52799 | 287.58386 | 0 | 45 |

*Timepoint*

| | N | Mean | SD | SEM | Variance | Missing | NonMissing |
|---|---|---|---|---|---|---|---|
| 7 | 24 | 11.2525 | 6.33045 | 1.2922 | 40.07456 | 0 | 24 |
| 10 | 29 | 13.71621 | 4.76585 | 0.885 | 22.7133 | 0 | 29 |
| 14 | 43 | 36.29023 | 9.98189 | 1.52222 | 99.63811 | 0 | 43 |
| 21 | 33 | 45.15061 | 7.44311 | 1.29568 | 55.39993 | 0 | 33 |

*Gender*

| | N | Mean | SD | SEM | Variance | Missing | NonMissing |
|---|---|---|---|---|---|---|---|
| Male | 55 | 25.55873 | 15.39806 | 2.07627 | 237.10035 | 0 | 55 |
| Female | 74 | 31.25068 | 16.09578 | 1.8711 | 259.07429 | 0 | 74 |

*Genotype*Timepoint*

| | | N | Mean | SD | SEM | Variance | Missing | NonMissing |
|---|---|---|---|---|---|---|---|---|
| WT | 7 | 8 | 9.2375 | 3.03504 | 1.07305 | 9.21145 | 0 | 8 |
| | 10 | 10 | 13.551 | 4.46298 | 1.41132 | 19.91821 | 0 | 10 |
| | 14 | 13 | 36.50615 | 7.34451 | 2.037 | 53.94186 | 0 | 13 |
| | 21 | 11 | 48.30909 | 8.89358 | 2.68152 | 79.09583 | 0 | 11 |
| cKO COX-2 | 7 | 7 | 8.63429 | 3.68608 | 1.39321 | 13.58716 | 0 | 7 |
| | 10 | 8 | 11.8325 | 6.27854 | 2.2198 | 39.42005 | 0 | 8 |
| | 14 | 15 | 27.894 | 6.4834 | 1.67401 | 42.03445 | 0 | 15 |
| | 21 | 12 | 38.94083 | 3.0424 | 0.87827 | 9.25621 | 0 | 12 |
| Lyz2 | 7 | 9 | 15.08 | 8.39194 | 2.79731 | 70.4246 | 0 | 9 |
| | 10 | 11 | 15.23636 | 3.55934 | 1.07318 | 12.66889 | 0 | 11 |
| | 14 | 15 | 44.49933 | 7.92879 | 2.0472 | 62.86565 | 0 | 15 |
| | 21 | 10 | 49.128 | 4.07367 | 1.28821 | 16.59477 | 0 | 10 |

*Genotype*Gender*

| | | N | Mean | SD | SEM | Variance | Missing | NonMissing |
|---|---|---|---|---|---|---|---|---|
| WT | Male | 21 | 24.49905 | 15.78524 | 3.44462 | 249.17386 | 0 | 21 |
| | Female | 21 | 33.37667 | 17.6291 | 3.84699 | 310.78533 | 0 | 21 |
| cKO COX-2 | Male | 22 | 24.27818 | 13.6081 | 2.90126 | 185.18049 | 0 | 22 |
| | Female | 20 | 25.334 | 12.27962 | 2.74581 | 150.78901 | 0 | 20 |
| Lyz2 | Male | 12 | 29.76083 | 18.23982 | 5.26538 | 332.69112 | 0 | 12 |
| | Female | 33 | 33.48364 | 16.65094 | 2.89856 | 277.25394 | 0 | 33 |

*Timepoint*Gender*

| | | N | Mean | SD | SEM | Variance | Missing | NonMissing |
|---|---|---|---|---|---|---|---|---|
| 7 | Male | 11 | 8.39909 | 3.24228 | 0.97758 | 10.51235 | 0 | 11 |
| | Female | 13 | 13.66692 | 7.36686 | 2.0432 | 54.27061 | 0 | 13 |
| 10 | Male | 13 | 12.56077 | 4.43875 | 1.23109 | 19.70252 | 0 | 13 |
| | Female | 16 | 14.655 | 4.95369 | 1.23842 | 24.53903 | 0 | 16 |
| 14 | Male | 16 | 31.9675 | 9.32762 | 2.33191 | 87.0045 | 0 | 16 |
| | Female | 27 | 38.85185 | 9.61486 | 1.85038 | 92.44561 | 0 | 27 |
| 21 | Male | 15 | 42.57133 | 7.18587 | 1.85538 | 51.63673 | 0 | 15 |
| | Female | 18 | 47.3 | 7.14116 | 1.68319 | 50.99622 | 0 | 18 |

*Genotype*Timepoint*Gender*

| | | | N | Mean | SD | SEM | Variance | Missing | NonMissing |
|---|---|---|---|---|---|---|---|---|---|
| WT | 7 | Male | 4 | 6.7175 | 1.91587 | 0.95794 | 3.67056 | 0 | 4 |
| | | Female | 4 | 11.7575 | 0.94256 | 0.47128 | 0.88843 | 0 | 4 |
| | 10 | Male | 6 | 12.325 | 4.99027 | 2.03727 | 24.90275 | 0 | 6 |
| | | Female | 4 | 15.39 | 3.27638 | 1.63819 | 10.73467 | 0 | 4 |
| | 14 | Male | 7 | 33.35286 | 5.46529 | 2.06569 | 29.86942 | 0 | 7 |
| | | Female | 6 | 40.185 | 7.96592 | 3.25207 | 63.45587 | 0 | 6 |
| | 21 | Male | 4 | 45.0475 | 9.61027 | 4.80513 | 92.35723 | 0 | 4 |
| | | Female | 7 | 50.17286 | 8.63152 | 3.26241 | 74.50322 | 0 | 7 |
| cKO COX-2 | 7 | Male | 4 | 9.565 | 4.70396 | 2.35198 | 22.12723 | 0 | 4 |
| | | Female | 3 | 7.39333 | 1.87836 | 1.08447 | 3.52823 | 0 | 3 |
| | 10 | Male | 4 | 9.9425 | 2.79031 | 1.39515 | 7.78582 | 0 | 4 |
| | | Female | 4 | 13.7225 | 8.6411 | 4.32055 | 74.66869 | 0 | 4 |
| | 14 | Male | 6 | 24.95167 | 9.63189 | 3.9322 | 92.77322 | 0 | 6 |
| | | Female | 9 | 29.85556 | 2.18068 | 0.72689 | 4.75538 | 0 | 9 |
| | 21 | Male | 8 | 38.2975 | 3.21104 | 1.13527 | 10.31076 | 0 | 8 |
| | | Female | 4 | 40.2275 | 2.56319 | 1.2816 | 6.56996 | 0 | 4 |
| Lyz2 | 7 | Male | 3 | 9.08667 | 2.18672 | 1.2625 | 4.78173 | 0 | 3 |
| | | Female | 6 | 18.07667 | 8.85656 | 3.61567 | 78.43863 | 0 | 6 |
| | 10 | Male | 3 | 16.52333 | 2.6171 | 1.51099 | 6.84923 | 0 | 3 |
| | | Female | 8 | 14.75375 | 3.89428 | 1.37684 | 15.16546 | 0 | 8 |
| | 14 | Male | 3 | 42.76667 | 1.29477 | 0.74754 | 1.67643 | 0 | 3 |
| | | Female | 12 | 44.9325 | 8.87032 | 2.56064 | 78.68257 | 0 | 12 |
| | 21 | Male | 3 | 50.66667 | 1.79068 | 1.03385 | 3.20653 | 0 | 3 |
| | | Female | 7 | 48.46857 | 4.70449 | 1.77813 | 22.13225 | 0 | 7 |

*ANOVA*

*Overall ANOVA*

| | DF | Sum of Squares | Mean Square | F Value | P Value |
|---|---|---|---|---|---|
| Genotype | 2 | 1434.5815 | 717.29075 | 19.63767 | <0.0001 |
| Timepoint | 3 | 23451.03837 | 7817.01279 | 214.01069 | <0.0001 |
| Gender | 1 | 244.69766 | 244.69766 | 6.69922 | 0.01101 |
| Genotype*Timepoint | 6 | 563.82732 | 93.97122 | 2.5727 | 0.02297 |
| Genotype*Gender | 2 | 59.88465 | 29.94232 | 0.81975 | 0.44334 |
| Timepoint*Gender | 3 | 54.01033 | 18.00344 | 0.49289 | 0.68798 |
| Genotype*Timepoint*Gender | 6 | 186.51293 | 31.08549 | 0.85104 | 0.5336 |
| Model | 23 | 28902.76211 | 1256.64183 | 34.40378 | <0.0001 |
| Error | 105 | 3835.25856 | 36.52627 | | |
| Corrected Total | 128 | 32738.02066 | | | |

At the 0.05 level, the population means of **Genotype** are **significantly** different.
At the 0.05 level, the population means of **Timepoint** are **significantly** different.
At the 0.05 level, the population means of **Gender** are **significantly** different.
At the 0.05 level, the population means of **Genotype*Timepoint** are **significantly** different. At the 0.05 level, the population means of **Genotype*Gender** are **not significantly** different. At the 0.05 level, the population means of **Timepoint*Gender** are **not significantly** different.
At the 0.05 level, the population means of **Genotype*Timepoint*Gender** are **not significantly** different.

*Means Comparisons Tukey Test*

*Genotype*

| | MeanDiff | SEM | q Value | Prob | Alpha | Sig | LCL | UCL |
|---|---|---|---|---|---|---|---|---|
| WT cKO COX-2 | 5.12408 | 1.24844 | 5.80446 | 2.35566E-4 | 0.05 | 1 | 2.15603 | 8.09214 |
| WT Lyz2 | -3.79083 | 1.26967 | -4.22237 | 0.00978 | 0.05 | 1 | -6.80935 | -0.7723 |
| cKO COX-2 Lyz2 | -8.91491 | 1.29091 | -9.76642 | <0.0001 | 0.05 | 1 | -11.98393 | -5.84589 |

*Timepoint*

| | MeanDiff | SEM | q Value | Prob | Alpha | Sig | LCL | UCL |
|---|---|---|---|---|---|---|---|---|
| 7 10 | -3.3434 | 1.56306 | -3.02502 | 0.14763 | 0.05 | 0 | -7.424 | 0.7372 |
| 7 14 | -25.5746 | 1.46176 | -24.74281 | <0.0001 | 0.05 | 1 | -29.39074 | -21.75846 |
| 7 21 | -35.04732 | 1.52807 | -32.43594 | <0.0001 | 0.05 | 1 | -39.03659 | -31.05806 |
| 10 14 | -22.23119 | 1.40167 | -22.43015 | <0.0001 | 0.05 | 1 | -25.89047 | -18.57192 |
| 10 21 | -31.70392 | 1.4707 | -30.48632 | <0.0001 | 0.05 | 1 | -35.5434 | -27.86444 |
| 14 21 | -9.47272 | 1.36255 | -9.83194 | <0.0001 | 0.05 | 1 | -13.02986 | -5.91559 |

*Gender*

| | MeanDiff | SEM | q Value | Prob | Alpha | Sig | LCL | UCL |
|---|---|---|---|---|---|---|---|---|
| Male Female | -2.97441 | 1.03678 | -4.05721 | 0.00498 | 0.05 | 1 | -5.03016 | -0.91866 |

*Genotype 's*

| | Mean | Groups | | |
|---|---|---|---|---|
| Lyz2 | 32.49089 | A | | |
| WT | 28.93786 | | B | |
| cKO COX-2 | 24.78095 | | | C |

Means that do not share a letter are significantly different.

*Timepoint 's*

| | Mean | Groups | | |
|---|---|---|---|---|
| 21 | 45.15061 | A | | |
| 14 | 36.29023 | | B | |
| 10 | 13.71621 | | | C |
| 7 | 11.2525 | | | C |

Means that do not share a letter are significantly different.

*Gender 's Gr*

| | Mean | Groups | |
|---|---|---|---|
| Female | 31.25068 | A | |
| Male | 25.55873 | | B |

Means that do not share a letter are significantly different.

*Interactions '*

| Genotype | Timepoint | Gender | Mean | Groups | | | | | | | |
|---|---|---|---|---|---|---|---|---|---|---|---|
| Lyz2 | 21 | Male | 50.66667 | A | B | | | | | | |
| WT | 21 | Female | 50.17286 | A | | | | | | | |
| Lyz2 | 21 | Female | 48.46857 | A | B | | | | | | |
| WT | 21 | Male | 45.0475 | A | B | C | | | | | |
| Lyz2 | 14 | Female | 44.9325 | A | B | | | | | | |
| Lyz2 | 14 | Male | 42.76667 | A | B | C | D | | | | |
| cKO COX-2 | 21 | Female | 40.2275 | A | B | C | D | | | | |
| WT | 14 | Female | 40.185 | A | B | C | D | | | | |
| cKO COX-2 | 21 | Male | 38.2975 | | B | C | D | | | | |
| WT | 14 | Male | 33.35286 | | | C | D | E | | | |
| cKO COX-2 | 14 | Female | 29.85556 | | | | D | E | F | | |
| cKO COX-2 | 14 | Male | 24.95167 | | | | | E | F | G | |
| Lyz2 | 7 | Female | 18.07667 | | | | | | | G | H |
| Lyz2 | 10 | Male | 16.52333 | | | | | | F | G | H |
| WT | 10 | Female | 15.39 | | | | | | | G | H |
| Lyz2 | 10 | Female | 14.75375 | | | | | | | G | H |
| cKO COX-2 | 10 | Female | 13.7225 | | | | | | | G | H |
| WT | 10 | Male | 12.325 | | | | | | | | H |
| WT | 7 | Female | 11.7575 | | | | | | | | H |
| cKO COX-2 | 10 | Male | 9.9425 | | | | | | | | H |
| cKO COX-2 | 7 | Male | 9.565 | | | | | | | | H |
| Lyz2 | 7 | Male | 9.08667 | | | | | | | | H |
| cKO COX-2 | 7 | Female | 7.39333 | | | | | | | | H |
| WT | 7 | Male | 6.7175 | | | | | | | | H |

Means that do not share a letter are significantly different.

Sig equals 1 indicates that the difference of the means is significant at the 0.05 level. Sig equals 0 indicates that the difference of the means is not significant at the 0.05 level.

*ANOVAThreeWay (11/5/2024 18:46:0*

*Descriptive Statistics Genotype*

| | N | Mean | SD | SEM | Variance | Missing | NonMissing |
|---|---|---|---|---|---|---|---|
| WT | 16 | 1.03969 | 0.31783 | 0.09175 | 0.10101 | 4 | 12 |
| Lyz2 | 15 | 1.12212 | 1.12568 | 0.32496 | 1.26716 | 3 | 12 |
| cKO COX-2 | 14 | 0.62135 | 0.54516 | 0.1512 | 0.2972 | 1 | 13 |

*Timepoint*

| | N | Mean | SD | SEM | Variance | Missing | NonMissing |
|---|---|---|---|---|---|---|---|
| 10 | 23 | 1.03932 | 0.891 | 0.20441 | 0.79387 | 4 | 19 |
| 14 | 22 | 0.7929 | 0.57327 | 0.13512 | 0.32864 | 4 | 18 |

*Gender*

| | N | Mean | SD | SEM | Variance | Missing | NonMissing |
|---|---|---|---|---|---|---|---|
| Male | 23 | 0.98373 | 0.95747 | 0.21966 | 0.91676 | 4 | 19 |
| Female | 22 | 0.85158 | 0.47122 | 0.11107 | 0.22205 | 4 | 18 |

*Genotype*Timepoint*

| | | N | Mean | SD | SEM | Variance | Missing | NonMissing |
|---|---|---|---|---|---|---|---|---|
| WT | 10 | 9 | 1.01408 | 0.17717 | 0.07233 | 0.03139 | 3 | 6 |
| | 14 | 7 | 1.06529 | 0.43505 | 0.17761 | 0.18927 | 1 | 6 |
| Lyz2 | 10 | 6 | 1.29978 | 1.53281 | 0.62577 | 2.3495 | 0 | 6 |
| | 14 | 9 | 0.94446 | 0.60207 | 0.24579 | 0.36249 | 3 | 6 |
| cKO COX-2 | 10 | 8 | 0.8377 | 0.5307 | 0.20059 | 0.28164 | 1 | 7 |
| | 14 | 6 | 0.36895 | 0.48304 | 0.1972 | 0.23332 | 0 | 6 |

*Genotype*Gender*

| | | N | Mean | SD | SEM | Variance | Missing | NonMissing |
|---|---|---|---|---|---|---|---|---|
| WT | Male | 8 | 1.02707 | 0.24831 | 0.10137 | 0.06166 | 2 | 6 |
| | Female | 8 | 1.05231 | 0.40024 | 0.1634 | 0.16019 | 2 | 6 |
| Lyz2 | Male | 8 | 1.30474 | 1.57495 | 0.64297 | 2.48047 | 2 | 6 |
| | Female | 7 | 0.9395 | 0.47669 | 0.19461 | 0.22724 | 1 | 6 |
| cKO COX-2 | Male | 7 | 0.67143 | 0.64274 | 0.24293 | 0.41312 | 0 | 7 |
| | Female | 7 | 0.56293 | 0.45817 | 0.18705 | 0.20992 | 1 | 6 |

*Timepoint*Gender*

| | | N | Mean | SD | SEM | Variance | Missing | NonMissing |
|---|---|---|---|---|---|---|---|---|
| 10 | Male | 11 | 1.14219 | 1.21035 | 0.38275 | 1.46494 | 1 | 10 |
| | Female | 12 | 0.92502 | 0.33201 | 0.11067 | 0.11023 | 3 | 9 |
| 14 | Male | 12 | 0.80767 | 0.59024 | 0.19675 | 0.34838 | 3 | 9 |
| | Female | 10 | 0.77814 | 0.59117 | 0.19706 | 0.34948 | 1 | 9 |

*Genotype*Timepoint*Gender*

| | | | N | Mean | SD | SEM | Variance | Missing | NonMissing |
|---|---|---|---|---|---|---|---|---|---|
| WT | 10 | Male | 4 | 1.02171 | 0.2446 | 0.14122 | 0.05983 | 1 | 3 |
| | | Female | 5 | 1.00646 | 0.13591 | 0.07847 | 0.01847 | 2 | 3 |
| | 14 | Male | 4 | 1.03243 | 0.30697 | 0.17723 | 0.09423 | 1 | 3 |
| | | Female | 3 | 1.09816 | 0.61294 | 0.35388 | 0.3757 | 0 | 3 |

| | | | | | | | | | |
|---|---|---|---|---|---|---|---|---|---|
| Lyz2 | 10 | Male | 3 | 1.71863 | 2.26272 | 1.30638 | 5.11988 | 0 | 3 |
| | | Female | 3 | 0.88093 | 0.47704 | 0.27542 | 0.22757 | 0 | 3 |
| | 14 | Male | 5 | 0.89085 | 0.75324 | 0.43488 | 0.56737 | 2 | 3 |
| | | Female | 4 | 0.99806 | 0.57466 | 0.33178 | 0.33023 | 1 | 3 |
| cKO COX-2 | 10 | Male | 4 | 0.80022 | 0.66254 | 0.33127 | 0.43896 | 0 | 4 |
| | | Female | 4 | 0.88767 | 0.42419 | 0.24491 | 0.17994 | 1 | 3 |
| | 14 | Male | 3 | 0.49972 | 0.70959 | 0.40968 | 0.50352 | 0 | 3 |
| | | Female | 3 | 0.23819 | 0.16879 | 0.09745 | 0.02849 | 0 | 3 |

*ANOVA*

*Overall ANOVA*

| | DF | Sum of Squares | Mean Square | F Value | P Value |
|---|---|---|---|---|---|
| Genotype | 2 | 1.91993 | 0.95997 | 1.46988 | 0.24915 |
| Timepoint | 1 | 0.61992 | 0.61992 | 0.94921 | 0.33925 |
| Gender | 1 | 0.18625 | 0.18625 | 0.28518 | 0.59805 |
| Genotype*Timepoint | 2 | 0.46554 | 0.23277 | 0.35641 | 0.70368 |
| Genotype*Gender | 2 | 0.24308 | 0.12154 | 0.1861 | 0.83133 |
| Timepoint*Gender | 1 | 0.11699 | 0.11699 | 0.17913 | 0.67574 |
| Genotype*Timepoint*Gender | 2 | 0.6675 | 0.33375 | 0.51103 | 0.60601 |
| Model | 11 | 4.11047 | 0.37368 | 0.57217 | 0.83292 |
| Error | 25 | 16.32733 | 0.65309 | | |
| Corrected Total | 36 | 20.4378 | | | |

At the 0.05 level, the population means of **Genotype** are **not significantly** different.

At the 0.05 level, the population means of **Timepoint** are **not significantly** different.

At the 0.05 level, the population means of **Gender** are **not significantly** different.

At the 0.05 level, the population means of **Genotype*Timepoint** are **not significantly** different.

At the 0.05 level, the population means of **Genotype*Gender** are **not significantly** different.

At the 0.05 level, the population means of **Timepoint*Gender** are **not significantly** different.

At the 0.05 level, the population means of **Genotype*Timepoint*Gender** are **not significantly** different.

*Means Comparisons Tukey Test*

*Genotype*

| | MeanDiff | SEM | q Value | Prob | Alpha | Sig | LCL | UCL |
|---|---|---|---|---|---|---|---|---|
| WT Lyz2 | -0.08243 | 0.27119 | -0.42986 | 0.95045 | 0.05 | 0 | -0.75792 | 0.59306 |
| WT cKO COX-2 | 0.43324 | 0.26692 | 2.29539 | 0.2548 | 0.05 | 0 | -0.23161 | 1.09809 |
| Lyz2 cKO COX-2 | 0.51567 | 0.26692 | 2.73213 | 0.15069 | 0.05 | 0 | -0.14918 | 1.18053 |

*Timepoint*

| | MeanDiff | SEM | q Value | Prob | Alpha | Sig | LCL | UCL |
|---|---|---|---|---|---|---|---|---|
| 10 14 | 0.2597 | 0.21911 | 1.67621 | 0.24706 | 0.05 | 0 | -0.19157 | 0.71097 |

*Gender*

| | MeanDiff | SEM | q Value | Prob | Alpha | Sig | LCL | UCL |
|---|---|---|---|---|---|---|---|---|
| Male Female | 0.14235 | 0.21911 | 0.91876 | 0.52184 | 0.05 | 0 | -0.30892 | 0.59362 |

*Genotype 's*

| | Mean | Groups |
|---|---|---|
| Lyz2 | 1.12212 | A |
| WT | 1.03969 | A |
| cKO COX-2 | 0.62135 | A |

Means that do not share a letter are significantly different.

*Timepoint 's*

| | Mean | Groups |
|---|---|---|
| 10 | 1.03932 | A |
| 14 | 0.7929 | A |

Means that do not share a letter are significantly different.

*Gender 's Gr*

| | Mean | Groups |
|---|---|---|
| Male | 0.98373 | A |
| Female | 0.85158 | A |

Means that do not share a letter are significantly different.

*Interactions '*

| Genotype | Timepoint | Gender | Mean | Groups |
|---|---|---|---|---|
| Lyz2 | 10 | Male | 1.71863 | A |
| WT | 14 | Female | 1.09816 | A |
| WT | 14 | Male | 1.03243 | A |
| WT | 10 | Male | 1.02171 | A |
| WT | 10 | Female | 1.00646 | A |
| Lyz2 | 14 | Female | 0.99806 | A |
| Lyz2 | 14 | Male | 0.89085 | A |
| cKO COX-2 | 10 | Female | 0.88767 | A |
| Lyz2 | 10 | Female | 0.88093 | A |
| cKO COX-2 | 10 | Male | 0.80022 | A |
| cKO COX-2 | 14 | Male | 0.49972 | A |
| cKO COX-2 | 14 | Female | 0.23819 | A |

Means that do not share a letter are significantly different.

Sig equals 1 indicates that the difference of the means is significant at the 0.05 level. Sig equals 0 indicates that the difference of the means is not significant at the 0.05 level.

*ANOVAThreeWay (11/5/2024 15:38:5*

*Descriptive Statistics Genotype*

| | N | Mean | SD | SEM | Variance | Missing | NonMissing |
|---|---|---|---|---|---|---|---|
| WT | 19 | 42.80354 | 17.49514 | 4.01366 | 306.08002 | 0 | 19 |
| cKO COX-2 | 24 | 32.07803 | 16.05245 | 3.27669 | 257.68116 | 0 | 24 |
| Lyz2 | 27 | 42.5529 | 22.1812 | 4.26877 | 492.00565 | 0 | 27 |

*Timepoint*

| | N | Mean | SD | SEM | Variance | Missing | NonMissing |
|---|---|---|---|---|---|---|---|
| 10 | 21 | 14.61906 | 7.91383 | 1.72694 | 62.6287 | 0 | 21 |
| 14 | 23 | 48.4359 | 12.73266 | 2.65494 | 162.12064 | 0 | 23 |
| 21 | 26 | 50.4247 | 11.40203 | 2.23612 | 130.00619 | 0 | 26 |

*Gender*

| | N | Mean | SD | SEM | Variance | Missing | NonMissing |
|---|---|---|---|---|---|---|---|
| Male | 32 | 39.00384 | 17.78164 | 3.14338 | 316.18677 | 0 | 32 |
| Female | 38 | 39.05119 | 20.9194 | 3.39357 | 437.62124 | 0 | 38 |

*Genotype*Timepoint*

| | | N | Mean | SD | SEM | Variance | Missing | NonMissing |
|---|---|---|---|---|---|---|---|---|
| WT | 10 | 6 | 21.21533 | 10.80285 | 4.41024 | 116.70155 | 0 | 6 |
| | 14 | 6 | 52.12268 | 9.12287 | 3.7244 | 83.22676 | 0 | 6 |
| | 21 | 7 | 53.31989 | 8.34927 | 3.15573 | 69.71026 | 0 | 7 |
| cKO COX-2 | 10 | 6 | 9.6568 | 4.40171 | 1.79699 | 19.37505 | 0 | 6 |
| | 14 | 8 | 36.9102 | 12.46129 | 4.40573 | 155.28363 | 0 | 8 |
| | 21 | 10 | 41.66504 | 8.30415 | 2.626 | 68.95891 | 0 | 10 |
| Lyz2 | 10 | 9 | 13.52971 | 4.36895 | 1.45632 | 19.08773 | 0 | 9 |
| | 14 | 9 | 56.22312 | 6.83263 | 2.27754 | 46.68488 | 0 | 9 |
| | 21 | 9 | 57.90586 | 10.4974 | 3.49913 | 110.19534 | 0 | 9 |

*Genotype*Gender*

| | | N | Mean | SD | SEM | Variance | Missing | NonMissing |
|---|---|---|---|---|---|---|---|---|
| WT | Male | 9 | 43.08247 | 17.63011 | 5.8767 | 310.82087 | 0 | 9 |
| | Female | 10 | 42.55251 | 18.32285 | 5.7942 | 335.72701 | 0 | 10 |
| cKO COX-2 | Male | 14 | 35.33547 | 16.27843 | 4.35059 | 264.98715 | 0 | 14 |
| | Female | 10 | 27.51762 | 15.36702 | 4.85948 | 236.14528 | 0 | 10 |
| Lyz2 | Male | 9 | 40.63157 | 20.94168 | 6.98056 | 438.55384 | 0 | 9 |
| | Female | 18 | 43.51356 | 23.306 | 5.49328 | 543.16947 | 0 | 18 |

*Timepoint*Gender*

| | | N | Mean | SD | SEM | Variance | Missing | NonMissing |
|---|---|---|---|---|---|---|---|---|
| 10 | Male | 9 | 16.17597 | 9.62897 | 3.20966 | 92.71701 | 0 | 9 |
| | Female | 12 | 13.45138 | 6.55508 | 1.89229 | 42.96914 | 0 | 12 |
| 14 | Male | 10 | 47.00212 | 12.07806 | 3.81942 | 145.87945 | 0 | 10 |
| | Female | 13 | 49.53882 | 13.59341 | 3.77013 | 184.78069 | 0 | 13 |
| 21 | Male | 13 | 48.65523 | 10.02691 | 2.78096 | 100.53885 | 0 | 13 |
| | Female | 13 | 52.19418 | 12.78763 | 3.54665 | 163.52348 | 0 | 13 |

*Genotype*Timepoint*Gender*

| | | | N | Mean | SD | SEM | Variance | Missing | NonMissing |
|---|---|---|---|---|---|---|---|---|---|
| WT | 10 | Male | 3 | 23.94287 | 14.64859 | 8.45737 | 214.58108 | 0 | 3 |
| | | Female | 3 | 18.4878 | 7.40638 | 4.27608 | 54.85447 | 0 | 3 |
| | 14 | Male | 3 | 47.14433 | 10.54967 | 6.09085 | 111.29552 | 0 | 3 |
| | | Female | 3 | 57.10103 | 4.73492 | 2.73371 | 22.41949 | 0 | 3 |
| | 21 | Male | 3 | 58.1602 | 1.47148 | 0.84956 | 2.16526 | 0 | 3 |
| | | Female | 4 | 49.68965 | 9.84768 | 4.92384 | 96.97689 | 0 | 4 |
| cKO COX-2 | 10 | Male | 3 | 10.75403 | 1.90069 | 1.09736 | 3.61263 | 0 | 3 |
| | | Female | 3 | 8.55957 | 6.41975 | 3.70645 | 41.21324 | 0 | 3 |
| | 14 | Male | 4 | 41.68955 | 13.90964 | 6.95482 | 193.47808 | 0 | 4 |
| | | Female | 4 | 32.13085 | 10.38932 | 5.19466 | 107.93789 | 0 | 4 |
| | 21 | Male | 7 | 42.23947 | 9.56799 | 3.61636 | 91.54639 | 0 | 7 |
| | | Female | 3 | 40.3247 | 5.64148 | 3.25711 | 31.82627 | 0 | 3 |
| Lyz2 | 10 | Male | 3 | 13.831 | 3.13734 | 1.81134 | 9.84288 | 0 | 3 |
| | | Female | 6 | 13.37907 | 5.1499 | 2.10244 | 26.52152 | 0 | 6 |
| | 14 | Male | 3 | 53.94333 | 11.23375 | 6.48581 | 126.19708 | 0 | 3 |
| | | Female | 6 | 57.36302 | 4.42033 | 1.80459 | 19.53928 | 0 | 6 |
| | 21 | Male | 3 | 54.12037 | 1.42953 | 0.82534 | 2.04355 | 0 | 3 |
| | | Female | 6 | 59.7986 | 12.7514 | 5.20574 | 162.59819 | 0 | 6 |

*ANOVA*

*Overall ANOVA*

| | DF | Sum of Squares | Mean Square | F Value | P Value |
|---|---|---|---|---|---|
| Genotype | 2 | 2428.62197 | 1214.31099 | 15.67687 | <0.0001 |
| Timepoint | 2 | 16486.64544 | 8243.32272 | 106.4221 | <0.0001 |
| Gender | 1 | 15.97695 | 15.97695 | 0.20626 | 0.6516 |
| Genotype*Timepoint | 4 | 513.99535 | 128.49884 | 1.65893 | 0.17363 |
| Genotype*Gender | 2 | 159.85605 | 79.92802 | 1.03188 | 0.36352 |
| Timepoint*Gender | 2 | 43.92705 | 21.96353 | 0.28355 | 0.75426 |
| Genotype*Timepoint*Gender | 4 | 385.96131 | 96.49033 | 1.2457 | 0.30323 |
| Model | 17 | 21965.95986 | 1292.11529 | 16.68133 | <0.0001 |
| Error | 52 | 4027.85482 | 77.45875 | | |
| Corrected Total | 69 | 25993.81468 | | | |

At the 0.05 level, the population means of **Genotype** are **significantly** different.
At the 0.05 level, the population means of **Timepoint** are **significantly** different.
At the 0.05 level, the population means of **Gender** are **not significantly** different.
At the 0.05 level, the population means of **Genotype*Timepoint** are **not significantly** different. At the 0.05 level, the population means of **Genotype*Gender** are **not significantly** different.
At the 0.05 level, the population means of **Timepoint*Gender** are **not significantly** different.
At the 0.05 level, the population means of **Genotype*Timepoint*Gender** are **not significantly** different.

*Means Comparisons Tukey Test*

*Genotype*

| | MeanDiff | SEM | q Value | Prob | Alpha | Sig | LCL | UCL |
|---|---|---|---|---|---|---|---|---|
| WT cKO COX-2 | 13.13795 | 2.38524 | 7.78951 | <0.0001 | 0.05 | 1 | 7.38342 | 18.89248 |
| WT Lyz2 | 0.34842 | 2.33689 | 0.21085 | 0.98782 | 0.05 | 0 | -5.28946 | 5.98629 |
| cKO COX-2 Lyz2 | -12.78954 | 2.24129 | -8.06995 | <0.0001 | 0.05 | 1 | -18.19678 | -7.38229 |

*Timepoint*

| | MeanDiff | SEM | q Value | Prob | Alpha | Sig | LCL | UCL |
|---|---|---|---|---|---|---|---|---|
| 10 14 | -33.40296 | 2.36522 | -19.97236 | <0.0001 | 0.05 | 1 | -39.10918 | -27.69675 |
| 10 21 | -35.89644 | 2.32873 | -21.79952 | <0.0001 | 0.05 | 1 | -41.51464 | -30.27825 |
| 14 21 | -2.49348 | 2.27081 | -1.55288 | 0.51957 | 0.05 | 0 | -7.97195 | 2.98499 |

*Gender*

| | MeanDiff | SEM | q Value | Prob | Alpha | Sig | LCL | UCL |
|---|---|---|---|---|---|---|---|---|
| Male Female | 0.99899 | 1.89583 | 0.7452 | 0.60048 | 0.05 | 0 | -2.80529 | 4.80326 |

*Genotype 's*

| | Mean | Groups | |
|---|---|---|---|
| WT | 42.80354 | A | |
| Lyz2 | 42.5529 | A | |
| cKO COX-2 | 32.07803 | | B |

Means that do not share a letter are significantly different.

*Timepoint 's*

| | Mean | Groups | |
|---|---|---|---|
| 21 | 50.4247 | A | |
| 14 | 48.4359 | A | |
| 10 | 14.61906 | | B |

Means that do not share a letter are significantly different.

*Gender 's Gr*

| | Mean | Groups |
|---|---|---|
| Female | 39.05119 | A |
| Male | 39.00384 | A |

Means that do not share a letter are significantly different.

*Interactions '*

| Genotype | Timepoint | Gender | Mean | Groups | | | | | | |
|---|---|---|---|---|---|---|---|---|---|---|
| Lyz2 | 21 | Female | 59.7986 | A | | | | | | |
| WT | 21 | Male | 58.1602 | A | B | | | | | |
| Lyz2 | 14 | Female | 57.36302 | A | B | | | | | |
| WT | 14 | Female | 57.10103 | A | B | | | | | |
| Lyz2 | 21 | Male | 54.12037 | A | B | | | | | |
| Lyz2 | 14 | Male | 53.94333 | A | B | | | | | |
| WT | 21 | Female | 49.68965 | A | B | C | | | | |
| WT | 14 | Male | 47.14433 | A | B | C | | | | |
| cKO COX-2 | 21 | Male | 42.23947 | | B | C | D | | | |
| cKO COX-2 | 14 | Male | 41.68955 | | B | C | D | | | |
| cKO COX-2 | 21 | Female | 40.3247 | A | B | C | D | E | | |
| cKO COX-2 | 14 | Female | 32.13085 | | | C | D | E | F | |
| WT | 10 | Male | 23.94287 | | | | D | E | F | G |
| WT | 10 | Female | 18.4878 | | | | | E | F | G |
| Lyz2 | 10 | Male | 13.831 | | | | | | F | G |
| Lyz2 | 10 | Female | 13.37907 | | | | | | | G |
| cKO COX-2 | 10 | Male | 10.75403 | | | | | | | G |
| cKO COX-2 | 10 | Female | 8.55957 | | | | | | | G |

Means that do not share a letter are significantly different.

Sig equals 1 indicates that the difference of the means is significant at the 0.05 level. Sig equals 0 indicates that the difference of the means is not significant at the 0.05 level.

## S7-D. Osteopontin (*Spp1*) mRNA Levels (RTqPCR)

*ANOVAThreeWay (11/5/2024 18:22:4*

*Descriptive Statistics Genotype*

| | N | Mean | SD | SEM | Variance | Missing | NonMissing |
|---|---|---|---|---|---|---|---|
| WT | 12 | 1.09593 | 0.47357 | 0.13671 | 0.22427 | 0 | 12 |
| Lyz2 | 12 | 1.34636 | 0.80997 | 0.23382 | 0.65606 | 0 | 12 |
| cKO COX-2 | 12 | 0.30291 | 0.18996 | 0.05484 | 0.03608 | 0 | 12 |

*Timepoint*

| | N | Mean | SD | SEM | Variance | Missing | NonMissing |
|---|---|---|---|---|---|---|---|
| 10 | 18 | 0.7496 | 0.56195 | 0.13245 | 0.31579 | 0 | 18 |
| 14 | 18 | 1.08054 | 0.79882 | 0.18828 | 0.63811 | 0 | 18 |

*Gender*

| | N | Mean | SD | SEM | Variance | Missing | NonMissing |
|---|---|---|---|---|---|---|---|
| Male | 18 | 0.80189 | 0.50945 | 0.12008 | 0.25954 | 0 | 18 |
| Female | 18 | 1.02825 | 0.8516 | 0.20072 | 0.72521 | 0 | 18 |

*Genotype*Timepoint*

| | | N | Mean | SD | SEM | Variance | Missing | NonMissing |
|---|---|---|---|---|---|---|---|---|
| WT | 10 | 6 | 1.07441 | 0.46155 | 0.18843 | 0.21303 | 0 | 6 |
| | 14 | 6 | 1.11745 | 0.52843 | 0.21573 | 0.27924 | 0 | 6 |
| Lyz2 | 10 | 6 | 0.87807 | 0.65814 | 0.26869 | 0.43315 | 0 | 6 |
| | 14 | 6 | 1.81466 | 0.69559 | 0.28397 | 0.48385 | 0 | 6 |
| cKO COX-2 | 10 | 6 | 0.29631 | 0.18583 | 0.07587 | 0.03453 | 0 | 6 |
| | 14 | 6 | 0.3095 | 0.21153 | 0.08636 | 0.04475 | 0 | 6 |

*Genotype*Gender*

| | | N | Mean | SD | SEM | Variance | Missing | NonMissing |
|---|---|---|---|---|---|---|---|---|
| WT | Male | 6 | 1.04042 | 0.33175 | 0.13544 | 0.11006 | 0 | 6 |
| | Female | 6 | 1.15144 | 0.61313 | 0.25031 | 0.37593 | 0 | 6 |
| Lyz2 | Male | 6 | 0.99028 | 0.66174 | 0.27015 | 0.4379 | 0 | 6 |
| | Female | 6 | 1.70244 | 0.83733 | 0.34184 | 0.70112 | 0 | 6 |
| cKO COX-2 | Male | 6 | 0.37496 | 0.07003 | 0.02859 | 0.0049 | 0 | 6 |
| | Female | 6 | 0.23086 | 0.24904 | 0.10167 | 0.06202 | 0 | 6 |

*Timepoint*Gender*

| | | N | Mean | SD | SEM | Variance | Missing | NonMissing |
|---|---|---|---|---|---|---|---|---|
| 10 | Male | 9 | 0.69787 | 0.45096 | 0.15032 | 0.20337 | 0 | 9 |
| | Female | 9 | 0.80133 | 0.67946 | 0.22649 | 0.46166 | 0 | 9 |
| 14 | Male | 9 | 0.90591 | 0.56905 | 0.18968 | 0.32382 | 0 | 9 |
| | Female | 9 | 1.25516 | 0.98161 | 0.3272 | 0.96356 | 0 | 9 |

*Genotype*Timepoint*Gender*

| | | | N | Mean | SD | SEM | Variance | Missing | NonMissing |
|---|---|---|---|---|---|---|---|---|---|
| WT | 10 | Male | 3 | 1.0287 | 0.29966 | 0.17301 | 0.08979 | 0 | 3 |
| | | Female | 3 | 1.12013 | 0.66069 | 0.38145 | 0.43652 | 0 | 3 |
| | 14 | Male | 3 | 1.05215 | 0.43005 | 0.24829 | 0.18494 | 0 | 3 |
| | | Female | 3 | 1.18275 | 0.70737 | 0.4084 | 0.50037 | 0 | 3 |

| | | | | | | | | | |
|---|---|---|---|---|---|---|---|---|---|
| Lyz2 | 10 | Male | 3 | 0.67444 | 0.64191 | 0.3706 | 0.41204 | 0 | 3 |
| | | Female | 3 | 1.08169 | 0.73922 | 0.42679 | 0.54644 | 0 | 3 |
| | 14 | Male | 3 | 1.30613 | 0.61921 | 0.3575 | 0.38342 | 0 | 3 |
| | | Female | 3 | 2.32319 | 0.22447 | 0.1296 | 0.05039 | 0 | 3 |
| cKO COX-2 | 10 | Male | 3 | 0.39046 | 0.06992 | 0.04037 | 0.00489 | 0 | 3 |
| | | Female | 3 | 0.20217 | 0.23421 | 0.13522 | 0.05485 | 0 | 3 |
| | 14 | Male | 3 | 0.35946 | 0.08154 | 0.04708 | 0.00665 | 0 | 3 |
| | | Female | 3 | 0.25954 | 0.31262 | 0.18049 | 0.09773 | 0 | 3 |

*ANOVA*

*Overall ANOVA*

| | DF | Sum of Squares | Mean Square | F Value | P Value |
|---|---|---|---|---|---|
| Genotype | 2 | 7.12158 | 3.56079 | 15.43678 | <0.0001 |
| Timepoint | 1 | 0.98569 | 0.98569 | 4.27317 | 0.04967 |
| Gender | 1 | 0.46113 | 0.46113 | 1.99912 | 0.17023 |
| Genotype*Timepoint | 2 | 1.65201 | 0.826 | 3.5809 | 0.04356 |
| Genotype*Gender | 2 | 1.15964 | 0.57982 | 2.51365 | 0.10206 |
| Timepoint*Gender | 1 | 0.13592 | 0.13592 | 0.58925 | 0.4502 |
| Genotype*Timepoint*Gender | 2 | 0.14998 | 0.07499 | 0.32511 | 0.72558 |
| Model | 11 | 11.66597 | 1.06054 | 4.59767 | 8.70565E-4 |
| Error | 24 | 5.53606 | 0.23067 | | |
| Corrected Total | 35 | 17.20203 | | | |

At the 0.05 level, the population means of **Genotype** are **significantly** different.
At the 0.05 level, the population means of **Timepoint** are **significantly** different.
At the 0.05 level, the population means of **Gender** are **not significantly** different.
At the 0.05 level, the population means of **Genotype*Timepoint** are **significantly** different.
At the 0.05 level, the population means of **Genotype*Gender** are **not significantly** different.
At the 0.05 level, the population means of **Timepoint*Gender** are **not significantly** different.
At the 0.05 level, the population means of **Genotype*Timepoint*Gender** are **not significantly** different.

*Means Comparisons Tukey Test*

*Genotype*

| | MeanDiff | SEM | q Value | Prob | Alpha | Sig | LCL | UCL |
|---|---|---|---|---|---|---|---|---|
| WT Lyz2 | -0.25043 | 0.16009 | -2.21223 | 0.28011 | 0.05 | 0 | -0.65023 | 0.14936 |
| WT cKO COX-2 | 0.79302 | 0.16009 | 7.0053 | 1.34047E-4 | 0.05 | 1 | 0.39323 | 1.19282 |
| Lyz2 cKO COX-2 | 1.04345 | 0.16009 | 9.21753 | <0.0001 | 0.05 | 1 | 0.64366 | 1.44325 |

*Timepoint*

| | MeanDiff | SEM | q Value | Prob | Alpha | Sig | LCL | UCL |
|---|---|---|---|---|---|---|---|---|
| 10 14 | -0.33094 | 0.13072 | -3.58043 | 0.01831 | 0.05 | 1 | -0.60073 | -0.06115 |

*Gender*

| | MeanDiff | SEM | q Value | Prob | Alpha | Sig | LCL | UCL |
|---|---|---|---|---|---|---|---|---|
| Male Female | -0.22636 | 0.13072 | -2.44895 | 0.09617 | 0.05 | 0 | -0.49614 | 0.04343 |

*Genotype 's*

| | Mean | Groups | |
|---|---|---|---|
| Lyz2 | 1.34636 | A | |
| WT | 1.09593 | A | |
| cKO COX-2 | 0.30291 | | B |

Means that do not share a letter are significantly different.

*Timepoint 's*

| | Mean | Groups | |
|---|---|---|---|
| 14 | 1.08054 | A | |
| 10 | 0.7496 | | B |

Means that do not share a letter are significantly different.

*Gender 's Gr*

| | Mean | Groups |
|---|---|---|
| Female | 1.02825 | A |
| Male | 0.80189 | A |

Means that do not share a letter are significantly different.

*Interactions '*

| Genotype | Timepoint | Gender | Mean | Groups | |
|---|---|---|---|---|---|
| Lyz2 | 14 | Female | 2.32319 | A | |
| Lyz2 | 14 | Male | 1.30613 | A | B |
| WT | 14 | Female | 1.18275 | A | B |
| WT | 10 | Female | 1.12013 | | B |
| Lyz2 | 10 | Female | 1.08169 | | B |
| WT | 14 | Male | 1.05215 | | B |
| WT | 10 | Male | 1.0287 | | B |
| Lyz2 | 10 | Male | 0.67444 | | B |
| cKO COX-2 | 10 | Male | 0.39046 | | B |
| cKO COX-2 | 14 | Male | 0.35946 | | B |
| cKO COX-2 | 14 | Female | 0.25954 | | B |
| cKO COX-2 | 10 | Female | 0.20217 | | B |

Means that do not share a letter are significantly different.

Sig equals 1 indicates that the difference of the means is significant at the 0.05 level. Sig equals 0 indicates that the difference of the means is not significant at the 0.05 level.

*ANOVAThreeWay (11/5/2024 18:25:2*

*Descriptive Statistics Genotype*

| | N | Mean | SD | SEM | Variance | Missing | NonMissing |
|---|---|---|---|---|---|---|---|
| WT | 12 | 1.18721 | 0.74551 | 0.21521 | 0.55579 | 0 | 12 |
| Lyz2 | 12 | 1.61116 | 0.97374 | 0.2811 | 0.94817 | 0 | 12 |
| cKO COX-2 | 12 | 0.48816 | 0.46648 | 0.13466 | 0.2176 | 0 | 12 |

*Timepoint*

| | N | Mean | SD | SEM | Variance | Missing | NonMissing |
|---|---|---|---|---|---|---|---|
| 10 | 18 | 0.86107 | 0.75595 | 0.17818 | 0.57146 | 0 | 18 |
| 14 | 18 | 1.32995 | 0.93814 | 0.22112 | 0.88012 | 0 | 18 |

*Gender*

| | N | Mean | SD | SEM | Variance | Missing | NonMissing |
|---|---|---|---|---|---|---|---|
| Male | 18 | 1.24244 | 0.93186 | 0.21964 | 0.86836 | 0 | 18 |
| Female | 18 | 0.94858 | 0.80863 | 0.1906 | 0.65389 | 0 | 18 |

*Genotype*Timepoint*

| | | N | Mean | SD | SEM | Variance | Missing | NonMissing |
|---|---|---|---|---|---|---|---|---|
| WT | 10 | 6 | 1.27176 | 0.94591 | 0.38617 | 0.89475 | 0 | 6 |
| | 14 | 6 | 1.10266 | 0.55752 | 0.22761 | 0.31083 | 0 | 6 |
| Lyz2 | 10 | 6 | 1.01912 | 0.60747 | 0.248 | 0.36901 | 0 | 6 |
| | 14 | 6 | 2.20321 | 0.9358 | 0.38204 | 0.87573 | 0 | 6 |
| cKO COX-2 | 10 | 6 | 0.29232 | 0.24217 | 0.09886 | 0.05865 | 0 | 6 |
| | 14 | 6 | 0.68399 | 0.57275 | 0.23382 | 0.32804 | 0 | 6 |

*Genotype*Gender*

| | | N | Mean | SD | SEM | Variance | Missing | NonMissing |
|---|---|---|---|---|---|---|---|---|
| WT | Male | 6 | 1.12367 | 0.58819 | 0.24013 | 0.34597 | 0 | 6 |
| | Female | 6 | 1.25075 | 0.93117 | 0.38015 | 0.86707 | 0 | 6 |
| Lyz2 | Male | 6 | 1.83123 | 1.29216 | 0.52752 | 1.66968 | 0 | 6 |
| | Female | 6 | 1.39109 | 0.54779 | 0.22363 | 0.30007 | 0 | 6 |
| cKO COX-2 | Male | 6 | 0.77244 | 0.48863 | 0.19948 | 0.23875 | 0 | 6 |
| | Female | 6 | 0.20388 | 0.21453 | 0.08758 | 0.04602 | 0 | 6 |

*Timepoint*Gender*

| | | N | Mean | SD | SEM | Variance | Missing | NonMissing |
|---|---|---|---|---|---|---|---|---|
| 10 | Male | 9 | 0.74906 | 0.45436 | 0.15145 | 0.20644 | 0 | 9 |
| | Female | 9 | 0.97307 | 0.98979 | 0.32993 | 0.97968 | 0 | 9 |
| 14 | Male | 9 | 1.73583 | 1.04456 | 0.34819 | 1.09111 | 0 | 9 |
| | Female | 9 | 0.92408 | 0.63912 | 0.21304 | 0.40848 | 0 | 9 |

*Genotype*Timepoint*Gender*

| | | | N | Mean | SD | SEM | Variance | Missing | NonMissing |
|---|---|---|---|---|---|---|---|---|---|
| WT | 10 | Male | 3 | 1.06965 | 0.43504 | 0.25117 | 0.18926 | 0 | 3 |
| | | Female | 3 | 1.47387 | 1.38747 | 0.80106 | 1.92507 | 0 | 3 |
| | 14 | Male | 3 | 1.17769 | 0.81665 | 0.47149 | 0.66692 | 0 | 3 |
| | | Female | 3 | 1.02763 | 0.30539 | 0.17632 | 0.09327 | 0 | 3 |

| | | | | | | | | | |
|---|---|---|---|---|---|---|---|---|---|
| Lyz2 | 10 | Male | 3 | 0.78432 | 0.46994 | 0.27132 | 0.22084 | 0 | 3 |
| | | Female | 3 | 1.25392 | 0.73232 | 0.42281 | 0.5363 | 0 | 3 |
| | 14 | Male | 3 | 2.87814 | 0.81564 | 0.47091 | 0.66526 | 0 | 3 |
| | | Female | 3 | 1.52827 | 0.39678 | 0.22908 | 0.15743 | 0 | 3 |
| cKO COX-2 | 10 | Male | 3 | 0.39321 | 0.26399 | 0.15242 | 0.06969 | 0 | 3 |
| | | Female | 3 | 0.19143 | 0.21537 | 0.12435 | 0.04639 | 0 | 3 |
| | 14 | Male | 3 | 1.15166 | 0.30946 | 0.17867 | 0.09577 | 0 | 3 |
| | | Female | 3 | 0.21633 | 0.26116 | 0.15078 | 0.06821 | 0 | 3 |

*ANOVA*

*Overall ANOVA*

| | DF | Sum of Squares | Mean Square | F Value | P Value |
|---|---|---|---|---|---|
| Genotype | 2 | 7.71818 | 3.85909 | 9.78141 | 7.81884E-4 |
| Timepoint | 1 | 1.9787 | 1.9787 | 5.0153 | 0.03466 |
| Gender | 1 | 0.77723 | 0.77723 | 1.97 | 0.17325 |
| Genotype*Timepoint | 2 | 2.77351 | 1.38675 | 3.51492 | 0.04583 |
| Genotype*Gender | 2 | 0.82215 | 0.41107 | 1.04193 | 0.36819 |
| Timepoint*Gender | 1 | 2.41383 | 2.41383 | 6.1182 | 0.02085 |
| Genotype*Timepoint*Gender | 2 | 0.70303 | 0.35151 | 0.89096 | 0.4234 |
| Model | 11 | 17.18663 | 1.56242 | 3.96018 | 0.00233 |
| Error | 24 | 9.46879 | 0.39453 | | |
| Corrected Total | 35 | 26.65542 | | | |

At the 0.05 level, the population means of **Genotype** are **significantly** different.
At the 0.05 level, the population means of **Timepoint** are **significantly** different.
At the 0.05 level, the population means of **Gender** are **not significantly** different.
At the 0.05 level, the population means of **Genotype*Timepoint** are **significantly** different.
At the 0.05 level, the population means of **Genotype*Gender** are **not significantly** different.
At the 0.05 level, the population means of **Timepoint*Gender** are **significantly** different.
At the 0.05 level, the population means of **Genotype*Timepoint*Gender** are **not significantly** different.

*Means Comparisons  Tukey Test*

*Genotype*

| | MeanDiff | SEM | q Value | Prob | Alpha | Sig | LCL | UCL |
|---|---|---|---|---|---|---|---|---|
| WT Lyz2 | -0.42395 | 0.20937 | -2.86359 | 0.12787 | 0.05 | 0 | -0.94681 | 0.09891 |
| WT cKO COX-2 | 0.69905 | 0.20937 | 4.72176 | 0.00744 | 0.05 | 1 | 0.17619 | 1.22191 |
| Lyz2 cKO COX-2 | 1.123 | 0.20937 | 7.58535 | <0.0001 | 0.05 | 1 | 0.60014 | 1.64586 |

*Timepoint*

| | MeanDiff | SEM | q Value | Prob | Alpha | Sig | LCL | UCL |
|---|---|---|---|---|---|---|---|---|

| | | | | | | | | |
|---|---|---|---|---|---|---|---|---|
| 10 14 | -0.46889 | 0.17095 | -3.8789 | 0.01133 | 0.05 | 1 | -0.82172 | -0.11606 |

*Gender*

| | MeanDiff | SEM | q Value | Prob | Alpha | Sig | LCL | UCL |
|---|---|---|---|---|---|---|---|---|
| Male Female | 0.29387 | 0.17095 | 2.43105 | 0.09849 | 0.05 | 0 | -0.05896 | 0.6467 |

*Genotype 's*

| | Mean | Groups | |
|---|---|---|---|
| Lyz2 | 1.61116 | A | |
| WT | 1.18721 | A | |
| cKO COX-2 | 0.48816 | | B |

Means that do not share a letter are significantly different.

*Timepoint 's*

| | Mean | Groups | |
|---|---|---|---|
| 14 | 1.32995 | A | |
| 10 | 0.86107 | | B |

Means that do not share a letter are significantly different.

*Gender 's Gr*

| | Mean | Groups |
|---|---|---|
| Male | 1.24244 | A |
| Female | 0.94858 | A |

Means that do not share a letter are significantly different.

*Interactions '*

| Genotype | Timepoint | Gender | Mean | Groups | |
|---|---|---|---|---|---|
| Lyz2 | 14 | Male | 2.87814 | A | |
| Lyz2 | 14 | Female | 1.52827 | A | B |
| WT | 10 | Female | 1.47387 | A | B |
| Lyz2 | 10 | Female | 1.25392 | | B |
| WT | 14 | Male | 1.17769 | | B |
| cKO COX-2 | 14 | Male | 1.15166 | | B |
| WT | 10 | Male | 1.06965 | | B |
| WT | 14 | Female | 1.02763 | | B |
| Lyz2 | 10 | Male | 0.78432 | | B |
| cKO COX-2 | 10 | Male | 0.39321 | | B |
| cKO COX-2 | 14 | Female | 0.21633 | | B |
| cKO COX-2 | 10 | Female | 0.19143 | | B |

Means that do not share a letter are significantly different.

Sig equals 1 indicates that the difference of the means is significant at the 0.05 level. Sig equals 0 indicates that the difference of the means is not significant at the 0.05 level.

*ANOVAThreeWay (11/5/2024 18:43:4*

*Descriptive Statistics  Genotype*

| | N | Mean | SD | SEM | Variance | Missing | NonMissing |
|---|---|---|---|---|---|---|---|
| WT | 16 | 1.19402 | 0.75571 | 0.21815 | 0.5711 | 4 | 12 |
| Lyz2 | 15 | 0.95898 | 0.77993 | 0.22515 | 0.60829 | 3 | 12 |
| cKO COX-2 | 14 | 0.36538 | 0.30428 | 0.08439 | 0.09259 | 1 | 13 |

*Timepoint*

| | N | Mean | SD | SEM | Variance | Missing | NonMissing |
|---|---|---|---|---|---|---|---|
| 10 | 23 | 0.92598 | 0.78138 | 0.17926 | 0.61055 | 4 | 19 |
| 14 | 22 | 0.72179 | 0.65551 | 0.1545 | 0.42969 | 4 | 18 |

*Gender*

| | N | Mean | SD | SEM | Variance | Missing | NonMissing |
|---|---|---|---|---|---|---|---|
| Male | 23 | 0.74473 | 0.5807 | 0.13322 | 0.33721 | 4 | 19 |
| Female | 22 | 0.91311 | 0.85227 | 0.20088 | 0.72636 | 4 | 18 |

*Genotype*Timepoint*

| | | N | Mean | SD | SEM | Variance | Missing | NonMissing |
|---|---|---|---|---|---|---|---|---|
| WT | 10 | 9 | 1.15743 | 0.71119 | 0.29034 | 0.50579 | 3 | 6 |
| | 14 | 7 | 1.23061 | 0.86453 | 0.35294 | 0.74741 | 1 | 6 |
| Lyz2 | 10 | 6 | 1.20338 | 1.06481 | 0.43471 | 1.13382 | 0 | 6 |
| | 14 | 9 | 0.71458 | 0.24713 | 0.10089 | 0.06107 | 3 | 6 |
| cKO COX-2 | 10 | 8 | 0.48984 | 0.33595 | 0.12698 | 0.11286 | 1 | 7 |
| | 14 | 6 | 0.22018 | 0.19947 | 0.08143 | 0.03979 | 0 | 6 |

*Genotype*Gender*

| | | N | Mean | SD | SEM | Variance | Missing | NonMissing |
|---|---|---|---|---|---|---|---|---|
| WT | Male | 8 | 1.12897 | 0.64298 | 0.2625 | 0.41342 | 2 | 6 |
| | Female | 8 | 1.25907 | 0.9126 | 0.37257 | 0.83284 | 2 | 6 |
| Lyz2 | Male | 8 | 0.86059 | 0.49034 | 0.20018 | 0.24044 | 2 | 6 |
| | Female | 7 | 1.05736 | 1.03662 | 0.4232 | 1.07457 | 1 | 6 |
| cKO COX-2 | Male | 7 | 0.31607 | 0.30217 | 0.11421 | 0.09131 | 0 | 7 |
| | Female | 7 | 0.42291 | 0.32444 | 0.13245 | 0.10526 | 1 | 6 |

*Timepoint*Gender*

| | | N | Mean | SD | SEM | Variance | Missing | NonMissing |
|---|---|---|---|---|---|---|---|---|
| 10 | Male | 11 | 0.77992 | 0.71408 | 0.22581 | 0.50991 | 1 | 10 |
| | Female | 12 | 1.08827 | 0.86243 | 0.28748 | 0.74378 | 3 | 9 |
| 14 | Male | 12 | 0.70563 | 0.42638 | 0.14213 | 0.1818 | 3 | 9 |
| | Female | 10 | 0.73795 | 0.85481 | 0.28494 | 0.7307 | 1 | 9 |

*Genotype*Timepoint*Gender*

| | | | N | Mean | SD | SEM | Variance | Missing | NonMissing |
|---|---|---|---|---|---|---|---|---|---|
| WT | 10 | Male | 4 | 1.1971 | 0.921 | 0.53174 | 0.84823 | 1 | 3 |
| | | Female | 5 | 1.11775 | 0.6415 | 0.37037 | 0.41153 | 2 | 3 |
| | 14 | Male | 4 | 1.06083 | 0.414 | 0.23902 | 0.17139 | 1 | 3 |
| | | Female | 3 | 1.40038 | 1.26912 | 0.73272 | 1.61066 | 0 | 3 |

| | | | | | | | | | |
|---|---|---|---|---|---|---|---|---|---|
| Lyz2 | 10 | Male | 3 | 0.96334 | 0.73226 | 0.42277 | 0.5362 | 0 | 3 |
| | | Female | 3 | 1.44341 | 1.45791 | 0.84172 | 2.12549 | 0 | 3 |
| | 14 | Male | 5 | 0.75784 | 0.18226 | 0.10523 | 0.03322 | 2 | 3 |
| | | Female | 4 | 0.67131 | 0.33741 | 0.1948 | 0.11385 | 1 | 3 |
| cKO COX-2 | 10 | Male | 4 | 0.32947 | 0.35854 | 0.17927 | 0.12855 | 0 | 4 |
| | | Female | 4 | 0.70366 | 0.16044 | 0.09263 | 0.02574 | 1 | 3 |
| | 14 | Male | 3 | 0.29821 | 0.28331 | 0.16357 | 0.08026 | 0 | 3 |
| | | Female | 3 | 0.14216 | 0.03074 | 0.01775 | 9.45204E-4 | 0 | 3 |

*ANOVA*

*Overall ANOVA*

| | DF | Sum of Squares | Mean Square | F Value | P Value |
|---|---|---|---|---|---|
| Genotype | 2 | 4.51737 | 2.25869 | 4.59057 | 0.02004 |
| Timepoint | 1 | 0.51773 | 0.51773 | 1.05225 | 0.31481 |
| Gender | 1 | 0.19408 | 0.19408 | 0.39446 | 0.53566 |
| Genotype*Timepoint | 2 | 0.4901 | 0.24505 | 0.49804 | 0.61362 |
| Genotype*Gender | 2 | 0.01284 | 0.00642 | 0.01304 | 0.98705 |
| Timepoint*Gender | 1 | 0.11734 | 0.11734 | 0.23849 | 0.62956 |
| Genotype*Timepoint*Gender | 2 | 0.47288 | 0.23644 | 0.48055 | 0.62404 |
| Model | 11 | 6.37924 | 0.57993 | 1.17866 | 0.34952 |
| Error | 25 | 12.30068 | 0.49203 | | |
| Corrected Total | 36 | 18.67993 | | | |

At the 0.05 level, the population means of **Genotype** are **significantly** different.
At the 0.05 level, the population means of **Timepoint** are **not significantly** different.
At the 0.05 level, the population means of **Gender** are **not significantly** different.
At the 0.05 level, the population means of **Genotype*Timepoint** are **not significantly** different.
At the 0.05 level, the population means of **Genotype*Gender** are **not significantly** different.
At the 0.05 level, the population means of **Timepoint*Gender** are **not significantly** different.
At the 0.05 level, the population means of **Genotype*Timepoint*Gender** are **not significantly** different.

*Means Comparisons  Tukey Test*

*Genotype*

| | MeanDiff | SEM | q Value | Prob | Alpha | Sig | LCL | UCL |
|---|---|---|---|---|---|---|---|---|
| WT Lyz2 | 0.23504 | 0.23539 | 1.4121 | 0.58461 | 0.05 | 0 | -0.35127 | 0.82135 |
| WT cKO COX-2 | 0.82564 | 0.23168 | 5.03979 | 0.00415 | 0.05 | 1 | 0.24857 | 1.40272 |
| Lyz2 cKO COX-2 | 0.5906 | 0.23168 | 3.6051 | 0.04414 | 0.05 | 1 | 0.01353 | 1.16768 |

*Timepoint*

| | MeanDiff | SEM | q Value | Prob | Alpha | Sig | LCL | UCL |
|---|---|---|---|---|---|---|---|---|
| 10 14 | 0.23733 | 0.19018 | 1.76484 | 0.22362 | 0.05 | 0 | -0.15436 | 0.62902 |

*Gender*

| | MeanDiff | SEM | q Value | Prob | Alpha | Sig | LCL | UCL |
|---|---|---|---|---|---|---|---|---|
| Male Female | -0.14531 | 0.19018 | -1.08055 | 0.45198 | 0.05 | 0 | -0.537 | 0.24638 |

*Genotype ’s*

| | Mean | Groups | |
|---|---|---|---|
| WT | 1.19402 | A | |
| Lyz2 | 0.95898 | A | |
| cKO COX-2 | 0.36538 | | B |

Means that do not share a letter are significantly different.

*Timepoint ’s*

| | Mean | Groups |
|---|---|---|
| 10 | 0.92598 | A |
| 14 | 0.72179 | A |

Means that do not share a letter are significantly different.

*Gender ’s Gr*

| | Mean | Groups |
|---|---|---|
| Female | 0.91311 | A |
| Male | 0.74473 | A |

Means that do not share a letter are significantly different.

*Interactions ’*

| Genotype | Timepoint | Gender | Mean | Groups |
|---|---|---|---|---|
| Lyz2 | 10 | Female | 1.44341 | A |
| WT | 14 | Female | 1.40038 | A |
| WT | 10 | Male | 1.1971 | A |
| WT | 10 | Female | 1.11775 | A |
| WT | 14 | Male | 1.06083 | A |
| Lyz2 | 10 | Male | 0.96334 | A |
| Lyz2 | 14 | Male | 0.75784 | A |
| cKO COX-2 | 10 | Female | 0.70366 | A |
| Lyz2 | 14 | Female | 0.67131 | A |
| cKO COX-2 | 10 | Male | 0.32947 | A |
| cKO COX-2 | 14 | Male | 0.29821 | A |
| cKO COX-2 | 14 | Female | 0.14216 | A |

Means that do not share a letter are significantly different.

Sig equals 1 indicates that the difference of the means is significant at the 0.05 level. Sig equals 0 indicates that the difference of the means is not significant at the 0.05 level.
